# Colonized Niche, Evolution and Function Signatures of *Bifidobacterium pseudolongum* within Bifidobacterial Genus

**DOI:** 10.3390/foods10102284

**Published:** 2021-09-27

**Authors:** Yue Xiao, Jianxin Zhao, Hao Zhang, Qixiao Zhai, Wei Chen

**Affiliations:** 1 State Key Laboratory of Food Science and Technology, Jiangnan University, Wuxi 214122, China; 7170112038@stu.jiangnan.edu.cn (Y.X.); jxzhao@jiangnan.edu.cn (J.Z.); zhanghao@jiangnan.edu.cn (H.Z.); chenwei66@jiangnan.edu.cn (W.C.); 2 School of Food Science and Technology, Jiangnan University, Wuxi 214122, China; 3 National Engineering Research Center for Functional Food, Jiangnan University, Wuxi 214122, China; 4 Institute of Food Biotechnology, Jiangnan University, Yangzhou 225004, China; 5 Wuxi Translational Medicine Research Center, Jiangsu Translational Medicine Research Institute Wuxi Branch, Wuxi 214122, China; 6 International Joint Research Laboratory for Probiotics, Jiangnan University, Wuxi 214122, China

**Keywords:** *Bifidobacterium*, *Bifidobacterium pseudolongum*, population genomics, niche, evolution, probiotic effector molecules

## Abstract

Background: Although genomic features of various bifidobacterial species have received much attention in the past decade, information on *Bifidobacterium pseudolongum* was limited. In this study, we retrieved 887 publicly available genomes of bifidobacterial species, and tried to elucidate phylogenetic and potential functional roles of *B. pseudolongum* within the *Bifidobacterium* genus. Results: The results indicated that *B. pseudolongum* formed a population structure with multiple monophyletic clades, and had established associations with different types of mammals. The abundance of *B. pseudolongum* was inversely correlated with that of the harmful gut bacterial taxa. We also found that *B. pseudolongum* showed a strictly host-adapted lifestyle with a relatively smaller genome size, and higher intra-species genetic diversity in comparison with the other tested bifidobacterial species. For functional aspects, *B. pseudolongum* showed paucity of specific metabolic functions, and enrichment of specific enzymes degrading complex plant carbohydrates and host glycans. In addition, *B. pseudolongum* possessed a unique signature of probiotic effector molecules compared with the other tested bifidobacterial species. The investigation on intra-species evolution of *B. pseudolongum* indicated a clear evolution trajectory in which considerable clade-specific genes, and variation on genomic diversity by clade were observed. Conclusions: These findings provide valuable information for explaining the host adaptability of *B. pseudolongum*, its evolutionary role, as well as its potential probiotic effects.

## 1. Introduction

Bifidobacteria are important colonizers in the mammalian gut, and abundance of the genus is considered to be positively correlated with host’s health outcomes [1,2,3]. Bifidobacterial composition in the gut has been studied by using isolation, denaturing gradient gel electrophoresis (DGGE) and internally transcribed spacer (ITS) rRNA profiling methods [4,5,6,7]. Recently, bifidobacterial composition among a wide range of 291 adult animals was analyzed, indicating their widespread distribution across the mammalian kingdom [7]. In this context, *B. pseudolongum*, together with *B. longum* and *B. adolescentis*, was shown to be the most predominant bifidobacterial species present in the gut of various mammals. Multiple bifidobacterial species were reported to coexist in the gut [7], showing species-level diversity. *B. pseudolongum* were enriched in the gut of some kinds of mammals (e.g., animals from Erinaceidae and Canidae), while were in low abundance for others (e.g., animals from Cebidae and Cercopithecidae). Notably, Mao et al. previously showed that *B. pseudolongum* was the exclusive bifidobacterial species detected in the gut of Balb/c mice [8].

There are 72 currently recognized (sub)species within the *Bifidobacterium* genus. *B. pseudolongum*, as a species of the *Bifidobacterium* genus, consists of two subspecies, *B. pseudolongum* subsp. *pseudolongum* and *B. pseudolongum* subsp. *globosum* [9]. *B. pseudolongum* subsp. *pseudolongum* was first isolated and described by Mitsuoka in 1969 [10]. *B*. *globosum* was isolated from bovine rumen by Scardovi in the same year [11], and further classified into a subspecies of *B. pseudolongum*, that is, *pseudolongum* subsp. *globosum* in 1992 [9]. *B*. *pseudolongum* is a widely distributed gut commensal of the animal kingdom, and can be isolated from the guts of carnivores, herbivores, birds, and reptiles [12]. Nowadays, although comparative genomic analysis among various bifidobacterial species have been conducted [13,14,15,16,17], little attention has been paid to *B. pseudolongum*. Although the phylogenetic structure and metabolic ability of the species have been partially revealed by Lugli [12], the intra-species evolution, the subspecies-specific features, and the phylogenetic and potential functional roles of *B. pseudolongum* within the *Bifidobacterium* genus need further investigation.

Some bifidobacterial species, such as *B. longum*, *B. animalis*, *B. breve* and *B. bifidum*, have been extensively investigated and validated for their uses as probiotics [18,19,20,21,22]. However, very limited studies have focused on probiotic properties of *B. pseudolongum*. *B. pseudolongum* was evaluated in vitro for its potential probiotic functionality [23,24]. Five *B. pseudolongum* strains isolated from rats were evaluated for their tolerance to low pH and bile, as well as their capacity to adhere to intestinal epithelial cells and mucus [23]. The *B. pseudolongum* strain 119 has been tested for its growth rate, aerotolerance, antagonistic activity against pathogens, antimicrobial susceptibility profile, and cell wall hydrophobicity [24]. Several in vivo studies have also been conducted to support the potential beneficial effects of *B. pseudolongum* on the host. The *B. pseudolongum* strain Patronus has been reported to alleviate oxidative damage in metronidazole-treated rats [25], and in a previous study, oral administration of an isolated *B. pseudolongum* strain (no strain-specific full name of the strain was provided) was found to protect against hypersensitivity in mice [26]. Similarly, the distribution of genomic features that might contribute to probiotic effects, such as mucin-glycan degrading enzymes, pili, and S-layer protein, has been revealed in some probiotic bifidobacterial species [27,28], but remains overlooked in *B. pseudolongum*.

In this study, we analyzed the bifidobacterial composition and bacterial structure of the gut microbiota of representative mammals to explore niche features of *B. pseudolongum* and identify specific bacterial taxa whose abundances were correlated with that of *B. pseudolongum*. We analyzed 887 bifidobacterial genomes and their niche information, and constructed species-level phylogenetic trees for the selected bifidobacterial species. We also evaluated the phylogenetic role of *B. pseudolongum* within the *Bifidobacterium* genus by comparing the phylogenetic relationship, general genomic features, and genomic diversity among different bifidobacterial species. The profiles of carbohydrate-utilizing enzymes and signatures of clusters of orthologous groups of protein (COG) functions were also compared between *B. pseudolongum* and the other selected bifidobacterial species. In addition, we analyzed the distribution of genes encoding potential probiotic effectors. Finally, we explored the intra-species evolution and the clade-specific genomic features of *B. pseudolongum*.

## 2. Materials and Methods

### 2.1. Sample Collection

Fecal samples of C57/B6 male mice (8-week-old, *n* = 20), Balb/c male mice (8-week-old, *n* = 20), Wistar female rats (8-week-old, *n* = 22) and human subjects (*n* = 60) were collected to determine microbial composition. The mice were bred in the Laboratory Animal Center of the Department of Food Science and Technology, Jiangnan University, Wuxi, China, under specific pathogen-free (SPF) conditions for 3 weeks before sample collection. All of the study protocols were approved by the Ethics Committee of Jiangnan University, China (JN. No20181130b1200130[261]). All of the applicable institutional and national guidelines (including the ARRIVE guidelines, the UK Animals (Scientific Procedures) Act, 1986 and associated guidelines, EU Directive 2010/63/EU for animal experiments, and the National Institutes of Health guide for the care and use of Laboratory animals (NIH Publications No. 8023, revised 1978)) for the care and use of animals were followed. The human subjects were recruited from the provinces of Qinhai (*n* = 20), Heilongjiang (*n* = 20), and Jilin (*n* = 20), with a median age of the cohort of 48 and a ratio of female/male of 27/32 (information of one sample was missing). The human subjects were self-reported to be healthy and had not consumed antibiotics or probiotic-based products for one month before fecal sample collection.

### 2.2. Analysis of Fecal Microbial Community and Composition of Bifidobacterium Species

The microbial DNA of fecal samples were extracted by using the MP FastDNA Spin kit for Feces (MP Biomedicals) according to the manual. The V3-V4 region of 16S rRNA and the 60 kDa chaperonin (groEL) gene were amplified using barcoded fusion primers, and then sequenced with a Miseq TM sequencer separately for determination of microbial community and species-level *Bifidobacterium* composition, as previously described [29]. After sequencing, 16S rRNA sequencing data were analyzed using the QIIME pipeline [30]. For determination of bifidobacterial composition, a local nucleotide database was constructed using Bioedit, and the BLASTn algorithm was used to count the numbers of sequences that belong to each individual species. The PCA analysis based on abundances of key gut bacteria was conducted by the prcomp function and visualized using the ggbiplot package in R software. For correlation analysis, Spearman’s correlation with FDR correlation was conducted by using package psych in R. The correlation coefficients of <−0.5 or >0.5, and *p* < 0.05 were considered as statistically significant.

### 2.3. Phylogenetic Reconstruction, Hierarchical Bayesian Clustering, Fixation Index (Fst) Caculation, Assembly Re-Annotation, and Pan-Genome Analysis

We retrieved 887 publicly available genomes/strains of the Bifidobacterial species from the NCBI database. The general genomic features (Guanine-Cytosine [GC] content, genome sizes, and numbers of coding sequences [CDs]) and biosample information of these 887 strains were also collected (Table S1). As previously mentioned [31], SNPs of the included genome assemblies were identified via sequence alignment to the corresponding reference genome for each *Bifidobacterium* species via MUMmer [32]. The phylogenetic tree of each *Bifidobacterium* species was constructed by using both the neighbor-joining (NJ) method (via Treebest software) and the maximum-likelihood (ML) method (via FastTree software [33]) based on concatenated sequences of bi-allelic SNPs in the core genome of each corresponding species. Tree-independent hierarchical Bayesian clustering with hierBAPS [34] was used to separately determine the population structure generated from the core genome mapping alignment for each species. For each species with more than two identified populations, SNP sites were used to convert multiple alignments of core-genome to VCF format [35]. Fst values between populations were analyzed by using the R package hierfstat [36]. The genome sequences were re-annotated using Prokka [37], and the annotated results were put into Roary [38] to conduct the pan-genome and gene presence/absence analyses (with a minimum identity of 90%). The core genes of each species were defined by those present in 99% to 100% of the strains of corresponding species. For the phylogenetic reconstruction of the *Bifidobacterium* genus, we selected 10 representative genomes along the tree of each species to cover an intra-species genetic distance for those species with more than 10 publicly available sequenced genomes, and adopted all the genomes for the species with less than 10 publicly available sequenced genomes. After the above selection, a total of 201 strains were adopted, and the homologous genes of the strains were identified by orthomcl [39]. The identified homologous genes were concatenated and then aligned by mafft [40]. The resulted alignment was used to build a NJ tree via the Treebest tool (http://treesoft.sourceforge.net/treebest.shtml, accessed on 25 December 2015). Each above-mentioned tree was visualized and phenotype mapped (e.g., isolated origins and subspecies) by Figtree (http://tree.bio.ed.ac.uk/software/, accessed on 23 January 2018) and iTOL (https://itol.embl.de/, accessed on 22 June 2019).

### 2.4. Profiles of Glucosyltransferase (GTs) and Glycoside HYDROLASES (GHs), and COG Functions

GH and GT genes were predicted among the genomes of 10 *Bifidobacterium* species by using HMMSCAN (from the HMMER package 3.1b2 (http://hmmer.org/, accessed on 30 March 2019)) to query a hidden Markov models-based CAZyme dbCAN database. For protein sequences beyond 80 aa, 50% coverage and an *E*-value < 1 × 10^−5^, and for protein sequences below 80 aa, 50% coverage and an *E*-value < 1 × 10^−3^ were chosen as cutoff thresholds. COG function categories were annotated with protein sequences as inputs by BLASTP against a COG database (ftp://ftp.ncbi.nih.gov/pub/COG/COG2014/data, accessed on 20 May 2019) with a threshold of 45% identity, 50% query coverage, and an *E*-value of 1 × 10^−10^. Genes below the thresholds were not included in the analysis. The PCAs based on profiles of bifidobacterial GTs, and GHs, as well as COG functions were respectively conducted by using the prcomp function and visualized with the ggbiplot package in R software. The heatmap plot showing average gene numbers of COGs or carbohydrate enzymes per strain was drawn by pheatmap packages in R. For comparisons of profiles of carbohydrate-utilizing enzymes and COGs of *B. pseudolongum* by clade, PERMANOVA analysis based on bray distance was used with 999 permutations.

### 2.5. Profiles of Probiotic Effector Factors

For identification of mucin glycan-processing enzymes, the reference protein sequences were collected from the NCBI Refseq database by searching names of the enzymes as keywords (Chitinase (EC 3.2.1.14), neuraminidase/sialidase (EC 3.2.1.18), α-galactosidase (EC 3.2.1.22), β-galactosidase (EC 3.2.1.23), α-*N*-acetylgalactosaminidase (EC 3.2.1.49), α-*N*-acetylglucosaminidase (EC 3.2.1.50), α-l-fucosidase (EC 3.2.1.51), β-*N*-hexosaminidase (EC 3.2.1.52), and endo-α-*N*-acetylgalactosaminidase (EC 3.2.1.97), as reported by Ravcheev et al. [41]). The separate protein databases for other probiotic effectors were built by retrieving sequences from the Refseq database and/or Uniprot database using the keywords “S-layer protein”, “LPXTG”, “sortase”, “pilus, fimbria, and fimbrial protein”, “*luxS*”, “mucus-binding protein”, and “serine-rich glycoprotein adhesin”. The Antimicrobial Peptide Database (APD) was directly adopted (http://aps.unmc.edu/AP/, accessed on 15 April 2019). The amino acid sequences of each genome from Prokka were taken as a query to search against the above reference protein databases by using BLASTP with an E-value of 1 × 10^−5^, sequence identity of 45%, and reference coverage of 50% as the cutoff. For identification of the priming-GTF gene (p-gtf) for EPS biosynthesis, the two genes “undecaprenyl-phosphate sugar phosphotransferase” (rfbP, accession number NP_695455) and “galactosyl-transferase” (cpsD, accession number NP_695447) in *B. longum* NCC2705 were taken as queries separately, as previously reported [42], to search against the proteome of each bifidobacterial strain (amino acid sequences of each bacterial genome from Prokka) with a threshold of query coverage of 50%, *E*-value of 1 × 10^−5^, and sequence identity of 45%.

The BSH genes were identified by searching against the Prokka annotation results using keywords of “choloylglycine hydrolase” or “bile salt hydrolase”, as previously reported [43]. The BSH nucleotide sequences with lengths of 900 bp to 1200 bp were included and aligned by mafft [40], and then a NJ tree was constructed by Treebest (http://treesoft.sourceforge.net/treebest.shtml) based on BSH alignments. The PCA analysis based on probiotic effectors was conducted by the prcomp function and visualized using the ggbiplot package in R software.

### 2.6. Statistical Analysis

For data of normal distribution, a One-Way ANOVA analysis was adopted (Tukey’s b or Tamhane’s T2). Otherwise, the nonparametric Mann-Whitney U test was used. A *p*-value of less than 0.05 was considered to be statistically significant.

## 3. Results

### 3.1. Distribution of B. pseudolongum in the Gut of the Selected Mammals, and Features of B. pseudolongum-Enriched Gut Microbiota

**Figure 1:**
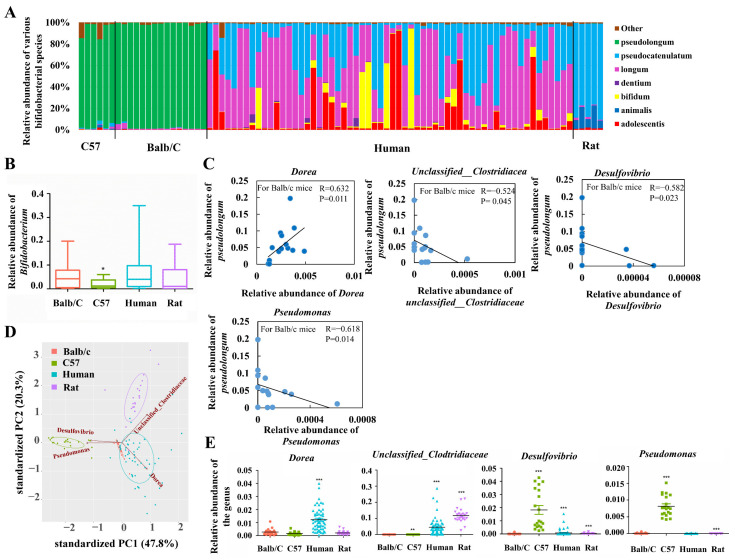
Distribution of *B. pseudolongum* in the gut of selected mammals, and features of *B. pseudolongum*-enriched gut microbiota. (**A**) Bifidobacterial composition in the gut of Balb/c mice (*n* = 15), C57/B6 mice (*n* = 6), humans (*n* = 60) and rats (*n* = 5). Due to good repeatability of sequencing data for C57 mice and rats, only 5 to 6 samples were sequenced. For Balb/c mice, more samples (totally 15) were successfully sequenced in order to conduct the following correlation analysis. For human fecal samples, all the 60 samples were sequenced. (**B**) Relative abundance of the genus *Bifidobacterium*. (**C**) Abundance correlation between relative abundances of *B. pseudolongum* and that of the other genera in the gut of Balb/c mice. Spearman’s method was used for correlation analysis. |R| > 0.5 and *p* < 0.05 (with a total number of 20,000 tests for FDR correction) were considered to be statistically significant. (**D**) PCA plot of the four types of included mammals (Balb/c mice, C57 mice, humans and rats) with the relative abundances of the four representative genera as inputs. (**E**) Relative abundances of the four representative genera. Statistical analysis was conducted between Balb/c group (*n* = 20) and each of the other three groups (C57 group (*n* = 20), human group (*n* = 60) and rat group (*n* = 22)). *, *p* < 0.05; **, *p* < 0.01; ***, *p* < 0.001. All the fecal samples of included humans (60), mice (20 for Balb/c and 20 for C57) and rats (22) were sequenced for 16S rRNA gene data.

Previous studies have proposed better host adaptability of *B. pseudolongum* among various *Bifidobacterium* species by demonstrating its prevalence and dominance across the mammalian tree of life [7]. In order to get closer to its niche features, we sequenced and analyzed the composition of gut bifidobacteria of rodents (Balb/c mice, C57/B6 mice, and Wistar rats) and humans by including more individuals for the given types of animals compared with a previous study [7]. Our results suggested that *B. pseudolongum* was the most dominant species among all bifidobacterial species in Balb/c mice. The dominant colonization of this species was also observed for C57 mice. In contrast, the main bifidobacteria in rats were *B. pseudocatenulatum* and *B. animalis*, while humans were mainly colonized by *B. longum*, *B. pseudocatenulatum*, *B. adolescentis*, and *B. bifidum* with varying relative abundances of these species between individuals. We found that population levels of *Bifidobacterium* in rats, humans, and Balb/c mice were comparable, whereas C57 mice showed significantly lower abundances compared with Balb/c mice (*p* < 0.05, Figure 1B). In general, *B. pseudolongum* was the most dominant bifidobacterial species in the gut of C57 mice and Balb/c mice instead of rats and humans. However, *B. pseudolongum* showed a higher relative colonized biomass in the gut of Balb/c mice compared with C57 mice. Therefore, we concluded that *B. pseudolongum* prefers to expand in the gut of Balb/c mice compared with C57 mice, rats, and humans.

Next, we explored the bacterial taxa whose abundances were correlated positively or negatively with that of *B. pseudolongum* in Balb/c mice (the most potent niche that is enriched with *B. pseudolongum*). After the correlation test, four taxa stood out, including *unclassified__Clostridiaceae*, *Dorea*, *Desulfovibrio*, and *Pseudomonas* (|R| > 0.5 and *p_adj* < 0.05 with a total number of 20,000 tests for FDR correction; Figure 1C). The four kinds of mammals included in this study formed distinct clusters based on the abundances of these four bacterial genera (Figure 1D). In addition, levels of the colonized biomass of these four genera markedly varied between Balb/c mice and each type of the other three kinds of mammals (Figure 1E).

### 3.2. Phylogenetic Structures and Niche Distribution

**Figure 2:**
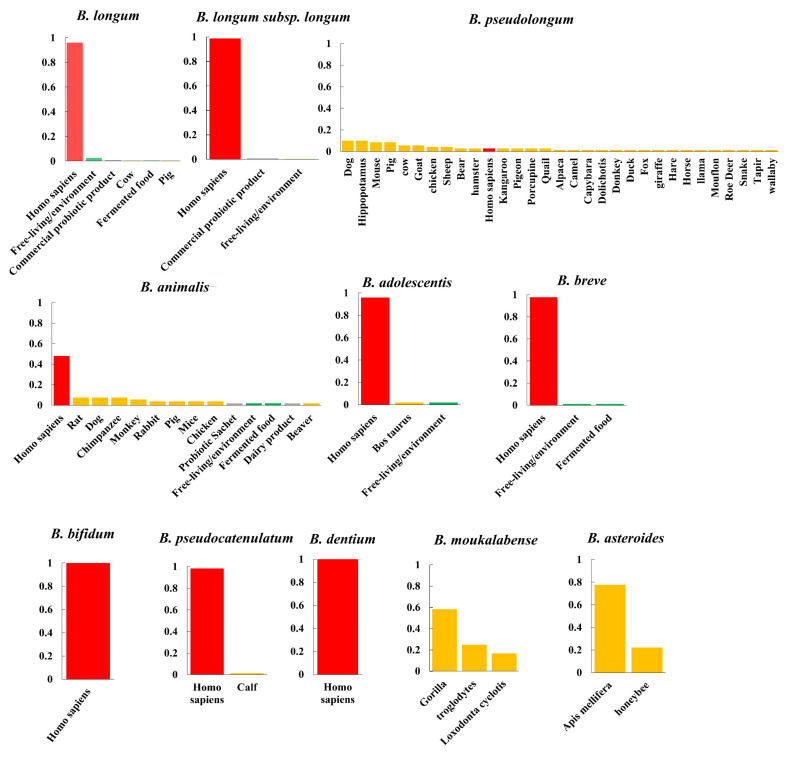
Niche distribution. Niche distribution of bifidobacterial species. The percentage of genomes belonging to the given niche was illustrated. Niches were categorized into human-associated habitats (red), free-living environments, such as food matrices or plants (green), animals (orange), and pure bacterial cultures or commercial supplements for which the isolation origins of microbial strains cannot be determined (gray).

**Figure 3:**
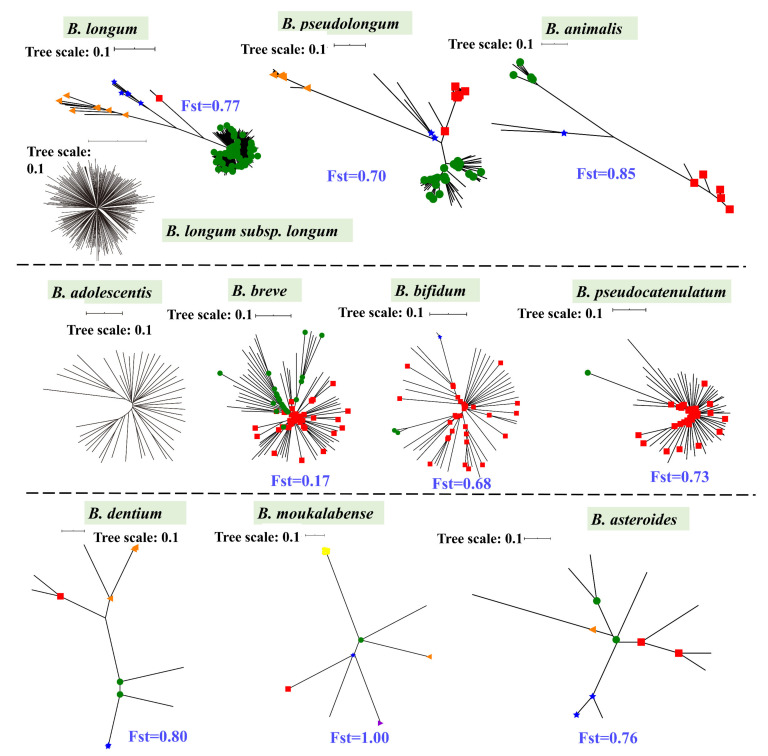
Phylogenetic structures. Phylogenetic structures of bifidobacterial species. The *Bifidobacterium* species with more than 10 publicly available genomes in the NCBI database were selected, and a maximum likelihood (ML) tree was constructed based on bi-SNPs in the core genome of each species. The corresponding NJ trees were also constructed (Figure S1B), and similar topologies as those ML trees are shown. Reference genomes of each species used for tree construction are shown in bold in Table S1. For *B. longum*, population structures of *B. longum* (upper) and *B. longum* subsp. *longum* (lower) are shown. The scale bars for trees were uniformly 0.1. The node symbols represent population clusters based on Bayesian Analysis of Population Structure (BAPS) hierarchical clustering. Within each species, the average Fst values between BAPS populations are also shown.

We analyzed the phylogenetic structures and niche distribution modes of *B. pseudolongum* and nine more *Bifidobacterium* species (each species with a number of publicly available genomes >10) (Figure 2, Figure 3 and Figure S1). We observed that *B. pseudolongum* and *B. animalis*, which showed large associations with a variety of animals, appeared to form multi-clade population structures. In particular, *B. pseudolongum* formed four distinct Bayesian Analysis of Population Structure (BAPS) clusters with an average differentiation value (Fst) of 0.70, and *B. animalis* formed three distinct BAPS clusters with an average Fst of 0.85. In contrast, some of the (sub)species that were frequently found to be associated with humans showed radiating structures (a possible indication of recombination, Figure 3). In particular, *B. longum* subsp. *longum* and *B. adolescentis* showed no genetically distant clusters via BAPS analysis, and two clusters of *B. breve* represented limited population differentiation (Fst = 0.17).

Although the numbers of isolates from particular niches were limited, such as *B. pseudolongum* strains isolated from primates and *B. animalis* strains isolated from Artiodactyla, preliminary host-specific lineages were shown for the two bifidobacterial species (Figure S2A,B). In general, *Bifidobacterium* species, a group of strictly anaerobic bacteria, adopted a host-adapted lifestyle (only occasionally found in free-living niches). *B. pseudolongum* with multi-clade population structures was more frequently isolated from various mammalian animals rather than being mainly found in humans according to the current dataset.

### 3.3. General Genomic Features

**Figure 4:**
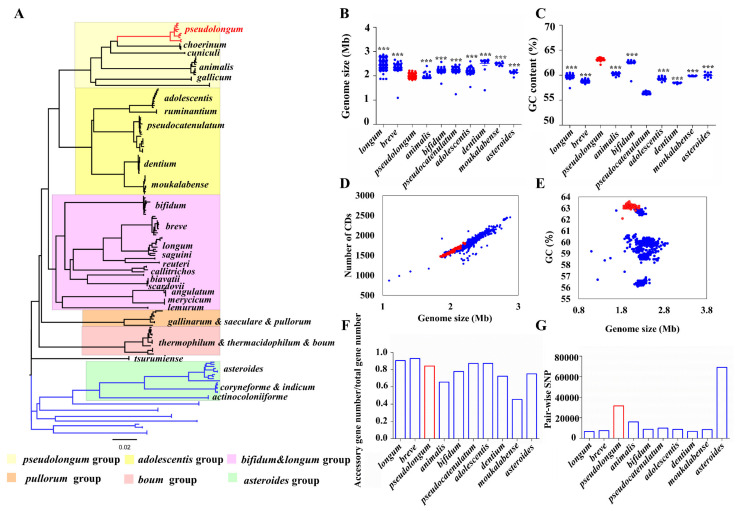
Features of *B. pseudolongum* in terms of phylogenetic role and general genomic features among various *Bifidobacterium* species. (**A**) Phylogenetic tree (NJ tree) of genus *Bifidobacterium* based on the protein sequences of nine orthologs of various species. For species with more than 10 publicly available sequenced genomes, 10 representative strains distributed across the tree, which covered the overall genetic distance of each species, were selected; for those with less than 10 publicly available sequenced genomes, all the strains were included. (**B**,**C**) Genome size (Mb) (**B**) and GC content (%) (**C**) of different species. The statistical analysis was conducted between *B. pseudolongum* and each of the other species. ***, *p* < 0.001. (**D**) Association between genome size (Mb) and the number of CDSs, Pearson *r* = 0.94, *p* < 0.0001. (**E**) Association between genome size (Mb) and GC content (%), Pearson *r* = −0.3, *p* < 0.0001. (**F**,**G**) Comparison of intra-species genomic diversity for each species by accessory genome size (**F**) and SNP distance (**G**). Columns and dots representing *B. pseudolongum* are highlighted in red. All the available 887 sequenced genomes of *Bifidobacterium* in the NCBI database were used for analysis (**A**). Each of the *Bifidobacterium* species with more than 10 publicly available sequenced genomes in the NCBI database were analyzed, resulting in 786 strains in total (**B**–**G**).

To capture the intra-genus evolution of *Bifidobacterium*, we constructed a phylogenetic tree based on core orthologs by selecting representative strains from each species. Six main groups were categorized, including the *B. pseudolongum* group, *B. adolescentis* group, *B. pullorum* group, *B. boum* group, *B. bifidum* and *B. longum* complex, and *B. asteroides* group (Figure 4A). We observed that the *B. asteroides* phylogenetic group was shown to be positioned closest to the root of the tree, therefore indicating that members of this group most closely resemble the evolutionary ancestor of the *Bifidobacterium* genus. In addition, the bootstrap values of the clades were high (Figure S3), suggesting that the root and the phylogenetic structure revealed here were robust. We observed that *B. pseudolongum* belonged to one of the three groups (*B. pseudolongum* group, *B. adolescentis* group, and *B. bifidum* and *B. longum* complex) that were shown to fit in the deepest branch of the resulting phylogenetic tree, and position farthest to the root, suggesting the members of the *B. pseudolongum* group were divergent from the evolutionary ancestor of the genus.

We further evaluated the general genomic features and intra-species genomic diversity of these *Bifidobacterium* species (Figure 4B–G). The genome sizes varied significantly among different bifidobacterial species. We found that *B. pseudolongum* harbored the second smallest genome size following *B. animalis* (Figure 4B). There was no apparent difference in ratio of accessory genes between the various species (Figure 4F). However, *B. pseudolongum* was among the species with the highest numbers of pair-wise single nucleotide polymorphisms (SNPs) (Figure 4G).

### 3.4. COG Functions and Profiles of Carbohydrate-Utilizing Enzymes

**Figure 5:**
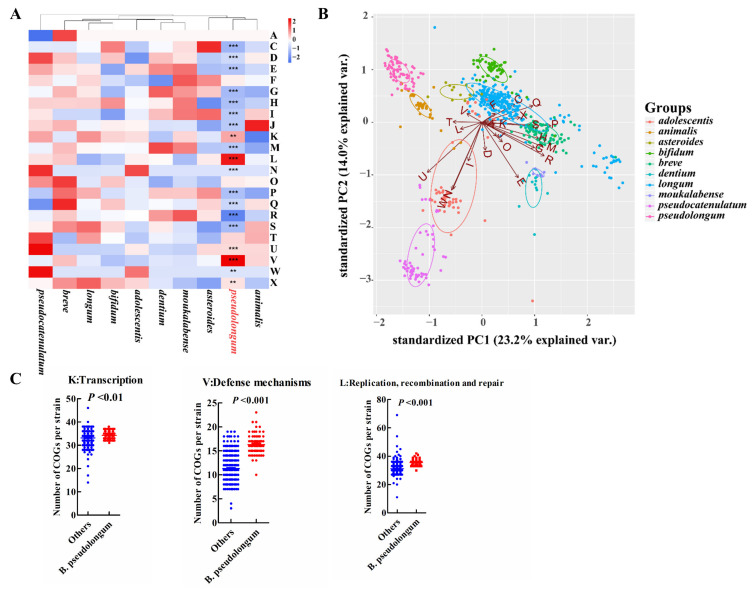
Characterization of *B. pseudolongum* in terms of COG functions among various *Bifidobacterium* species. (**A**) Heatmap of average numbers of COG function categories per strain by species. Significantly different COG functions in the comparison of *B. pseudolongum* strains and the strains of the other studied bifidobacterial species were marked. ***, *p* < 0.001; **, *p* < 0.01. A: RNA processing and modification; C: Energy production and conversion; D: Cell cycle control, cell division, chromosome partitioning; E: Amino acid transport and metabolism; F: Nucleotide transport and metabolism; G: Carbohydrate transport and metabolism; H: Coenzyme transport and metabolism; I: Lipid transport and metabolism; J: Translation, ribosomal structure and biogenesis; K: Transcription; L: Replication, recombination and repair; M: Cell wall/membrane/envelope biogenesis; N: Cell motility; O: Posttranslational modification, protein turnover, chaperones; P:Inprganic ion transport and metabolism; Q: Secondary metabolites biosynthesis, transport and catabolism; R: General function prediction only; S: Function unknown; T: Signal transduction mechanisms; U: Intracellular trafficking, secretion, and vesicular transport; V: Defense mechanisms; W: Extracellular structures; X: Mobilome: prophages, transposons. (**B**) PCA plot of COG functions by *Bifidobacterium* species. (**C**) Selected enriched COGs in *B. pseudolongum* compared with the other studied bifidobacterial species. The *Bifidobacterium* species with more than 10 publicly available sequenced genomes in the NCBI database were analyzed (including 786 strains).

**Figure 6:**
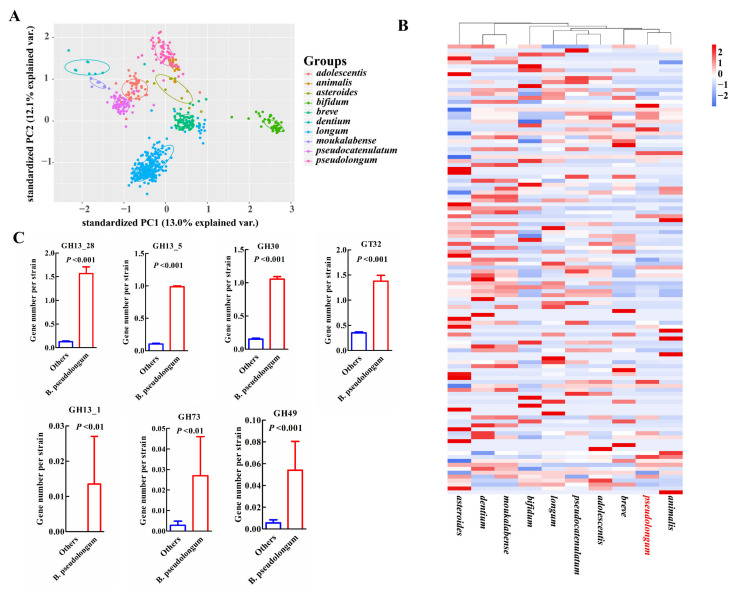
Characterization of *B. pseudolongum* in terms of carbohydrate-utilizing enzymes among various *Bifidobacterium* species. (**A**) PCA plot of GH and GT profiles of different species. (**B**) Heatmap of average numbers of GHs and GTs per strain by *Bifidobacterium* species. (**C**) Selected enriched GHs and GTs in *B. pseudolongum* compared with the other studied bifidobacterial species. GH13_1 (encompassing α-amylases), GH13_28 (encompassing α-amylases), GH13_5 (encompassing α-amylases), GH30 (representing fucosidases), GH73 (including activities of β-*N*-acetylglucosaminidases), GH49 (including activities of dextranases), and GT32 (including activities of mannosyltransferases). The *Bifidobacterium* species with more than 10 publicly available sequenced genomes in the NCBI database were analyzed (including 786 strains).

For COG categories and signatures of the carbohydrate-utilizing enzymes examined, the animal-associated species, *B. pseudolongum* and *B. animalis*, clustered together and showed more similar profiles (Figure 5A,B and Figure 6A,B). *B. pseudolongum* demonstrated significant differences on 19 of 23 COG functions in comparison to other *Bifidobacterium* species (Figure 5A). Most of these significantly different COGs were in the lower levels in *B. pseudolongum* (15/19), covering three main metabolic processes—carbohydrate transport and metabolism, amino acid transport and metabolism, and lipid transport and metabolism. In addition, *B. pseudolongum* seemed to possess lower numbers of most glycosyltransferases (GTs) and glycoside hydrolases (GHs) than other species (Figure 6B).

However, four COGs were in higher levels in *B. pseudolongum* compared with the other tested bifidobacterial species, including replication, recombination and repair (L), defense mechanisms (V), intracellular trafficking, secretion, and vesicular transport (U), and transcription (K) (Figure 5A,C). Similarly, several GHs and GTs were also enriched in *B. pseudolongum*, such as GH13_1 (encompassing α-amylases), GH13_28 (encompassing α-amylases), GH13_5 (encompassing α-amylases), GH30 (representing fucosidases), GH73 (including activities of β-*N*-acetylglucosaminidases), GH49 (including activities of dextranases), and GT32 (including activities of mannosyltransferases) (Figure 6B,C). The enzymes in the GH13 family (encompassing α-amylases) are involved in the breakdown of complex plant carbohydrates [44]. GH13_28 (63/74) and GH13_5 (73/74) distributed in most *B. pseudolongum* strains, but were not frequently found in the genomes of the other tested bifidobacterial species. GH13_1 (1/786), GH73 (including activities of β-*N*-acetylglucosaminidases; 4/786), and GH49 (including activities of dextranases; 8/786) were rare GH families within the studied *Bifidobacterium* species, and the genes encoding these enzymes were more likely to be present in *B. pseudolongum* compared with the other tested bifidobacterial species (Figure 6C). GH30, which represented fucosidases and was believed to degrade host glycans [16], was one of the core genes of *B. pseudolongum*, but showed large paucity in the other studied bifidobacterial species.

### 3.5. Probiotic Effector Molecules

To explore potential probiotic effects of *B. pseudolongum*, we mined the distribution of the current elucidated probiotic effector molecules [27,28] in *B. pseudolongum* in the background of hundreds of genomes of bifidobacterial strains.

**Figure 7:**
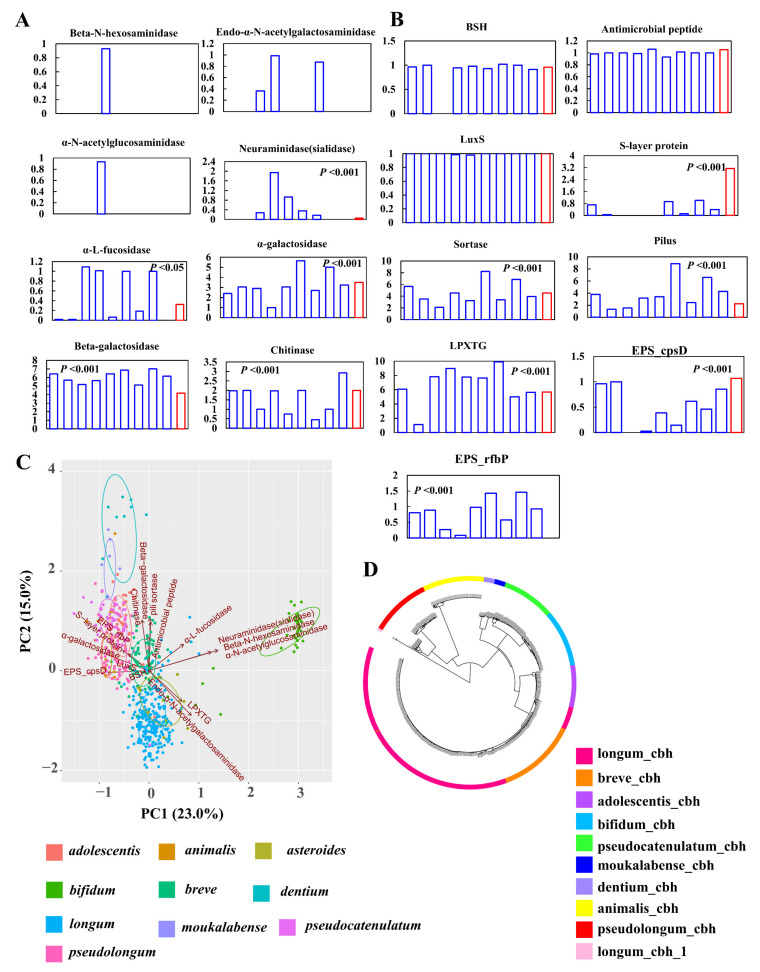
Distribution of probiotic effector molecules among *B. pseudolongum* and other *Bifidobacterium* species. (**A**) Average gene numbers of mucin-glycan foraging enzymes per strain by Bifidobacterium species. Each column represents a bifidobacterial species. From left to right: *B. adolescentis*, *B. animalis*, *B. asteroides*, *B. bifidum*, *B. breve*, *B. dentium*, *B. longum*, *B. moukalabense*, *B. pseudocatenulatum*, and *B. pseudolongum*. (**B**) Average gene numbers of probiotic effectors per strain for each species. Each column represents a bifidobacterial species. From left to right: *B. adolescentis*, *B. animalis*, *B. asteroides*, *B. bifidum*, *B. breve*, *B. dentium*, *B. longum*, *B. moukalabense*, *B. pseudocatenulatum*, and *B. pseudolongum*. (**C**) PCA plot of the strains from different *Bifidobacterium* species by taking the numbers of each included probiotic effector as inputs. (**D**) Phylotypes of BSH sequences by *Bifidobacterium* species. The BSH reference sequences were removed after evaluating the BSH types. The information for all the used BSH sequences are listed in Table S2. The *Bifidobacterium* species with more than 10 publicly available sequenced genomes in the NCBI database were analyzed (including 786 strains). Statistical analyses were performed between *B. pseudolongum* strains and the strains of the other studied bifidobacterial species.

First, we evaluated the signatures of nine key mucin-glycan foraging enzymes (Figure 7A) [41]. None of the studied *Bifidobacterium* species possessed α-*N*-acetylgalactosaminidase, and *B. bifidum* was the only member that encoded β-*N*-hexosaminidase and α-*N*-acetylglucosaminidase. Five enzymes for mucin-glycan degradation were found in the genomes of *B. pseudolongum*, and the distribution of these enzymes significantly fluctuated among different bifidobacterial species. Compared with other bifidobacteria, *B. pseudolongum* showed lower levels of neuraminidase (sialidase) and β-galactosidase, and higher levels of α-galactosidase, chitinase, and α-l-fucosidase. We found that *B. bifidum*, *B. longum*, and *B. asteroides* possessed the ability to utilize O-linked glycans (executed by Endo-α-*N*-acetylgalactosaminidase, with which bacteria could cleave glycans from mucin proteins). Although the other seven bifidobacterial species including *B. pseudolongum* did not encode this enzyme, they possessed mucin-glycan foraging potentials by cleaving side-chains of mucin-glycans (executed by one of the other seven enzymes other than Endo-α-*N*-acetylgalactosaminidase).

For other probiotic effector molecules, the mucus-binding protein and serine-rich glycoprotein adhesion could not be found in the genomes of all the tested bifidobacterial strains. A previous study has reported that complete mucus-binding domains, called MUB, were found exclusively in lactic acid bacteria [45], which supported the absence of mucus-binding proteins within the *Bifidobacterium* genus. The average gene numbers of bile salt hydrolase (BSH), antimicrobial peptide, and luxS in *B. pseudolongum* were comparable to the other tested bifidobacterial species (*p* > 0.05) (Figure 7B). *B. asteroides*, mainly found in insect guts, did not harbor BSH genes. The levels of pili-related molecules, including sortase, pilus, and LPXTG, significantly varied between *B. pseudolongum* and the other tested bifidobacterial species (*p* < 0.001). Notably, S-layer proteins were markedly enriched in *B. pseudolongum* strains. Next, we evaluated the presence and absence of exopolysaccharide (EPS) clusters in bifidobacterial genomes by searching for the priming-GTF gene (p-gtf). This gene encoded the enzyme that was in charge of the initial step of the EPS-unit biosynthesis; therefore, it should be present in all EPS clusters. We found that major bifidobacterial species showed the presence of two kinds of priming-GTF genes, while *B. pseudolongum* only harbored one priming-GTF gene (cpsD). The principal component analysis (PCA) results based on the gene numbers of all these probiotic factors also supported the fact that higher levels of EPS_rfbP and S-layer protein contributed to the separation of *B. pseudolongum* from the other tested bifidobacterial species in the PCA plot (Figure 7C).

Notably, we found that different bifidobacterial species possessed distinct types of BSH genes according to a phylogenetic tree constructed by BSH sequences (Figure 7D). After adding the reference sequences of seven types of BSH genes [43] (see Table S2 for details), we observed that the BSH genes of all the studied *Bifidobacterium* species (except that three BSH sequences from *B. longum* could not be categorized into any currently elucidated BSH types) belonged to the BSH-T4 type, and all the studied species except *B. longum* did not harbor paralogs of BSH. In addition, *B. pseudolongum* clustered with *B. animalis*, indicating they had more similar BSH sequences (Figure 7D).

### 3.6. Sub-Clade-Specific Evolution within B. pseudolongum

**Figure 8:**
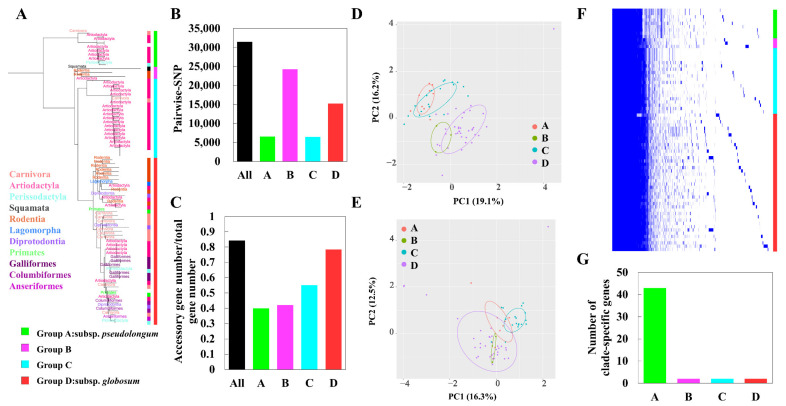
Clade-specific genomic features and intra-species evolution of *B. pseudolongum*. (**A**) Phylogenetic tree of *B. pseudolongum* constructed by the NJ method based on the bi-SNPs in the core genome of 74 strains. The tree was rooted by the clade that was nearest to the outgroup (*B. animalis* subsp. *animalis* ATCC 25527). Niche of each strain was marked on the corresponding clade and the inner strip by the taxonomic unit of “order”. Each sub-clade was indicated by color in the outer strip. (**B**,**C**) Comparison of intra-clade genomic diversity by SNP distance (**B**) and accessory genome size (**C**). (**D**) PCA plot of COG functions of *B. pseudolongum* by sub-clade. (**E**) PCA plot of GHs and GTs of *B. pseudolongum* by sub-clade. (**F**) The gene presence and absence conditions of 74 strains of *B. pseudolongum*. (**G**) Numbers of clade-specific core genes that were present in all the strains of a designated clade and absent from any strains of the other three clades. See detailed information in Table S3.

We analyzed subspecies-specific genomic features of *B. pseudolongum* to observe its evolutionary trajectory at a higher resolution (Figure 8A–G). *B. pseudolongum* represented a promiscuously host-adapted lifestyle, and could be found in diverse habitats including Mammalia, Aves, and Reptilia (Figure 8A). Four clades were shown after phylogenetic reconstruction based on core-genome bi-SNPs. Sub-clade A, corresponding with *B. pseudolongum* subsp. *pseudolongum,* showed the greatest numbers of the clade-specific genes, as represented by 43 unique genes that were present in all the strains of clade A and absent from all strains in the other three clades (Table S3 and Figure 8G). These clade-A-specific core genes covered a broad range of bacterial functions, including cell wall biosynthesis (e.g., β-hexosaminidase and LL-diaminopimelate aminotransferase), regulation (e.g., HTH-type transcriptional regulator DegA and transcriptional regulator LytR), signaling (e.g., pheromone autoinducer 2 transporter), stress response (e.g., sensor histidine kinase DesK), nucleotide metabolism (tRNA pseudouridine synthase B and DNA utilization protein GntX) and lipid metabolism (holo-[acyl-carrier-protein] synthase and dephospho-CoA kinase). The COG functions (PERMANOVA R^2^ = 0.31 and *p* < 0.001; Figure 8D) and profiles of carbohydrate-utilizing enzymes (GTs and GHs: PERMANOVA R^2^ = 0.30 and *p* < 0.001; Figure 8E) were preliminarily separated by clade. For pair-wise comparisons, COGs and carbohydrate-utilizing enzymes of bifidobacterial strains were significantly varied by clade (*p* < 0.05), except that clade B and clade D could not be distinguished with each other in terms of the profiles of GTs and GHs (*p* = 0.724).

For genomic diversity, although limited strains were included in the clade B, we observed the largest genetic distance within this cluster in terms of the pair-wise SNP numbers (Figure 8B). Clade D, corresponding to the traditional *B. pseudolongum* subsp. *globosum*, showed the largest ratio of accessory gene number/total gene number (Figure 8C). Genomic diversity in terms of the accessory gene sets seemed to be expanded across the phylogenetic tree (Figure 8C). Gene presence and absence analysis indicated the frequent appearance of the strain-specific genes for *B. pseudolongum*, which contributed to accruing accessory gene sets of the species (Figure 8F).

## 4. Discussion

The studies on evolutionary and functional genomics of various *Bifidobacterium* species have been performed for decades, yet these pieces of research largely focused on the limited number of type strains of various bifidobacterial species and mainly showed an emphasis on functional aspects instead of evolutionary roles [14,15,16,17,46]. In particular, *B. pseudolongum* seemed an overlooked species compared with those bifidobacterial species (e.g., *B. longum*) with well-documented health-promoting effects and colonization advantage [22,47]. Using population genomics analysis, we included 887 bifidobacterial genomes to investigate the evolutionary role and functional signature of *B. pseudolongum* within the bifidobacterial genus, and try to reveal the intra-species evolution and function separation. Our findings reveal important implications regarding the molecular mechanism of *Bifidobacterium* diversification.

Our results shed important light on the potential probiotic roles of *B. pseudolongum* on the host. We demonstrated that *B. pseudolongum* is more likely to colonize the gut of mice compared with rats and humans, and four bacterial taxa, including *unclassified__Clostridiaceae*, *Dorea*, *Desulfovibrio*, and *Pseudomonas* were observed to be correlated positively or negatively in abundance with *B. pseudolongum*. Notably, *Desulfovibrio* spp. are the most abundant sulfate-reducing bacteria in the mammalian gut [48]. *Desulfovibrio* spp. utilize hydrogen and other organic substrates as electron donors to reduce sulfate to hydrogen sulfide (H_2_S). The increased abundance of *Desulfovibrio* has been linked to development of inflammatory bowel disease [49] and autism spectrum disorder [50], likely caused by the accumulation of H_2_S. *Pseudomonas* spp. showed increased levels in the gut of patients with multiple sclerosis [51] and end-stage renal disease [52], represented a higher positive rate in the ileum of Crohn’s disease patients compared with the healthy control group [53], and reflected a positive correlation with pro-inflammatory cytokine IL-6 [54]. In addition, one member of the genus, *P. aeruginosa*, is a Gram-negative opportunistic bacterium that causes various infections [55]. Therefore, the negative correlation between levels of *B. pseudolongum* and levels of either *Psuedomonas* spp. (R = −0.618) or *Desulfovibrio* spp. (R = −0.582) raised the question of whether administration by *Bifidobacterium* could inhibit pathogenic *Desulfovibrio* and *Pseudomonas*. Indeed, bifidobacterial species, like *B. longum*, had been reported to be able to successfully treat ulcerative colitis with multidrug-resistant *pseudomonas aeruginosa* infection in a man [56] and prevent gut-derived *pseudomonas aeruginosa* sepsis in mice [57].

The probiotic effects of some bacterial molecules, such as mucin glycan foraging enzymes, pili, EPS, S-layer protein, bacteriocins, BSH, mucus-binding proteins, and serine-rich proteins have been revealed in various *Bifidobacterium* strains [27,28]. These bacterial molecules are known as “probiotic effector molecules” [27]. It was believed that these molecules exerted beneficial effects on host via at least five modes of action: Regulation of the composition and activity of the indigenous microbiota, improvement of epithelial barrier function, regulation of the immune system, regulation of systemic metabolic responses, and functioning via the central nervous system [27]. Here, the analysis on distribution of probiotic effector molecules in the genomes of various bifidobacterial species indicated that *B. pseudolongum* harbored mucin-glycan foraging enzymes, showed comparable numbers of BSH, antimicrobial peptide, and luxS with the other tested bifidobacterial species, but presented enriched genes encoding S-layer proteins, possible unique EPS organization, and the original BSH subtype. In particular, we observed that the major analyzed bifidobacterial species showed the presence of two kinds of priming-GTF genes, while *B. pseudolongum* only harbored one priming-GTF gene (cpsD). A previous study based on the analysis of 28 complete genomes of bifidobacterial strains reported that functional-structural organization of bifido-EPS was of large dissimilarity, and structures and lengths of the clusters with one priming-GTF gene were very different from those with two priming-GTF genes [42]. These results could provide a valuable knowledge basis for exploring similarity and uniqueness with respect to probiotic effects on the host between *B. pseudolongum* and each of the other tested bifidobacterial species that are with well-studied health-promoting phenotypes and related mechanistic insights.

This is the first-known example to reveal species-level population structures of various bifidobacterial species and give insight into their genomic diversity based on genome information. Our results indicated that those animal-associated species, including *B. pseudolongum* (Fst = 0.70) and *B. animalis* (Fst = 0.85), appeared to form multi-clade population structures, whereas some of human-associated bifidobacterial (sub)species showed radiating structures with limited population differentiation. Correspondingly, a multi-clade phylogenetic shape has been reported for another champion colonizer in the gut of various animals, *L. reuteri* [55]. For *L. reuteri*, it was considered that distinct monophyletic clades represented host origins but not geographical locations, which was regarded to be consequences of long-term colonization by *L. reuteri* lineages in the gut of specific vertebrate species and host-driven diversification [58,59]. Similarly, we also observed host-specific lineages for *B. pseudolongum* and *B. animalis*, indicating host-driven genomic diversification. For the intra-genus evolutionary role of *B. pseudolongum*, consistent with previous studies [15,17], six main groups were categorized, including the *B. pseudolongum* group, *B. adolescentis* group, *B. pullorum* group, *B. boum* group, *B. bifidum* and *B. longum* complex, and *B. asteroides* group. We observed that the *B. asteroides* phylogenetic group was shown at the position closest to the root of the tree, while *B. pseudolongum* belonged to one of the three groups (the *B. pseudolongum* group, *B. adolescentis* group, and *B. bifidum* and *B. longum* complex) that were shown to fit in the deepest branch of the resulting phylogenetic tree, suggesting the members of the *B. pseudolongum* group were divergent from the evolutionary ancestor of the genus. A previous study has reported that in a family-based supertree of the *Bifidobacteriaceae*, bifidobacteria were demonstrated to locate in the deepest branch of the phylogenetic tree, and separated them from other genera within this family. Meanwhile, in the same study, the *B. asteroides* phylogenetic group was shown to position closest to the root in this family-based supertree, therefore indicating that members of this group most closely resemble the evolutionary ancestor of the *Bifidobacterium* genus. The observed substantial genomic size difference among various bifidobacterial species was previously considered as a reminiscent evidence of an evolutionary route that has involved a lot of gene loss and/or gain events [16]. It was previously reported that host-adapted species tend to harbor a reduced genome size during the lifestyle transition from free-living to host-adapted when the deletion of redundant genetic content occurs [60]. Here, we found that *B. pseudolongum* harbored the second smallest genome size, which could possibly explain its better host adaptability. In addition, we found that *B. pseudolongum* showed the highest number of pair-wise SNPs, reflecting its higher intra-species genetic diversity [61].

The present work implies considerable functional divergence of *B. pseudolongum* from the other included bifidobacterial species in terms of COG terms and carbohydrate-utilizing enzymes. We found that *B. pseudolongum* demonstrated a significant difference on 19 of 23 COG functions in comparison to other *Bifidobacterium* species, and most of these significantly different COGs were in lower levels in *B. pseudolongum* (15/19). The analysis on carbohydrate-utilizing enzymes indicated that *B. pseudolongum* seemed to possess lower numbers of the most of GTs and GHs than other species. It has been reported that bacteria tend to shut down carbohydrate transportation and reduce energy metabolism in response to environmental stresses [62,63,64], and even discard some redundant genes encoding carbohydrate-utilizing enzymes in order to reduce energy consumption and improve niche fitness [65,66]. In contrast, several GHs and GTs involved in the breakdown of complex plant carbohydrates and host glycans were enriched in *B. pseudolongum*. Very limited studies have focused on carbohydrate metabolic ability of *B. pseudolongum.* Studies have reported that *B. pseudolongum* could use starch as a sole source of carbon and energy, possibly due to its α-glucosidase activity [12,67]. Additionally, *B. pseudolongum* encoded pectin-degrading enzymes and could degrade pectin [68].

The study on intra-species evolution of *B. pseudolongum* here highlights taxonomic inconsistencies and reveals novel clade-associated features. We demonstrated that four clades were shown after phylogenetic reconstruction based on core-genome bi-SNPs, despite the current recognition of only two subspecies—*B. pseudolongum* subsp. *pseudolongum* and *B. pseudolongum* subsp. *globosum* [12]. In addition, considerable clade-specific genes, functional separation by clade, and expansion of genomic diversity along the phylogenetic tree were observed. In a previous study, 47 bifidobacterial (sub)species have been clustered into three distinct groups based on the profiles of GHs (GHP/A, GHP/B, and GHP/C) [69]. In this context, two subspecies of *B. thermacidophilum* were clustered into GHP/B, and two subspecies of *B. animalis* were clustered into GHP/A. However, *B. pseudolongum* subsp. *pseudolongum* and *B. pseudolongum* subsp. *globosum* were classified into two separate groups—GHP/A and GHP/B, respectively. GHP/A cluster showed an extensive array of putative GH43 family members (involved in the degradation of complex plant glycans), suggesting adaptation of bifidobacterial (sub)species within the group GHP/A to hosts that adopted a vegetarian or omnivorous lifestyle [16,69]. Members of the GHP/B cluster were featured by the paucity of GH43 and GH3 enzymes. Therefore, the separation of GH and GT repertoires according to the clades of *B. pseudolongum* observed here and in the above-mentioned previous study suggested possible intra-species metabolic diversity.

## 5. Limitations

Our study has several limitations. For the analysis of colonized niches for *B. pseudolongum*, we only selected fecal samples from rodents (Balb/c mice, C57/B6 mice, and Wistar rats) and humans that were easily available and collected. Although the results demonstrated distribution differences of *B. pseudolongum* and the suggested preference of *B. pseudolongum* in the gut of Balb/c mice, additional insights could be reached when fecal samples from more types of animals (such as ungulates, carnivores, chicken, and pigeons) are included. In addition, for the analysis of sub-clade-specific evolution within *B. pseudolongum*, the sub-clade-specific genes were analyzed by Roary software. This software is based on protein sequences, and thus might introduce a minute quantity of false-positive and false-negative hits. If further studies are focused on one or several of these specific genes, it is necessary to validate the present/absent status of them via BLASTN and/or PCR experiments. Furthermore, most parts of this study were based on genomic analysis. Some interesting findings here, such as possible different activity of BSH sub-types, and the structural and functional organizations of the EPS clusters of *B. pseudolongum* and other bifidobacterial species, need further investigations both in vitro and in vivo. Finally, we only gave details of and analyzed the bifidobacterial species with more than 10 publicly available genomes. With increased access to more genomes of other bifidobacterial species that were not analyzed here, more information could be obtained, and we could further contribute to the understanding of genomic diversity and evolution of the genus *Bifidobacterium*.

## 6. Conclusions

Taken together, *B. pseudolongum* showed a multi-clade population structure, and established in various mammalian animals a rather strict association with humans. Its abundance was negatively correlated with two harmful gut bacterial taxa (*Psuedomonas* sp. and *Desulfovibrio* sp.). Within the *Bifidobacterium* genus, *B. pseudolongum* represented a relatively smaller genome size and higher intra-species genetic diversity. The paucity of metabolism-related functions and enrichment of specific enzymes degrading complex plant carbohydrates and host glycans might provide a possible explanation for its prevalence and dominance across the mammalian branch of the tree of life. *B. pseudolongum* had a higher level of S-layer proteins, unique BSH subtype, and possible particular EPS cluster organization. Finally, considerable sub-clade specific genes, separation of profiles of carbohydrate-utilizing enzymes and COGs by clade, and variations on genomic diversity across the phylogenetic tree were also observed.

## Data Availability

The metagenomic sequencing data have been deposited into sequence read archive (SRA) database under accession number PRJNA576558.

## Supplementary Materials

The following are available online at https://www.mdpi.com/article/10.3390/foods10102284/s1. Figure S1. Numbers of publicly available genomes for each bifidobacterial species (A), and maximum likelihood (ML) of them (B). Numbers of publicly available sequenced genomes for each *Bifidobacterium* species. Red, more than 50 genome assemblies; light orange, more than 10; light pink, more than 5; green, more than 1. Species in the right section behind the perpendicular line were without sequenced genomes in the NCBI database. Figure S2. Niche distribution along the phylogenetic tree (NJ tree) for *B. pseudolongum* (A) and *B. animalis* (B). Figure S3. Phylogenetic tree (NJ tree) of genus *Bifidobacterium* with the bootstrap values of the clades indicated. Bootstrap of 100 was set for tree construction. Table S1. The information on genome assemblies used in the study. Table S2. The information of sequences used in phylogenetic reconstruction of BSH genes. Table S3. The clade-specific core genes of *B. pseudolongum.*

## Abbreviations

DGGE: Denaturing gradient gel electrophoresis; ITS: Internally transcribed spacer; COG: Clusters of orthologous groups of protein; GHs: Glycoside hydrolases; GTs: Glycosyltransferases; BSH: Bile salt hydrolase; EPS: Exopolysaccharide; GC: Guanine-Cytosine; CD: Coding sequence; SNP: Single nucleotide polymorphisms; NJ: Neighbor-joining; ML: Maximum-likelihood; PCA: Principal component analysis.
