# Supplementary material for: Colonized Niche, Evolution and Function Signatures of Bifidobacterium pseudolongum within Bifidobacterial Genus
Source: Foods. 2021 Sep 27;10(10):2284. doi: 10.3390/foods10102284 (PMC8535030; doi:10.3390/foods10102284)
Supplement: Supplementary file 1 [file foods-10-02284-s001.zip › foods-1357924-supplementary.pdf]

**Supplementary materials for Foods**

# Colonized niche, evolution and function signatures of *Bifidobacterium pseudolongum* within bifidobacterial genus

Yue Xiao[a,b], Jianxin Zhao[a,b], Hao Zhang[a,b,c,d,e], Qixiao Zhai[a,b,f*],Wei Chen [a, b, c]

[a] State Key Laboratory of Food Science and Technology, Jiangnan University, Wuxi, Jiangsu 214122, People's Republic of China

[b] School of Food Science and Technology, Jiangnan University, Wuxi, Jiangsu 214122, China

[c] National Engineering Research Center for Functional Food, Jiangnan University, Wuxi, Jiangsu 214122, China

[d] (Yangzhou) Institute of Food Biotechnology, Jiangnan University, Yangzhou 225004, China

[e]Wuxi Translational Medicine Research Center and Jiangsu Translational Medicine Research Institute Wuxi Branch

[f] International Joint Research Laboratory for Probiotics at Jiangnan University, Wuxi, Jiangsu 214122, China

**Running title:** Population genomics of *Bifidobacterium pseudolongum*

[*] Corresponding author: Qixiao Zhai.

Tel: 86-510-85912155; Fax: 86-510-85912155

E-mail address: zhaiqixiao@sina.com

Postal address: Department of Food Science and Technology, Jiangnan University, Lihu Road No.1800, Binhu District, Wuxi, Jiangsu, 214122 P. R. China

## Figure legends

**Figure S1 Numbers of publicly available genomes for each bifidobacterial species (A), and maximum likelihood (ML) of them (B).** Numbers of publicly available sequenced genomes for each *Bifidobacterium* species. Red, more than 50 genome assemblies; light orange, more than 10; light pink, more than 5; green, more than 1. Species in the right section behind the perpendicular line were without sequenced genomes in the NCBI database.

**Figure S2 Niche distribution along the phylogenetic tree (NJ tree) for *B. pseudolongum* (A) and *B. animalis* (B).**

**Figure S3 Phylogenetic tree (NJ tree) of genus *Bifidobacterium* with the bootstrap values of the clades indicated.** Bootstrap of 100 was set for tree construction.

Figure S1

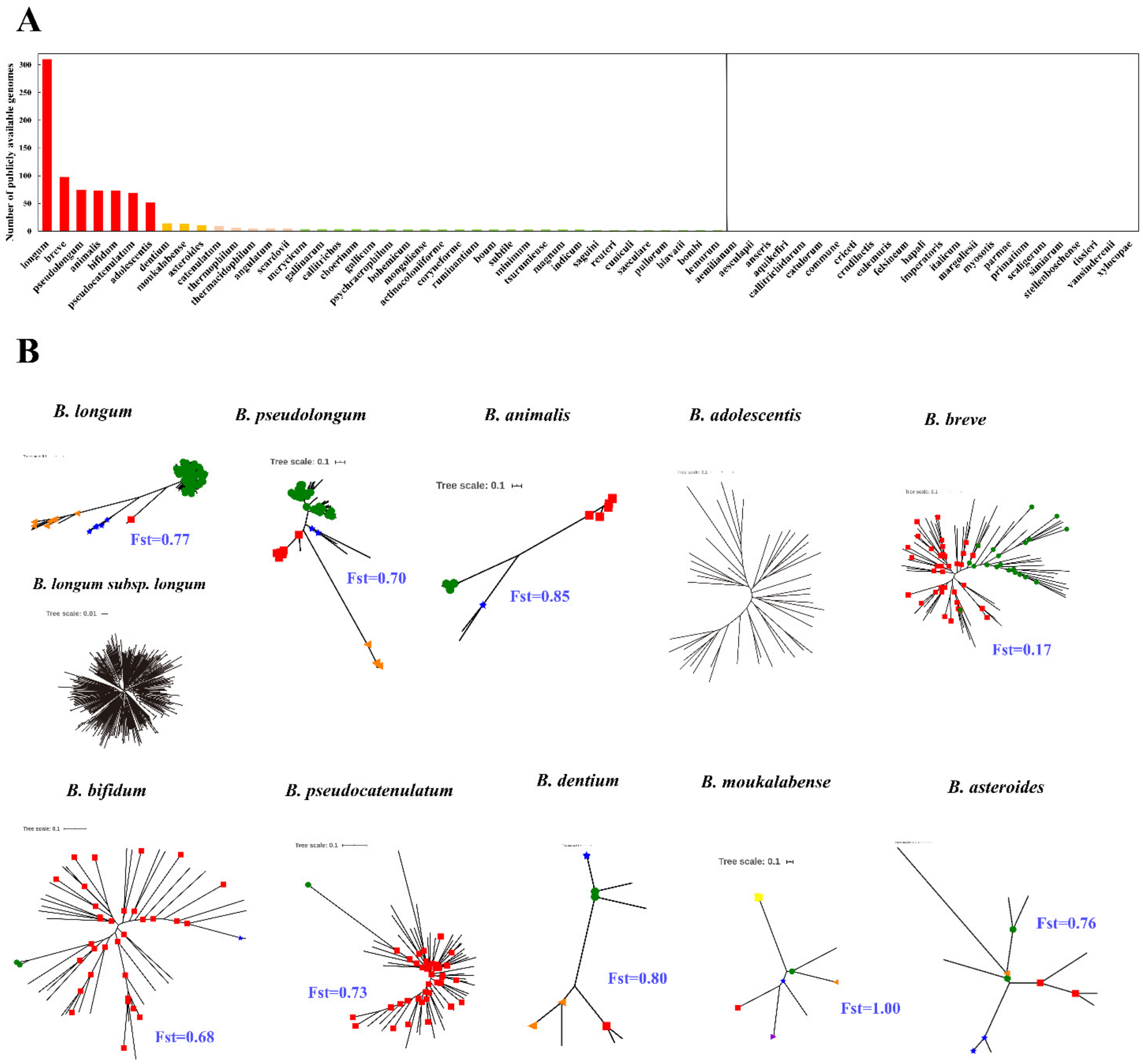
A
Number of publicly available genomes
0
50
100
150
200
250
300
longum
breve
pseudolongum
animalis
bifidum
pseudocatenulatum
adolescentis
dentium
moukalabense
asteroides
catenulatum
thermophilum
thermacidophilum
angulatum
scardovii
merycicum
gallinarum
callitrichos
choerinum
gallicum
psychraerophilum
bohemicum
mongoliense
actinocoloniiforme
coryneforme
ruminantium
boum
subtile
minimum
tsurumiense
magnum
indicum
saguini
reuteri
cuniculi
saeculare
pullorum
biavatii
bombi
lemurum
aemilianum
aesculapii
anseris
aquikefiri
callitrichidarum
catulorum
commune
criceti
crudilactis
eulemuris
felsineum
hapali
imperatoris
italicum
margollesii
myosotis
parmae
primatium
scaligerum
simiarum
stellenboschense
tissieri
vansinderenii
xylocopae
B
B. longum
Fst=0.77
B. longum subsp. longum
Tree scale: 0.01
B. pseudolongum
Tree scale: 0.1
Fst=0.70
B. animalis
Tree scale: 0.1
Fst=0.85
B. adolescentis
B. breve
Fst=0.17
B. bifidum
Tree scale: 0.1
Fst=0.68
B. pseudocatenulatum
Fst=0.73
B. dentium
Fst=0.80
B. moukalabense
Tree scale: 0.1
Fst=1.00
B. asteroides
Fst=0.76

Figure S2

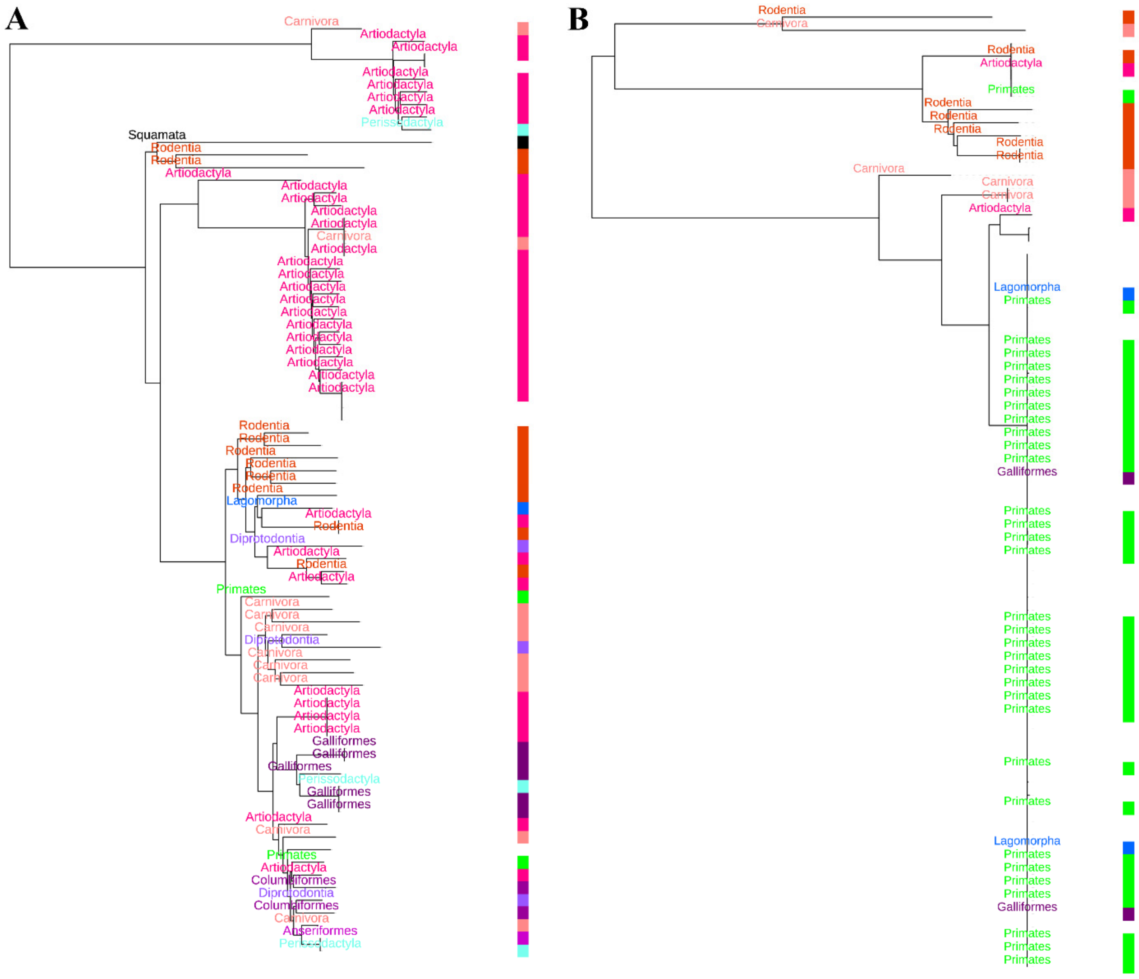
A
Carnivora
Artiodactyla
Artiodactyla
Artiodactyla
Artiodactyla
Artiodactyla
Artiodactyla
Perissodactyla
Squamata
Rodentia
Rodentia
Artiodactyla
Artiodactyla
Artiodactyla
Artiodactyla
Artiodactyla
Carnivora
Artiodactyla
Artiodactyla
Artiodactyla
Artiodactyla
Artiodactyla
Artiodactyla
Artiodactyla
Artiodactyla
Artiodactyla
Artiodactyla
Artiodactyla
Artiodactyla
Rodentia
Rodentia
Rodentia
Rodentia
Rodentia
Rodentia
Lagomorpha
Artiodactyla
Rodentia
Diprotodontia
Artiodactyla
Rodentia
Artiodactyla
Primates
Carnivora
Carnivora
Carnivora
Diprotodontia
Carnivora
Carnivora
Carnivora
Artiodactyla
Artiodactyla
Artiodactyla
Artiodactyla
Galliformes
Galliformes
Galliformes
Perissodactyla
Galliformes
Galliformes
Artiodactyla
Carnivora
Primates
Artiodactyla
Columbiformes
Diprotodontia
Columbiformes
Carnivora
Anseriformes
Perissodactyla
B
Rodentia
Carnivora
Rodentia
Artiodactyla
Primates
Rodentia
Rodentia
Rodentia
Rodentia
Rodentia
Carnivora
Carnivora
Carnivora
Artiodactyla
Lagomorpha
Primates
Primates
Primates
Primates
Primates
Primates
Primates
Primates
Primates
Primates
Primates
Galliformes
Primates
Primates
Primates
Primates
Primates
Primates
Primates
Primates
Primates
Primates
Primates
Primates
Primates
Primates
Lagomorpha
Primates
Primates
Primates
Primates
Galliformes
Primates
Primates
Primates

Figure S3

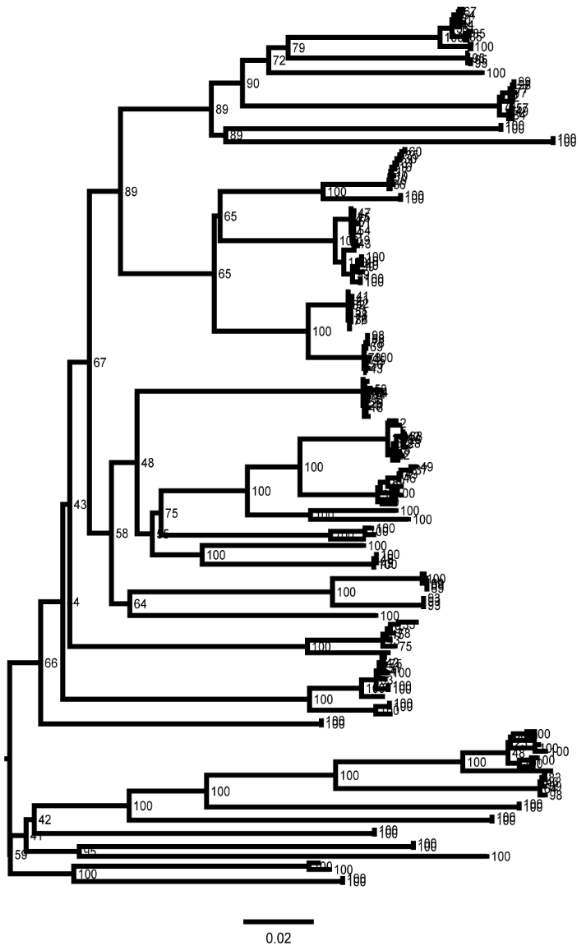

## Table S1 The information on genome assemblies used in the study

| Organism/Name | Strain | BioSample | Size (Mb) | GC% | Proteins | Genome ID |
|---|---|---|---|---|---|---|
| *Bifidobacterium actinocoloniiforme* DSM 22766 | DSM 22766 | SAMN02673422 | 1.82339 | 62.7 | 1417 | GCF_000741095.1_Bifact_genomic.fna |
| *Bifidobacterium actinocoloniiforme* DSM 22766 | DSM 22766 | SAMN02442035 | 1.81848 | 62.7 | 1407 | GCF_000771585.1_DSM-22766_genomic.fna |
| *Bifidobacterium actinocoloniiforme* DSM 22766 | DSM 22766 | SAMN02442035 | 1.83006 | 62.7 | 1422 | GCF_001263395.1_ASM126339v1_genomic.fna |
| *Bifidobacterium adolescentis* | AF45-19 | SAMN09734719 | 2.24738 | 59.5 | 1432 | GCA_003473345.1_ASM347334v1_genomic.fna |
| *Bifidobacterium adolescentis* | UBA2084 | SAMN06457481 | 1.54188 | 58.6 | - | GCA_002331865.1_ASM233186v1_genomic.fna |
| *Bifidobacterium adolescentis* | 22L | SAMN02673695 | 2.20322 | 59.3 | 1713 | GCF_000737885.1_ASM73788v1_genomic.fna |
| *Bifidobacterium adolescentis* | BBMN23 | SAMN03273368 | 2.17372 | 59.3 | 1696 | GCF_000817995.1_ASM81799v1_genomic.fna |
| *Bifidobacterium adolescentis* | IVS-1 | SAMN03076346 | 2.26346 | 59.6 | 1629 | GCF_000829865.1_ASM82986v1_genomic.fna |
| *Bifidobacterium adolescentis* | 150 | SAMN03468113 | 2.31566 | 59.4 | 1844 | GCF_001010915.1_ASM101091v1_genomic.fna |
| *Bifidobacterium adolescentis* | 2789STDY5834850 | SAMEA3545316 | 2.23413 | 59.4 | 1749 | GCF_001406215.1_13470_2_69_genomic.fna |
| *Bifidobacterium adolescentis* | 2789STDY5608824 | SAMEA3545233 | 2.31205 | 59.4 | 1862 | GCF_001406455.1_13414_6_8_genomic.fna |
| *Bifidobacterium adolescentis* | 2789STDY5608862 | SAMEA3545270 | 2.25908 | 59.3 | 1743 | GCF_001406735.1_13414_6_46_genomic.fna |
| *Bifidobacterium adolescentis* | Km 4 | SAMN05300513 | 2.42497 | 59.8 | 1931 | GCF_001756865.1_ASM175686v1_genomic.fna |
| *Bifidobacterium adolescentis* | 1892B | SAMN06621712 | 2.15085 | 59.4 | 1678 | GCF_002075965.1_Bbif1892B_genomic.fna |
| *Bifidobacterium adolescentis* | 42B | SAMN04231308 | 2.21274 | 59.2 | 1752 | GCF_002107925.1_Bado42Bv1_genomic.fna |
| *Bifidobacterium adolescentis* | 70B | SAMN04231309 | 2.21213 | 58.8 | 1712 | GCF_002107955.1_Bado70Bv1_genomic.fna |
| *Bifidobacterium adolescentis* | AL12-4 | SAMN04231313 | 2.09491 | 59.4 | 1632 | GCF_002107975.1_BadoAL124v1_genomic.fna |
| *Bifidobacterium adolescentis* | LMG 10734 | SAMN04231317 | 2.14863 | 59.2 | 1621 | GCF_002107995.1_BadoLMG10734v1_genomic.fna |
| *Bifidobacterium adolescentis* | 487B | SAMN04231310 | 2.59778 | 59.1 | 2139 | GCF_002108015.1_Bado487Bv1_genomic.fna |
| *Bifidobacterium adolescentis* | 703B | SAMN04231311 | 2.37281 | 59.3 | 1932 | GCF_002108035.1_Bado703Bv1_genomic.fna |
| *Bifidobacterium adolescentis* | AD2-8 | SAMN04231312 | 2.37972 | 59.9 | 1920 | GCF_002108045.1_BadoAD28v1_genomic.fna |
| *Bifidobacterium adolescentis* | AL46-2 | SAMN04231314 | 2.20856 | 59.5 | 1697 | GCF_002108075.1_BadoAL462v1_genomic.fna |
| *Bifidobacterium adolescentis* | AL46-7 | SAMN04231315 | 2.20303 | 59.1 | 1736 | GCF_002108095.1_BadoAD467v1_genomic.fna |

| | | | | | | |
|---|---|---|---|---|---|---|
| *Bifidobacterium adolescentis* | LMG 10733 | SAMN04231316 | 2.08423 | 59.2 | 1599 | GCF_002108135.1_BadoLMG10733v1_genomic.fna |
| *Bifidobacterium adolescentis* | LMG 11579 | SAMN04231318 | 2.06169 | 59.4 | 1562 | GCF_002108155.1_BadoLMG11579v1_genomic.fna |
| *Bifidobacterium adolescentis* | LMG 18897 | SAMN04231319 | 2.14679 | 59.8 | 1681 | GCF_002108165.1_BadoLMG188971_genomic.fna |
| *Bifidobacterium adolescentis* | 43476 | SAMN08770276 | 2.19243 | 59.6 | 1747 | GCF_003030905.1_ASM303090v1_genomic.fna |
| *Bifidobacterium adolescentis* | 6 | SAMN07551488 | 2.20181 | 59.3 | 1716 | GCF_003429385.1_ASM342938v1_genomic.fna |
| *Bifidobacterium adolescentis* | TM06-4 | SAMN09736994 | 2.15038 | 59.1 | 1672 | GCF_003436185.1_ASM343618v1_genomic.fna |
| *Bifidobacterium adolescentis* | TF06-2AC | SAMN09736894 | 2.26572 | 59.6 | 1828 | GCF_003437735.1_ASM343773v1_genomic.fna |
| *Bifidobacterium adolescentis* | TF06-29 | SAMN09736893 | 2.16352 | 59.2 | 1731 | GCF_003437755.1_ASM343775v1_genomic.fna |
| *Bifidobacterium adolescentis* | TF06-10AC | SAMN09736887 | 2.16249 | 59.2 | 1725 | GCF_003437775.1_ASM343777v1_genomic.fna |
| *Bifidobacterium adolescentis* | AF28-4AC | SAMN09734547 | 2.14198 | 59.2 | 1680 | GCF_003457765.1_ASM345776v1_genomic.fna |
| *Bifidobacterium adolescentis* | AF21-27 | SAMN09734426 | 2.27784 | 59.4 | 1819 | GCF_003458805.1_ASM345880v1_genomic.fna |
| *Bifidobacterium adolescentis* | OF04-5 | SAMN09736710 | 2.14192 | 59.2 | 1677 | GCF_003462885.1_ASM346288v1_genomic.fna |
| *Bifidobacterium adolescentis* | OF04-9AC | SAMN09736711 | 2.14218 | 59.2 | 1680 | GCF_003462895.1_ASM346289v1_genomic.fna |
| *Bifidobacterium adolescentis* | AF15-3 | SAMN09734284 | 2.19712 | 59.4 | 1721 | GCF_003464325.1_ASM346432v1_genomic.fna |
| *Bifidobacterium adolescentis* | AF14-56 | SAMN09734269 | 2.25699 | 59.5 | 1800 | GCF_003465205.1_ASM346520v1_genomic.fna |
| *Bifidobacterium adolescentis* | TM06-51 | SAMN09736997 | 2.22524 | 59.2 | 1758 | GCF_003466335.1_ASM346633v1_genomic.fna |
| *Bifidobacterium adolescentis* | AM41-17 | SAMN09736531 | 2.09457 | 59.2 | 1625 | GCF_003467335.1_ASM346733v1_genomic.fna |
| *Bifidobacterium adolescentis* | AM36-3AC | SAMN09736483 | 2.21547 | 59.2 | 1727 | GCF_003468385.1_ASM346838v1_genomic.fna |
| *Bifidobacterium adolescentis* | AM34-11 | SAMN09736459 | 2.17339 | 59.3 | 1686 | GCF_003469145.1_ASM346914v1_genomic.fna |
| *Bifidobacterium adolescentis* | AM14-37 | SAMN09734811 | 2.19316 | 59.1 | 1721 | GCF_003472095.1_ASM347209v1_genomic.fna |
| *Bifidobacterium adolescentis* | AM12-59 | SAMN09734791 | 2.28883 | 59.2 | 1779 | GCF_003472245.1_ASM347224v1_genomic.fna |
| *Bifidobacterium adolescentis* | AM12-20 | SAMN09734786 | 2.29387 | 59.3 | 1794 | GCF_003472265.1_ASM347226v1_genomic.fna |
| *Bifidobacterium adolescentis* | AM13-11 | SAMN09734794 | 2.26324 | 59.4 | 1847 | GCF_003473105.1_ASM347310v1_genomic.fna |
| *Bifidobacterium adolescentis* | P2P3 | SAMN08093360 | 2.20298 | 59.4 | 1718 | GCF_003856735.1_ASM385673v1_genomic.fna |
| *Bifidobacterium adolescentis* | ca_0067 | SAMN10239583 | 2.1492 | 59.5 | 1684 | GCF_004167585.1_ASM416758v1_genomic.fna |
| *Bifidobacterium adolescentis* | 1001271st1_A4 | SAMN10863259 | 2.25153 | 59.3 | - | GCF_005845205.1_ASM584520v1_genomic.fna |
| *Bifidobacterium adolescentis* | NCTC11814 | SAMEA4362428 | 2.20499 | 59.2 | 1661 | GCF_900445615.1_49964_F01_genomic.fna |
| ***Bifidobacterium adolescentis* ATCC 15703** | **ATCC 15703** | **SAMD00061080** | **2.08964** | **59.2** | **1624** | **GCF_000010425.1_ASM1042v1_genomic.fna** |

| | | | | | | |
|---|---|---|---|---|---|---|
| *Bifidobacterium adolescentis* DSM 20087 | DSM 20087 | SAMN02841222 | 2.05115 | 59.4 | 1586 | GCF_000702865.1_ASM70286v1_genomic.fna |
| *Bifidobacterium adolescentis* L2-32 | L2-32 | SAMN00627056 | 2.38911 | 59.3 | 1906 | GCF_000154085.1_ASM15408v1_genomic.fna |
| *Bifidobacterium angulatum* | LMG 11039 | SAMN02673423 | 2.00381 | 59.4 | 1498 | GCF_000741065.1_Bifang_genomic.fna |
| *Bifidobacterium angulatum* | GT102 | SAMN03435124 | 2.06464 | 59.3 | 1574 | GCF_000966445.2_ASM96644v2_genomic.fna |
| *Bifidobacterium angulatum* DSM 20098 = JCM 7096 | DSM 20098 | SAMN00008777 | 2.00821 | 59.4 | 1526 | GCF_000156635.1_ASM15663v1_genomic.fna |
| *Bifidobacterium angulatum* DSM 20098 = JCM 7096 | DSM 20098 | SAMN02442016 | 1.99378 | 59.4 | 1516 | GCF_000771205.1_DSM-20098_genomic.fna |
| *Bifidobacterium angulatum* DSM 20098 = JCM 7096 | JCM 7096 | SAMD00061039 | 2.02197 | 59.4 | 1520 | GCF_001025155.1_ASM102515v1_genomic.fna |
| *Bifidobacterium animalis* | RH | SAMN02797738 | 1.93106 | 60.5 | 1576 | GCF_000695895.1_ASM69589v1_genomic.fna |
| *Bifidobacterium animalis* | A6 | SAMN03273367 | 1.95865 | 60.5 | 1584 | GCF_000817045.1_ASM81704v1_genomic.fna |
| *Bifidobacterium animalis* | BL3 | SAMN05631165 | 1.94432 | 60.5 | 1572 | GCF_002220485.1_ASM222048v1_genomic.fna |
| *Bifidobacterium animalis* | DS15_2 | SAMN06464093 | 1.91929 | 60.5 | 1570 | GCF_003094875.1_ASM309487v1_genomic.fna |
| *Bifidobacterium animalis* | DS11_2 | SAMN06464092 | 1.9205 | 60.5 | 1574 | GCF_003094895.1_ASM309489v1_genomic.fna |
| *Bifidobacterium animalis* | DS20_2 | SAMN06464095 | 1.9289 | 60.4 | 1584 | GCF_003095055.1_ASM309505v1_genomic.fna |
| *Bifidobacterium animalis* | DS2_2 | SAMN06464094 | 1.91686 | 60.5 | 1567 | GCF_003095075.1_ASM309507v1_genomic.fna |
| *Bifidobacterium animalis* | DS1_2 | SAMN06464091 | 1.92058 | 60.5 | 1576 | GCF_003095115.1_ASM309511v1_genomic.fna |
| *Bifidobacterium animalis* | TF04-14 | SAMN09736869 | 1.91757 | 60.5 | 1573 | GCF_003438565.1_ASM343856v1_genomic.fna |
| *Bifidobacterium animalis* | OM05-7AA | SAMN09736809 | 1.91678 | 60.5 | 1567 | GCF_003438945.1_ASM343894v1_genomic.fna |
| *Bifidobacterium animalis* | 1 | SAMN10817706 | 1.93163 | 60.5 | 1569 | GCF_004135895.1_ASM413589v1_genomic.fna |
| *Bifidobacterium animalis* | co_0103 | SAMN10239584 | 1.92019 | 60.4 | 1574 | GCF_004167905.1_ASM416790v1_genomic.fna |
| *Bifidobacterium animalis* | BSD2780061688st1_E5 | SAMN10863267 | 1.9192 | 60.4 | - | GCF_005844245.1_ASM584424v1_genomic.fna |
| *Bifidobacterium animalis* | Bifido_08 | SAMEA51820918 | 1.95436 | 60.4 | 1583 | GCF_900157045.1_Bifido_08_v1_genomic.fna |
| *Bifidobacterium animalis* | Bifido_11 | SAMEA51823168 | 1.93843 | 60.4 | 1582 | GCF_900157135.1_Bifido_11_v1_genomic.fna |
| *Bifidobacterium animalis* subsp. *animalis* | 2022B | SAMN10459546 | 2.40519 | 60.1 | 1933 | GCA_004154635.1_ASM415463v1_genomic.fna |
| *Bifidobacterium animalis* subsp. *animalis* | LMG 10508 | SAMN02673424 | 1.91501 | 60.5 | 1454 | GCF_000741485.1_Bifani_sub.ani_genomic.fna |

| | | | | | | |
|---|---|---|---|---|---|---|
| *Bifidobacterium animalis* subsp. *animalis* | YL2 | SAMN04621611 | 2.02737 | 60.2 | 1566 | GCF_001688645.2_ASM168864v2_genomic.fna |
| *Bifidobacterium animalis* subsp. *animalis* | YL2 | SAMN03854118 | 2.02193 | 60.2 | 1565 | GCF_002221565.2_ASM222156v2_genomic.fna |
| *Bifidobacterium animalis* subsp. *animalis* | CNCM I-4602 | SAMN08774047 | 1.93269 | 60.5 | 1486 | GCF_003671995.1_ASM367199v1_genomic.fna |
| *Bifidobacterium animalis* subsp. *animalis* | 2006B | SAMN10459547 | 2.16359 | 60.3 | 1685 | GCF_004154625.1_ASM415462v1_genomic.fna |
| *Bifidobacterium animalis* subsp. *animalis* ATCC 25527 | ATCC 25527 | SAMN02603717 | 1.93269 | 60.5 | 1483 | GCF_000260715.1_ASM26071v1_genomic.fna |
| *Bifidobacterium animalis* subsp. *animalis* ATCC 27672 | ATCC 27672 | SAMN03978812 | 1.99016 | 60.1 | 1530 | GCF_001264055.1_ASM126405v1_genomic.fna |
| *Bifidobacterium animalis* subsp. *animalis* IM386 | IM386 | SAMEA3158463 | 1.92569 | 60.5 | 1471 | GCF_001039715.1_BAN2_genomic.fna |
| *Bifidobacterium animalis* subsp. *animalis* MCC 0483 | MCC 0483 | SAMN03978806 | 2.17604 | 60.1 | 1750 | GCF_001263945.1_ASM126394v1_genomic.fna |
| *Bifidobacterium animalis* subsp. *animalis* MCC 0499 | MCC 0499 | SAMN03978809 | 2.13378 | 60.2 | 1721 | GCF_001263865.1_ASM126386v1_genomic.fna |
| *Bifidobacterium animalis* subsp. *animalis* MCC 1489 | MCC 1489 | SAMN03978810 | 1.90992 | 60.5 | 1484 | GCF_001263835.1_ASM126383v1_genomic.fna |
| *Bifidobacterium animalis* subsp. *lactis* | BF052 | SAMN02941905 | 1.93862 | 60.5 | 1576 | GCF_000818055.1_ASM81805v1_genomic.fna |
| *Bifidobacterium animalis* subsp. *lactis* | 646 | SAMN05916063 | 1.92329 | 60.5 | 1533 | GCF_001892925.1_ASM189292v1_genomic.fna |
| *Bifidobacterium animalis* subsp. *lactis* | BM 25 | SAMN08107561 | 1.91906 | 60.5 | 1446 | GCF_002803775.1_ASM280377v1_genomic.fna |
| *Bifidobacterium animalis* subsp. *lactis* | LMG P-17502_1 | SAMN07187788 | 1.91729 | 60.5 | 1573 | GCF_002914815.1_ASM291481v1_genomic.fna |
| *Bifidobacterium animalis* subsp. *lactis* | LMG P-17502_2 | SAMN07187789 | 1.91649 | 60.5 | 1571 | GCF_002914895.1_ASM291489v1_genomic.fna |

| | | | | | | |
|---|---|---|---|---|---|---|
| *Bifidobacterium animalis* subsp. *lactis* | DS27_2 | SAMN08948999 | 1.92047 | 60.5 | 1572 | GCF_003094775.1_ASM309477v1_genomic.fna |
| *Bifidobacterium animalis* subsp. *lactis* | DS24_2 | SAMN08948998 | 1.92495 | 60.5 | 1573 | GCF_003094815.1_ASM309481v1_genomic.fna |
| *Bifidobacterium animalis* subsp. *lactis* | DS23_2 | SAMN08948997 | 1.93413 | 60.5 | 1588 | GCF_003094835.1_ASM309483v1_genomic.fna |
| *Bifidobacterium animalis* subsp. *lactis* | CF3_2 | SAMN08948996 | 1.97324 | 60.6 | 1600 | GCF_003094915.1_ASM309491v1_genomic.fna |
| *Bifidobacterium animalis* subsp. *lactis* | DS28_2 | SAMN08949000 | 1.9188 | 60.5 | 1567 | GCF_003095015.1_ASM309501v1_genomic.fna |
| *Bifidobacterium animalis* subsp. *lactis* | S7 | SAMN07488464 | 1.94407 | 60.5 | 1542 | GCF_003390755.1_ASM339075v1_genomic.fna |
| *Bifidobacterium animalis* subsp. *lactis* | IDCC4301 | SAMN09858435 | 1.94414 | 60.5 | 1580 | GCF_003428375.1_ASM342837v1_genomic.fna |
| *Bifidobacterium animalis* subsp. *lactis* | HN019 | SAMN09684768 | 1.93542 | 60.5 | 1571 | GCF_003606305.1_ASM360630v1_genomic.fna |
| *Bifidobacterium animalis* subsp. *lactis* | UBBLa 70 | SAMN10516502 | 1.94438 | 60 | 1582 | GCF_003970855.1_ASM397085v1_genomic.fna |
| *Bifidobacterium animalis* subsp. *lactis* | 2010B | SAMN10459559 | 1.98248 | 60.3 | 1565 | GCF_004154425.1_ASM415442v1_genomic.fna |
| *Bifidobacterium animalis* subsp. *lactis* | 1821B | SAMN10459555 | 2.00272 | 59.8 | 1639 | GCF_004154435.1_ASM415443v1_genomic.fna |
| *Bifidobacterium animalis* subsp. *lactis* | 1869B | SAMN10459557 | 1.91942 | 60.5 | 1573 | GCF_004154445.1_ASM415444v1_genomic.fna |
| *Bifidobacterium animalis* subsp. *lactis* | 2011B | SAMN10459560 | 2.08307 | 60.4 | 1660 | GCF_004154455.1_ASM415445v1_genomic.fna |
| *Bifidobacterium animalis* subsp. *lactis* | 1843B | SAMN10459556 | 1.91921 | 60.5 | 1574 | GCF_004154475.1_ASM415447v1_genomic.fna |
| *Bifidobacterium animalis* subsp. *lactis* | 1813B | SAMN10459554 | 1.91895 | 60.5 | 1574 | GCF_004154525.1_ASM415452v1_genomic.fna |

| | | | | | | |
|---|---|---|---|---|---|---|
| *Bifidobacterium animalis* subsp. *lactis* | 1811B | SAMN10459553 | 1.92261 | 60.5 | 1573 | GCF_004154535.1_ASM415453v1_genomic.fna |
| *Bifidobacterium animalis* subsp. *lactis* | 1808B | SAMN10459552 | 1.9194 | 60.5 | 1573 | GCF_004154545.1_ASM415454v1_genomic.fna |
| *Bifidobacterium animalis* subsp. *lactis* | 1528B | SAMN10459550 | 1.95396 | 60.6 | 1575 | GCF_004154555.1_ASM415455v1_genomic.fna |
| *Bifidobacterium animalis* subsp. *lactis* | 1316B | SAMN10459548 | 1.91921 | 60.5 | 1573 | GCF_004154565.1_ASM415456v1_genomic.fna |
| *Bifidobacterium animalis* subsp. *lactis* | 2007B | SAMN10459558 | 1.97319 | 60.4 | 1555 | GCF_004154645.1_ASM415464v1_genomic.fna |
| *Bifidobacterium animalis* subsp. *lactis* | 1802B | SAMN10459551 | 1.91948 | 60.5 | 1573 | GCF_004154655.1_ASM415465v1_genomic.fna |
| *Bifidobacterium animalis* subsp. *lactis* | 1395B | SAMN10459549 | 1.91894 | 60.5 | 1574 | GCF_004154695.1_ASM415469v1_genomic.fna |
| ***Bifidobacterium animalis* subsp. *lactis* AD011** | **AD011** | **SAMN02603485** | **1.93369** | **60.5** | **1551** | **GCF_000021425.1_ASM2142v1_genomic.fna** |
| *Bifidobacterium animalis* subsp. *lactis* ATCC 27536 | ATCC 27536 | SAMN03978807 | 1.91245 | 60.5 | 1569 | GCF_001263975.1_ASM126397v1_genomic.fna |
| *Bifidobacterium animalis* subsp. *lactis* ATCC 27673 | ATCC 27673 | SAMN02603715 | 1.96301 | 60.6 | 1523 | GCF_000471945.1_ASM47194v1_genomic.fna |
| *Bifidobacterium animalis* subsp. *lactis* ATCC 27673 | ATCC 27673 | SAMN03978811 | 1.93714 | 60.6 | 1560 | GCF_001264015.1_ASM126401v1_genomic.fna |
| *Bifidobacterium animalis* subsp. *lactis* ATCC 27674 | ATCC 27674 | SAMN03978808 | 1.91238 | 60.5 | 1569 | GCF_001263985.1_ASM126398v1_genomic.fna |
| *Bifidobacterium animalis* subsp. *lactis* B420 | B420 | SAMN02603214 | 1.9386 | 60.5 | 1538 | GCF_000277325.1_ASM27732v1_genomic.fna |
| *Bifidobacterium animalis* subsp. *lactis* BB-12 | BB-12 | SAMN07981101 | 1.99659 | 59.6 | 1495 | GCA_002762435.1_ASM276243v1_genomic.fna |
| *Bifidobacterium animalis* subsp. *lactis* BB-12 | BB-12 | SAMN02603131 | 1.9422 | 60.5 | 1563 | GCF_000025245.1_ASM2524v1_genomic.fna |

| | | | | | | |
|---|---|---|---|---|---|---|
| *Bifidobacterium animalis* subsp. *lactis* Bi-07 | Bi-07 | SAMN02603215 | 1.93882 | 60.5 | 1538 | GCF_000277345.1_ASM27734v1_genomic.fna |
| *Bifidobacterium animalis* subsp. *lactis* Bl-04 | Bl-04; ATCC SD5219 | SAMN02603172 | 1.93871 | 60.5 | 1575 | GCF_000022705.1_ASM2270v1_genomic.fna |
| *Bifidobacterium animalis* subsp. *lactis* Bl12 | Bl12 | SAMN02604238 | 1.93861 | 60.5 | 1574 | GCF_000414215.1_ASM41421v1_genomic.fna |
| *Bifidobacterium animalis* subsp. *lactis* BLC1 | BLC1 | SAMN02604239 | 1.93858 | 60.5 | 1578 | GCF_000224965.2_ASM22496v2_genomic.fna |
| *Bifidobacterium animalis* subsp. *lactis* BS 01 | BS 01 | SAMN00764905 | 1.9326 | 60.5 | 1571 | GCF_000240765.1_BifAniBS01_1.0_genomic.fna |
| *Bifidobacterium animalis* subsp. *lactis* CECT 8145 | CECT 8145 | SAMEA3138972 | 1.95989 | 60.4 | 1569 | GCF_000612705.1_CECT8145_genomic.fna |
| *Bifidobacterium animalis* subsp. *lactis* CNCM I-2494 | CNCM I-2494 | SAMN02604350 | 1.94311 | 60.5 | 1576 | GCF_000220885.1_ASM22088v1_genomic.fna |
| *Bifidobacterium animalis* subsp. *lactis* DSM 10140 | DSM 10140 | SAMN02603171 | 1.93848 | 60.5 | 1566 | GCF_000022965.1_ASM2296v1_genomic.fna |
| *Bifidobacterium animalis* subsp. *lactis* HN019 | HN019 | SAMN02469791 | 1.91589 | 60.5 | 1563 | GCF_000172535.1_Blac_1.0_genomic.fna |
| *Bifidobacterium animalis* subsp. *lactis* KLDS2.0603 | KLDS2.0603 | SAMN02726251 | 1.9469 | 60.5 | 1569 | GCF_000816205.1_ASM81620v1_genomic.fna |
| *Bifidobacterium animalis* subsp. *lactis* V9 | V9 | SAMN02603934 | 1.94405 | 60.5 | 1578 | GCF_000092765.1_ASM9276v1_genomic.fna |
| *Bifidobacterium asteroides* | Bin2 | SAMN03269133 | 2.09478 | 60.6 | 1590 | GCF_000967185.1_ASM96718v1_genomic.fna |
| *Bifidobacterium asteroides* | Bin7 | SAMN03269159 | 2.13239 | 60.7 | 1610 | GCF_000967265.1_ASM96726v1_genomic.fna |
| *Bifidobacterium asteroides* | Hma3 | SAMN03271968 | 2.24522 | 59.5 | 1699 | GCF_000970835.1_ASM97083v1_genomic.fna |
| *Bifidobacterium asteroides* | 1460B | SAMN07731035 | 2.12182 | 60.5 | 1604 | GCF_002846895.1_ASM284689v1_genomic.fna |
| *Bifidobacterium asteroides* | ESL0200 | SAMN08297250 | 1.93342 | 59.5 | 1526 | GCF_003202695.1_ASM320269v1_genomic.fna |
| *Bifidobacterium asteroides* | ESL0198 | SAMN08297248 | 2.23561 | 59.7 | 1724 | GCF_003202715.1_ASM320271v1_genomic.fna |
| *Bifidobacterium asteroides* | ESL0199 | SAMN08297249 | 2.16734 | 59.1 | 1649 | GCF_003202755.1_ASM320275v1_genomic.fna |
| *Bifidobacterium asteroides* | ESL0170 | SAMN08297246 | 2.17526 | 60.1 | 1670 | GCF_003202855.1_ASM320285v1_genomic.fna |

| | | | | | | |
|---|---|---|---|---|---|---|
| *Bifidobacterium asteroides* DSM 20089 | DSM 20089 | SAMN02442012 | 2.13859 | 60 | 1615 | GCF_000771125.1_DSM-20089_genomic.fna |
| *Bifidobacterium asteroides* DSM 20089 | DSM 20089 | SAMN05912982 | 2.19037 | 60 | 1647 | GCF_002715865.1_ASM271586v1_genomic.fna |
| ***Bifidobacterium asteroides* PRL2011** | **PRL2011** | **SAMN02604114** | **2.1673** | **60.1** | **1648** | **GCF_000304215.1_ASM30421v1_genomic.fna** |
| *Bifidobacterium biavatii* DSM 23969 | DSM 23969 | SAMN02673425 | 3.25215 | 63.1 | 2421 | GCF_000741165.1_Bifbia_genomic.fna |
| *Bifidobacterium biavatii* DSM 23969 | DSM 23969 | SAMN02442038 | 3.26268 | 63.2 | 2433 | GCF_000771645.1_DSM-23969_genomic.fna |
| *Bifidobacterium bifidum* | 141.5 | SAMN04497897 | 2.10522 | 62.9 | 1663 | GCA_001576865.1_ASM157686v1_genomic.fna |
| *Bifidobacterium bifidum* | 170.6 | SAMN04497916 | 2.13194 | 63 | 1695 | GCA_001576895.1_ASM157689v1_genomic.fna |
| *Bifidobacterium bifidum* | 62_13 | SAMN05717207 | 1.67851 | 62.8 | 1169 | GCA_001915215.1_ASM191521v1_genomic.fna |
| *Bifidobacterium bifidum* | LMG 11041 | SAMN02673426 | 2.20847 | 62.7 | 1712 | GCF_000741085.1_Bifbif_genomic.fna |
| *Bifidobacterium bifidum* | A8 | SAMN03097401 | 2.2231 | 62.8 | 1687 | GCF_001020245.1_Bbif04v4_genomic.fna |
| *Bifidobacterium bifidum* | 156B | SAMN03097399 | 2.19182 | 62.8 | 1723 | GCF_001020255.1_Bbif02v4_genomic.fna |
| *Bifidobacterium bifidum* | IPLA 20017 | SAMN03097402 | 2.17888 | 62.6 | 1682 | GCF_001020265.1_Bbif05v4_genomic.fna |
| *Bifidobacterium bifidum* | LMG 11582 | SAMN03097403 | 2.2225 | 62.8 | 1675 | GCF_001020275.1_Bbif07v4_genomic.fna |
| *Bifidobacterium bifidum* | LMG 13200 | SAMN03097405 | 2.17604 | 62.9 | 1685 | GCF_001020325.1_Bbif10v4_genomic.fna |
| *Bifidobacterium bifidum* | LMG 11583 | SAMN03097404 | 2.23853 | 62.9 | 1765 | GCF_001020335.1_Bbif08v4_genomic.fna |
| *Bifidobacterium bifidum* | 85B | SAMN03097398 | 2.36097 | 63 | 1828 | GCF_001020355.1_Bbif01v4_genomic.fna |
| *Bifidobacterium bifidum* | 324B | SAMN03097400 | 2.20434 | 62.7 | 1768 | GCF_001020375.1_Bbif03v4_genomic.fna |
| *Bifidobacterium bifidum* | IPLA 20015 | SAMN03103719 | 2.17299 | 62.7 | 1672 | GCF_001020405.1_Bbif06v4_genomic.fna |
| *Bifidobacterium bifidum* | BF3 | SAMN03271680 | 2.21037 | 62.6 | 1696 | GCF_001281345.1_ASM128134v1_genomic.fna |
| *Bifidobacterium bifidum* | 2789STDY5608877 | SAMEA3545285 | 2.15716 | 62.8 | 1669 | GCF_001405355.1_13414_6_61_genomic.fna |
| *Bifidobacterium bifidum* | MJR8628B | SAMN03842220 | 2.26235 | 62.8 | 1814 | GCF_001546225.1_ASM154622v1_genomic.fna |
| *Bifidobacterium bifidum* | 791 | SAMN04155634 | 2.28546 | 62.4 | 1778 | GCF_001595435.1_ASM159543v1_genomic.fna |
| *Bifidobacterium bifidum* | BI-14 | SAMD00047595 | 2.18443 | 62.6 | 1658 | GCF_001685685.1_ASM168568v1_genomic.fna |
| *Bifidobacterium bifidum* | 1887B | SAMN06621707 | 2.25554 | 62.6 | 1777 | GCF_002076105.1_Bbif1887B_genomic.fna |
| *Bifidobacterium bifidum* | ICIS-310 | SAMN05858649 | 2.21963 | 62.4 | 1715 | GCF_002114145.1_ASM211414v1_genomic.fna |

| | | | | | | |
|---|---|---|---|---|---|---|
| *Bifidobacterium bifidum* | 1 G1971 | SAMN06759457 | 2.19803 | 62.7 | 1538 | GCF_002469385.1_ASM246938v1_genomic.fna |
| *Bifidobacterium bifidum* | PRI 1 | SAMN06140833 | 2.24357 | 62.7 | 1718 | GCF_002845845.1_ASM284584v1_genomic.fna |
| *Bifidobacterium bifidum* | DS33_23 | SAMN08949006 | 2.22957 | 62.8 | 1714 | GCF_003094645.1_ASM309464v1_genomic.fna |
| *Bifidobacterium bifidum* | DS32_23 | SAMN08949005 | 2.27993 | 62.9 | 1789 | GCF_003094715.1_ASM309471v1_genomic.fna |
| *Bifidobacterium bifidum* | DS31_23 | SAMN08949004 | 2.23088 | 62.8 | 1723 | GCF_003094735.1_ASM309473v1_genomic.fna |
| *Bifidobacterium bifidum* | DS30_23 | SAMN08949003 | 2.25422 | 62.8 | 1772 | GCF_003094755.1_ASM309475v1_genomic.fna |
| *Bifidobacterium bifidum* | DS25_23 | SAMN08949001 | 2.22666 | 62.7 | 1724 | GCF_003094795.1_ASM309479v1_genomic.fna |
| *Bifidobacterium bifidum* | DS26_23 | SAMN08949002 | 2.23188 | 62.8 | 1712 | GCF_003095035.1_ASM309503v1_genomic.fna |
| *Bifidobacterium bifidum* | S6 | SAMN07484235 | 2.31134 | 62.7 | 1771 | GCF_003390735.1_ASM339073v1_genomic.fna |
| *Bifidobacterium bifidum* | TM07-4AC | SAMN09737006 | 2.18181 | 62.8 | 1690 | GCF_003436135.1_ASM343613v1_genomic.fna |
| *Bifidobacterium bifidum* | TM04-12 | SAMN09736968 | 2.31404 | 62.7 | 1827 | GCF_003436575.1_ASM343657v1_genomic.fna |
| *Bifidobacterium bifidum* | TM02-17 | SAMN09736965 | 2.20117 | 62.6 | 1690 | GCF_003436635.1_ASM343663v1_genomic.fna |
| *Bifidobacterium bifidum* | TM02-15 | SAMN09736964 | 2.20032 | 62.6 | 1686 | GCF_003436655.1_ASM343665v1_genomic.fna |
| *Bifidobacterium bifidum* | TF07-22 | SAMN09736904 | 2.19143 | 62.6 | 1706 | GCF_003437175.1_ASM343717v1_genomic.fna |
| *Bifidobacterium bifidum* | TF06-14AC | SAMN09736890 | 2.16607 | 62.7 | 1668 | GCF_003437325.1_ASM343732v1_genomic.fna |
| *Bifidobacterium bifidum* | TF06-13 | SAMN09736889 | 2.16628 | 62.7 | 1667 | GCF_003437345.1_ASM343734v1_genomic.fna |
| *Bifidobacterium bifidum* | TF05-39 | SAMN09736884 | 2.17895 | 62.8 | 1673 | GCF_003437785.1_ASM343778v1_genomic.fna |
| *Bifidobacterium bifidum* | TF05-1 | SAMN09736871 | 2.32917 | 62.5 | 1840 | GCF_003437945.1_ASM343794v1_genomic.fna |
| *Bifidobacterium bifidum* | AF11-25B | SAMN09734209 | 2.23403 | 62.6 | 1736 | GCF_003465425.1_ASM346542v1_genomic.fna |
| *Bifidobacterium bifidum* | TM06-5 | SAMN09736996 | 2.20388 | 62.6 | 1707 | GCF_003466365.1_ASM346636v1_genomic.fna |
| *Bifidobacterium bifidum* | TM06-10 | SAMN09736987 | 2.20702 | 62.6 | 1710 | GCF_003466395.1_ASM346639v1_genomic.fna |
| *Bifidobacterium bifidum* | TM05-15 | SAMN09736979 | 2.17737 | 62.8 | 1675 | GCF_003466485.1_ASM346648v1_genomic.fna |
| *Bifidobacterium bifidum* | AM42-15AC | SAMN09736543 | 2.30945 | 62.6 | 1809 | GCF_003467985.1_ASM346798v1_genomic.fna |
| *Bifidobacterium bifidum* | AM36-1AC | SAMN09736479 | 2.15368 | 62.8 | 1684 | GCF_003468415.1_ASM346841v1_genomic.fna |
| *Bifidobacterium bifidum* | AM18-29 | SAMN09734868 | 2.20117 | 62.8 | 1702 | GCF_003470585.1_ASM347058v1_genomic.fna |
| *Bifidobacterium bifidum* | AM18-12AC | SAMN09734861 | 2.20416 | 62.8 | 1702 | GCF_003470615.1_ASM347061v1_genomic.fna |
| *Bifidobacterium bifidum* | AM18-11 | SAMN09734860 | 2.20457 | 62.8 | 1705 | GCF_003471555.1_ASM347155v1_genomic.fna |
| *Bifidobacterium bifidum* | AM18-1 | SAMN09734857 | 2.20293 | 62.8 | 1703 | GCF_003471595.1_ASM347159v1_genomic.fna |
| *Bifidobacterium bifidum* | AM12-10 | SAMN09734784 | 2.37548 | 62.5 | 1902 | GCF_003472345.1_ASM347234v1_genomic.fna |

| | | | | | | |
|---|---|---|---|---|---|---|
| *Bifidobacterium bifidum* | AM12-9 | SAMN09734792 | 2.37333 | 62.5 | 1904 | GCF_003473145.1_ASM347314v1_genomic.fna |
| *Bifidobacterium bifidum* | AF45-10 | SAMN09734712 | 2.16192 | 62.7 | 1412 | GCF_003474045.1_ASM347404v1_genomic.fna |
| *Bifidobacterium bifidum* | TMC 3115 | SAMD00078313 | 2.17889 | 62.8 | 1612 | GCF_003573895.1_ASM357389v1_genomic.fna |
| *Bifidobacterium bifidum* | ICIS-643 | SAMN06606988 | 2.28172 | 62.4 | 1766 | GCF_003790385.1_ASM379038v1_genomic.fna |
| *Bifidobacterium bifidum* | ca_0067 | SAMN10239585 | 2.18861 | 62.7 | 1734 | GCF_004167365.1_ASM416736v1_genomic.fna |
| *Bifidobacterium bifidum* | ICIS-202 | SAMN07709009 | 2.26506 | 62.4 | 1763 | GCF_004799295.1_ASM479929v1_genomic.fna |
| *Bifidobacterium bifidum* | BSD2780061688st1_G1 | SAMN10863272 | 2.16421 | 62.7 | - | GCF_005844205.1_ASM584420v1_genomic.fna |
| *Bifidobacterium bifidum* | 1001271st1_H11 | SAMN10863260 | 2.26527 | 62.7 | - | GCF_005844365.1_ASM584436v1_genomic.fna |
| *Bifidobacterium bifidum* | Calf96 | SAMN05216254 | 2.1503 | 62.8 | 1670 | GCF_900112465.1_IMG-taxon_2654588208_annotated_assembly_genomic.fna |
| *Bifidobacterium bifidum* | NCTC13001 | SAMEA4063017 | 2.21103 | 62.7 | 1736 | GCF_900637095.1_48450_G02_genomic.fna |
| Bifidobacterium bifidum ATCC 29521 = JCM 1255 = DSM 20456 | ATCC 29521 | SAMN02436821 | 2.20125 | 62.7 | 1713 | GCF_000466525.1_ASM46652v1_genomic.fna |
| *Bifidobacterium bifidum* ATCC 29521 = JCM 1255 = DSM 20456 | DSM 20456 | SAMN02442030 | 2.20279 | 62.7 | 1721 | GCF_000771485.1_DSM-20456_genomic.fna |
| *Bifidobacterium bifidum* ATCC 29521 = JCM 1255 = DSM 20456 | JCM 1255 | SAMD00061040 | 2.21104 | 62.7 | 1723 | GCF_001025135.1_ASM102513v1_genomic.fna |
| *Bifidobacterium bifidum* BGN4 | BGN4 | SAMN02603486 | 2.22366 | 62.6 | 1727 | GCF_000265095.1_ASM26509v1_genomic.fna |
| *Bifidobacterium bifidum* IPLA 20015 | IPLA 20015 | SAMN02470078 | 2.13833 | 62.7 | 1553 | GCF_000300215.1_ASM30021v1_genomic.fna |
| *Bifidobacterium bifidum* JCM 1254 | JCM 1254 | SAMD00000434 | 2.19002 | 62.6 | - | GCF_001311705.1_ASM131170v1_genomic.fna |
| *Bifidobacterium bifidum* LMG 13195 | JCM 7004 | SAMD00078038 | 2.26167 | 62.6 | 2106 | GCA_003573955.1_ASM357395v1_genomic.fna |
| *Bifidobacterium bifidum* LMG 13195 | LMG 13195 | SAMN02470080 | 2.28315 | 62.6 | 1730 | GCF_000299595.1_ASM29959v1_genomic.fna |
| *Bifidobacterium bifidum* LMG 13195 | LMG 13195 | SAMN03103720 | 2.31018 | 62.6 | 1772 | GCF_001020415.1_Bbif09v4_genomic.fna |
| *Bifidobacterium bifidum* NCIMB 41171 | NCIMB 41171 | SAMN02463676 | 2.20269 | 62.7 | 1738 | GCF_000155395.1_ASM15539v1_genomic.fna |

| | | | | | | |
|---|---|---|---|---|---|---|
| *Bifidobacterium bifidum* NCIMB 41171 | NCIMB 41171 | SAMN02463676 | 2.21639 | 62.8 | 1755 | GCF_000273525.1_PB_Bifi_bifi_NCIMB_41171_V1_genomic.fna |
| ***Bifidobacterium bifidum* PRL2010** | **PRL2010** | **SAMN02603510** | **2.21466** | **62.7** | **1706** | **GCF_000165905.1_ASM16590v1_genomic.fna** |
| *Bifidobacterium bifidum* S17 | S17 | SAMN02603395 | 2.18688 | 62.8 | 1715 | GCF_000164965.1_ASM16496v1_genomic.fna |
| *Bifidobacterium bohemicum* | R-53250 | SAMN04487797 | 2.04645 | 57.4 | 1526 | GCF_900094965.1_IMG-taxon_2616644833_annotated_assembly_genomic.fna |
| *Bifidobacterium bohemicum* DSM 22767 | DSM 22767 | SAMN02673427 | 2.05247 | 57.5 | 1534 | GCF_000741525.1_Bifboh_genomic.fna |
| *Bifidobacterium bohemicum* DSM 22767 | DSM 22767 | SAMN02442036 | 2.0549 | 57.4 | 1522 | GCF_000771605.1_DSM-22767_genomic.fna |
| *Bifidobacterium bombi* DSM 19703 | DSM 19703 | SAMN02952076 | 1.89524 | 56.1 | 1403 | GCF_000737845.1_BBOMBassembly_genomic.fna |
| *Bifidobacterium bombi* DSM 19703 | DSM 19703 | SAMN02442010 | 1.91279 | 56 | 1418 | GCF_000771085.1_DSM-19703_genomic.fna |
| *Bifidobacterium boum* | LMG 10736 | SAMN02673428 | 2.17136 | 59.3 | 1645 | GCF_000741535.1_Bifbou_genomic.fna |
| *Bifidobacterium boum* DSM 20432 | DSM 20432 | SAMN02743876 | 2.16119 | 59.3 | 1642 | GCF_000687615.1_ASM68761v1_genomic.fna |
| *Bifidobacterium boum* DSM 20432 | DSM 20432 | SAMN02442025 | 2.17144 | 59.3 | 1642 | GCF_000771385.1_DSM-20432_genomic.fna |
| *Bifidobacterium breve* | 141.3 | SAMN04497912 | 1.09419 | 59.2 | 868 | GCA_001576875.1_ASM157687v1_genomic.fna |
| *Bifidobacterium breve* | LMG 13208 | SAMN02673429 | 2.26378 | 58.9 | 1798 | GCF_000741125.1_Bifbre_genomic.fna |
| *Bifidobacterium breve* | BBRI4 | SAMN03777307 | 2.42672 | 58.7 | 1964 | GCF_001189355.1_assBREI4_genomic.fna |
| *Bifidobacterium breve* | BR3 | SAMN03271681 | 2.42601 | 59.1056 | 1987 | GCF_001281425.1_ASM128142v1_genomic.fna |
| *Bifidobacterium breve* | GED8481 | SAMN03842221 | 2.35844 | 58.6 | 1909 | GCF_001546235.1_ASM154623v1_genomic.fna |
| *Bifidobacterium breve* | BR-06 | SAMD00047596 | 2.6608 | 59.2 | 2066 | GCF_001685705.1_ASM168570v1_genomic.fna |
| *Bifidobacterium breve* | BR-07 | SAMD00047597 | 2.24948 | 58.7 | 1809 | GCF_001685725.1_ASM168572v1_genomic.fna |
| *Bifidobacterium breve* | BR-10 | SAMD00047598 | 2.34992 | 58.8 | 1860 | GCF_001685745.1_ASM168574v1_genomic.fna |
| *Bifidobacterium breve* | BR-14 | SAMD00047599 | 2.52987 | 59.2 | 2086 | GCF_001685765.1_ASM168576v1_genomic.fna |
| *Bifidobacterium breve* | BR-15 | SAMD00047600 | 2.3738 | 58.7 | 1890 | GCF_001685785.1_ASM168578v1_genomic.fna |
| *Bifidobacterium breve* | BR-19 | SAMD00047601 | 2.32499 | 58.6 | 1867 | GCF_001685805.1_ASM168580v1_genomic.fna |
| *Bifidobacterium breve* | BR-20 | SAMD00047602 | 2.33547 | 58.8 | 1848 | GCF_001685825.1_ASM168582v1_genomic.fna |
| *Bifidobacterium breve* | BR-21 | SAMD00047603 | 2.61462 | 59.2 | 2151 | GCF_001685845.1_ASM168584v1_genomic.fna |
| *Bifidobacterium breve* | BR-A29 | SAMD00047604 | 2.38763 | 58.8 | 1881 | GCF_001685865.1_ASM168586v1_genomic.fna |
| *Bifidobacterium breve* | BR-C29 | SAMD00047605 | 2.28766 | 58.9 | 1844 | GCF_001685885.1_ASM168588v1_genomic.fna |

| | | | | | | |
|---|---|---|---|---|---|---|
| *Bifidobacterium breve* | BR-H29 | SAMD00047606 | 2.47084 | 59.2 | 1990 | GCF_001685905.1_ASM168590v1_genomic.fna |
| *Bifidobacterium breve* | BR-I29 | SAMD00047607 | 2.24992 | 58.7 | 1797 | GCF_001685925.1_ASM168592v1_genomic.fna |
| *Bifidobacterium breve* | BR-L29 | SAMD00047608 | 2.34466 | 59 | 1921 | GCF_001685945.1_ASM168594v1_genomic.fna |
| *Bifidobacterium breve* | LMC520 | SAMN06240351 | 2.4034 | 59 | 1953 | GCF_001990225.1_ASM199022v1_genomic.fna |
| *Bifidobacterium breve* | CECT7263 | SAMN06473336 | 2.33041 | 58.9 | 1860 | GCF_002027265.1_ASM202726v1_genomic.fna |
| *Bifidobacterium breve* | 1900B | SAMN06621718 | 2.28787 | 58.6 | 1844 | GCF_002075865.1_Bbif1900B_genomic.fna |
| *Bifidobacterium breve* | 1891B | SAMN06621711 | 2.41898 | 59.1 | 1972 | GCF_002076055.1_Bbif1891B_genomic.fna |
| *Bifidobacterium breve* | 1889B | SAMN06621709 | 2.34482 | 58.8 | 1866 | GCF_002076075.1_Bbif1889B_genomic.fna |
| *Bifidobacterium breve* | 7E | SAMN05933031 | 2.27086 | 58.6 | 1794 | GCF_002271275.1_Bbre7Ev1_genomic.fna |
| *Bifidobacterium breve* | DRBB26 | SAMN06827973 | 2.39639 | 58.5 | 1936 | GCF_002838225.1_ASM283822v1_genomic.fna |
| *Bifidobacterium breve* | NRBB01 | SAMN06827008 | 2.2694 | 58.9 | 1884 | GCF_002838245.1_ASM283824v1_genomic.fna |
| *Bifidobacterium breve* | NRBB02 | SAMN06827019 | 2.28988 | 58.6 | 1826 | GCF_002838265.1_ASM283826v1_genomic.fna |
| *Bifidobacterium breve* | NRBB04 | SAMN06827050 | 2.32465 | 58.7 | 1844 | GCF_002838285.1_ASM283828v1_genomic.fna |
| *Bifidobacterium breve* | NRBB11 | SAMN06827081 | 2.37756 | 58.7 | 1875 | GCF_002838305.1_ASM283830v1_genomic.fna |
| *Bifidobacterium breve* | NRBB09 | SAMN06827067 | 2.26556 | 58.7 | 1790 | GCF_002838325.1_ASM283832v1_genomic.fna |
| *Bifidobacterium breve* | NRBB57 | SAMN06827912 | 2.51038 | 59.4 | 2057 | GCF_002838345.1_ASM283834v1_genomic.fna |
| *Bifidobacterium breve* | NRBB50 | SAMN06827094 | 2.40906 | 58.8 | 1979 | GCF_002838365.1_ASM283836v1_genomic.fna |
| *Bifidobacterium breve* | NRBB52 | SAMN06827097 | 2.37967 | 58.9 | 1931 | GCF_002838385.1_ASM283838v1_genomic.fna |
| *Bifidobacterium breve* | NRBB51 | SAMN06827096 | 2.40227 | 59 | 1914 | GCF_002838405.1_ASM283840v1_genomic.fna |
| *Bifidobacterium breve* | NRBB56 | SAMN06827911 | 2.42512 | 58.9 | 1940 | GCF_002838425.1_ASM283842v1_genomic.fna |
| *Bifidobacterium breve* | DRBB27 | SAMN06827975 | 2.43508 | 58.9 | 1986 | GCF_002838445.1_ASM283844v1_genomic.fna |
| *Bifidobacterium breve* | 017W439 | SAMN06828931 | 2.30142 | 58.7 | 1855 | GCF_002838465.1_ASM283846v1_genomic.fna |
| *Bifidobacterium breve* | 215W447a | SAMN06829004 | 2.5896 | 59.3 | 2126 | GCF_002838485.1_ASM283848v1_genomic.fna |
| *Bifidobacterium breve* | DRBB28 | SAMN06827976 | 2.46217 | 59 | 2031 | GCF_002838505.1_ASM283850v1_genomic.fna |
| *Bifidobacterium breve* | 180W83 | SAMN06829003 | 2.27317 | 58.8 | 1837 | GCF_002838525.1_ASM283852v1_genomic.fna |
| *Bifidobacterium breve* | 082W48 | SAMN06828932 | 2.28634 | 58.8 | 1842 | GCF_002838545.1_ASM283854v1_genomic.fna |
| *Bifidobacterium breve* | 139W423 | SAMN06829002 | 2.41128 | 58.6 | 1940 | GCF_002838565.1_ASM283856v1_genomic.fna |
| *Bifidobacterium breve* | CNCM I-4321 | SAMN06827913 | 2.46485 | 59 | 2045 | GCF_002838585.1_ASM283858v1_genomic.fna |
| *Bifidobacterium breve* | NRBB18 | SAMN07160576 | 2.28969 | 58.6 | 1829 | GCF_002838605.1_ASM283860v1_genomic.fna |

| | | | | | | |
|---|---|---|---|---|---|---|
| *Bifidobacterium breve* | NRBB19 | SAMN07160980 | 2.28973 | 58.6 | 1813 | GCF_002838625.1_ASM283862v1_genomic.fna |
| *Bifidobacterium breve* | NRBB20 | SAMN07160580 | 2.28989 | 58.6 | 1833 | GCF_002838645.1_ASM283864v1_genomic.fna |
| *Bifidobacterium breve* | NRBB27 | SAMN07160976 | 2.28984 | 58.6 | 1826 | GCF_002838665.1_ASM283866v1_genomic.fna |
| *Bifidobacterium breve* | NRBB49 | SAMN07160977 | 2.28979 | 58.6 | 1828 | GCF_002838685.1_ASM283868v1_genomic.fna |
| *Bifidobacterium breve* | DRBB29 | SAMN07160978 | 2.43509 | 58.9 | 1987 | GCF_002838705.1_ASM283870v1_genomic.fna |
| *Bifidobacterium breve* | DRBB30 | SAMN07160979 | 2.47112 | 58.9 | 2051 | GCF_002838725.1_ASM283872v1_genomic.fna |
| *Bifidobacterium breve* | NRBB08 | SAMN07160574 | 2.28976 | 58.6 | 1820 | GCF_002838745.1_ASM283874v1_genomic.fna |
| *Bifidobacterium breve* | UMB0915 | SAMN08193648 | 2.315 | 58.8 | 1861 | GCF_002861455.1_ASM286145v1_genomic.fna |
| *Bifidobacterium breve* | UMB0089 | SAMN07511392 | 2.33608 | 58.8 | 1868 | GCF_002871815.1_ASM287181v1_genomic.fna |
| *Bifidobacterium breve* | LMG S-29190 | SAMN07187787 | 2.3172 | 58.8 | 1877 | GCF_002914865.1_ASM291486v1_genomic.fna |
| *Bifidobacterium breve* | DS15_17 | SAMN06464121 | 2.26068 | 58.9 | 1891 | GCF_003095095.1_ASM309509v1_genomic.fna |
| *Bifidobacterium breve* | N6D12 | SAMN07189843 | 2.29032 | 58.6 | 1768 | GCF_003370105.1_ASM337010v1_genomic.fna |
| *Bifidobacterium breve* | W20-13 | SAMN07189841 | 2.29515 | 58.5 | 1819 | GCF_003370125.1_ASM337012v1_genomic.fna |
| *Bifidobacterium breve* | 43803 | SAMN07189840 | 2.26034 | 58.2 | 1610 | GCF_003370155.1_ASM337015v1_genomic.fna |
| *Bifidobacterium breve* | 322-1 | SAMN07189839 | 2.23684 | 58.7 | 1741 | GCF_003370175.1_ASM337017v1_genomic.fna |
| *Bifidobacterium breve* | 43495 | SAMN07189837 | 2.53953 | 59.1 | 1743 | GCF_003370195.1_ASM337019v1_genomic.fna |
| *Bifidobacterium breve* | W56 | SAMN07189842 | 2.36405 | 58.5 | 1831 | GCF_003370265.1_ASM337026v1_genomic.fna |
| *Bifidobacterium breve* | 33239 | SAMN07189838 | 2.23926 | 58.5 | 1574 | GCF_003370295.1_ASM337029v1_genomic.fna |
| *Bifidobacterium breve* | FDAARGOS_561 | SAMN10163185 | 2.27565 | 58.9 | 1903 | GCF_003813065.1_ASM381306v1_genomic.fna |
| *Bifidobacterium breve* | lw01 | SAMN10484345 | 2.31317 | 58.8 | 1862 | GCF_003860285.1_ASM386028v1_genomic.fna |
| *Bifidobacterium breve* | BR03 | SAMN10659766 | 2.27463 | 58.6 | 1807 | GCF_004319685.1_ASM431968v1_genomic.fna |
| *Bifidobacterium breve* | UBBR-01 | SAMN11381953 | 2.33383 | 58.7 | 1908 | GCF_004802595.1_ASM480259v1_genomic.fna |
| *Bifidobacterium breve* | RP2 | SAMN05216468 | 2.26049 | 58.7 | 1816 | GCF_900102865.1_IMG-taxon_2651870312_annotated_assembly_genomic.fna |
| *Bifidobacterium breve* | Bifido_10 | SAMEA51822418 | 2.37804 | 58.5 | 1934 | GCF_900157105.1_Bifido_10_v1_genomic.fna |
| *Bifidobacterium breve* | Bifido_07 | SAMEA51820168 | 2.34387 | 58.8 | 1926 | GCF_900157125.1_Bifido_07_v1_genomic.fna |
| *Bifidobacterium breve* | NCTC11815 | SAMEA4076721 | 2.27566 | 58.9 | 1901 | GCF_900637145.1_49569_E01_genomic.fna |
| *Bifidobacterium breve* 12L | 12L | SAMN03081478 | 2.24462 | 58.9 | 1820 | GCF_000568955.1_ASM56895v1_genomic.fna |
| *Bifidobacterium breve* 2L | 2L | SAMN02951890 | 2.24072 | 58.9 | 1761 | GCF_000568895.1_Mira_de-novo_assembly_genomic.fna |

| | | | | | | |
|---|---|---|---|---|---|---|
| *Bifidobacterium breve* 31L | 31L | SAMN02951889 | 2.26565 | 58.6 | 1689 | GCF_000568875.1_Mira_de-novo_assembly_genomic.fna |
| *Bifidobacterium breve* 689b | 689b | SAMN03081482 | 2.33171 | 58.7 | 1887 | GCF_000569055.1_ASM56905v1_genomic.fna |
| *Bifidobacterium breve* ACS-071-V-Sch8b | ACS-071-V-Sch8b | SAMN00100758 | 2.32749 | 58.7 | 1877 | GCF_000213865.1_ASM21386v1_genomic.fna |
| *Bifidobacterium breve* CECT 7263 | CECT 7263 | SAMN02471362 | 2.3144 | 58.9 | 1841 | GCF_000247755.1_ASM24775v2_genomic.fna |
| *Bifidobacterium breve* DPC 6330 | DPC 6330 | SAMN02471030 | 2.38623 | 58.6 | 1977 | GCF_000226175.1_ASM22617v2_genomic.fna |
| *Bifidobacterium breve* DSM 20213 = JCM 1192 | DSM 20213 | SAMN00008778 | 2.33139 | 58.5 | 1927 | GCF_000158015.1_ASM15801v1_genomic.fna |
| *Bifidobacterium breve* DSM 20213 = JCM 1192 | DSM 20213 | SAMN02442021 | 2.25713 | 58.9 | 1895 | GCF_000771305.1_DSM-20213_genomic.fna |
| ***Bifidobacterium breve* DSM 20213 = JCM 1192** | **JCM 1192** | **SAMD00061041** | **2.26941** | **58.9** | **1904** | **GCF_001025175.1_ASM102517v1_genomic.fna** |
| *Bifidobacterium breve* DSM 20213 = JCM 1192 | JCM 1192 | SAMD00000422 | 2.23281 | 58.7 | - | GCF_001311295.1_ASM131129v1_genomic.fna |
| *Bifidobacterium breve* HPH0326 | HPH0326 | SAMN02596976 | 2.50431 | 59.1 | 2047 | GCF_000411435.1_Bifi_brev_HPH0326_V1_genomic.fna |
| *Bifidobacterium breve* JCM 7017 | JCM 7017 | SAMN03081479 | 2.28892 | 58.7 | 1843 | GCF_000568975.1_ASM56897v1_genomic.fna |
| *Bifidobacterium breve* JCM 7019 | JCM 7019 | SAMN03081480 | 2.35901 | 58.6 | 1919 | GCF_000569015.1_ASM56901v1_genomic.fna |
| *Bifidobacterium breve* JCP7499 | JCP7499 | SAMN02436887 | 2.36972 | 58.6 | 1913 | GCF_000466545.1_ASM46654v1_genomic.fna |
| *Bifidobacterium breve* MCC 0121 | MCC 0121 | SAMN03978796 | 2.43634 | 58.9 | 1973 | GCF_001264045.1_ASM126404v1_genomic.fna |
| *Bifidobacterium breve* MCC 0305 | MCC 0305 | SAMN03978801 | 2.28684 | 58.8 | 1859 | GCF_001264035.1_ASM126403v1_genomic.fna |
| *Bifidobacterium breve* MCC 0476 | MCC 0476 | SAMN03978797 | 2.23381 | 58.6 | 1800 | GCF_001263845.1_ASM126384v1_genomic.fna |
| *Bifidobacterium breve* MCC 1094 | MCC 1094 | SAMN03978802 | 2.32721 | 59 | 1899 | GCF_001264095.1_ASM126409v1_genomic.fna |
| *Bifidobacterium breve* MCC 1114 | MCC 1114 | SAMN03978798 | 2.48728 | 59 | 2018 | GCF_001263855.1_ASM126385v1_genomic.fna |
| *Bifidobacterium breve* MCC 1128 | MCC 1128 | SAMN03978799 | 2.48022 | 58.9 | 2045 | GCF_001263915.1_ASM126391v1_genomic.fna |
| *Bifidobacterium breve* MCC 1340 | MCC 1340 | SAMN03978803 | 2.37311 | 58.6 | 1899 | GCF_001264105.1_ASM126410v1_genomic.fna |
| *Bifidobacterium breve* MCC 1454 | MCC 1454 | SAMN03978804 | 2.45745 | 58.5 | 2034 | GCF_001264135.1_ASM126413v1_genomic.fna |
| *Bifidobacterium breve* MCC 1604 | MCC 1604 | SAMN03978800 | 2.20639 | 58.7 | 1771 | GCF_001264155.1_ASM126415v1_genomic.fna |
| *Bifidobacterium breve* MCC 1605 | MCC 1605 | SAMN03978805 | 2.32401 | 59 | 1870 | GCF_001263935.1_ASM126393v1_genomic.fna |
| *Bifidobacterium breve* NCFB 2258 | NCFB 2258 | SAMN03081481 | 2.3159 | 58.7 | 1870 | GCF_000569035.1_ASM56903v1_genomic.fna |
| *Bifidobacterium breve* S27 | S27 | SAMN03081483 | 2.29446 | 58.7 | 1837 | GCF_000569075.1_ASM56907v1_genomic.fna |

| | | | | | | |
|---|---|---|---|---|---|---|
| *Bifidobacterium breve* UCC2003 | UCC2003 | SAMN02604112 | 2.42268 | 58.7 | 1960 | GCF_000220135.1_ASM22013v1_genomic.fna |
| *Bifidobacterium callitrichos* | UMA51805 | SAMN07664461 | 2.77709 | 63.6 | 2110 | GCF_003024945.1_ASM302494v1_genomic.fna |
| *Bifidobacterium callitrichos* | UMA51804 | SAMN07664420 | 3.03233 | 64.5 | 2371 | GCF_003024955.1_ASM302495v1_genomic.fna |
| *Bifidobacterium callitrichos* DSM 23973 | DSM 23973 | SAMN02673430 | 2.88731 | 63.5 | 2217 | GCF_000741175.1_Bifcal_genomic.fna |
| *Bifidobacterium callitrichos* DSM 23973 | DSM 23973 | SAMN02442039 | 2.86721 | 63.6 | 2243 | GCF_000771665.1_DSM-23973_genomic.fna |
| *Bifidobacterium catenulatum* | 1899B | SAMN06621717 | 2.1246 | 56.3 | 1669 | GCF_002075855.1_Bbif1899B_genomic.fna |
| *Bifidobacterium catenulatum* DSM 16992 = JCM 1194 = LMG 11043 | DSM 16992 | SAMN00008795 | 2.05843 | 56.1 | 1619 | GCF_000173455.1_ASM17345v1_genomic.fna |
| *Bifidobacterium catenulatum* DSM 16992 = JCM 1194 = LMG 11043 | LMG 11043 | SAMN02673431 | 2.08276 | 56.1 | 1515 | GCF_000741565.1_Bifcat_genomic.fna |
| *Bifidobacterium catenulatum* DSM 16992 = JCM 1194 = LMG 11043 | DSM 16992 | SAMN02442007 | 2.10636 | 56.4 | 1638 | GCF_000771025.1_DSM-16992_genomic.fna |
| *Bifidobacterium catenulatum* DSM 16992 = JCM 1194 = LMG 11043 | JCM 1194 | SAMD00061042 | 2.07952 | 56.2 | 1628 | GCF_001025195.1_ASM102519v1_genomic.fna |
| *Bifidobacterium choerinum* | LMG 10510 | SAMN02673432 | 2.09612 | 65.5 | 1591 | GCF_000741135.1_Bifcho_genomic.fna |
| *Bifidobacterium choerinum* | FMB-1 | SAMN05949246 | 2.25729 | 65.5517 | 1758 | GCF_002761235.1_ASM276123v1_genomic.fna |
| *Bifidobacterium choerinum* DSM 20434 | DSM 20434 | SAMN02441446 | 2.03732 | 65.7 | 1544 | GCF_000484675.1_ASM48467v1_genomic.fna |
| *Bifidobacterium choerinum* DSM 20434 | DSM 20434 | SAMN02442027 | 2.04994 | 65.7 | 1536 | GCF_000771425.1_DSM-20434_genomic.fna |
| *Bifidobacterium coryneforme* | LMG18911 | SAMN02666223 | 1.75515 | 60.5 | 1352 | GCF_000737865.1_ASM73786v1_genomic.fna |
| *Bifidobacterium coryneforme* | Bma6 | SAMN03271958 | 1.74971 | 60.5 | 1342 | GCF_000967205.1_ASM96720v1_genomic.fna |
| *Bifidobacterium coryneforme* DSM 20216 | DSM 20216 | SAMN02442023 | 1.741 | 60.5 | 1343 | GCF_000771345.1_DSM-20216_genomic.fna |
| *Bifidobacterium cuniculi* | LMG 10738 | SAMN02673433 | 2.53159 | 64.9 | 2105 | GCF_000741575.1_Bifcun_genomic.fna |
| *Bifidobacterium cuniculi* DSM 20435 | DSM 20435 | SAMN02442028 | 2.51269 | 64.9 | 2069 | GCF_000771465.1_DSM-20435_genomic.fna |
| *Bifidobacterium dentium* | 176.7 | SAMN04497915 | 1.40795 | 58.4 | 1096 | GCA_001576945.1_ASM157694v1_genomic.fna |

| | | | | | | |
|---|---|---|---|---|---|---|
| *Bifidobacterium dentium* | UBA10777 | SAMN08018834 | 2.26612 | 58.5 | 1649 | GCA_003508295.1_ASM350829v1_genomic.fna |
| *Bifidobacterium dentium* | DE-29 | SAMD00047615 | 2.55269 | 58.5 | 2016 | GCF_001686085.1_ASM168608v1_genomic.fna |
| *Bifidobacterium dentium* | 1893B | SAMN06621713 | 2.57107 | 58.2 | 2033 | GCF_002075955.1_Bbif1893B_genomic.fna |
| *Bifidobacterium dentium* | cx_0004 | SAMN10239586 | 2.58988 | 58.5 | 2098 | GCF_004167735.1_ASM416773v1_genomic.fna |
| *Bifidobacterium dentium* | ATCC 15424 | SAMN11280666 | 2.62554 | 58.5 | 2154 | GCF_004683745.1_ASM468374v1_genomic.fna |
| *Bifidobacterium dentium* | NCTC11816 | SAMEA4076722 | 2.63583 | 58.5 | 2120 | GCF_900637175.1_49569_F01_genomic.fna |
| *Bifidobacterium dentium* ATCC 27678 | ATCC 27678 | SAMN00000021 | 2.64208 | 58.5 | 2149 | GCF_000172135.1_ASM17213v1_genomic.fna |
| *Bifidobacterium dentium* ATCC 27679 | ATCC 27679 | SAMN00210789 | 2.64578 | 58.4 | 2210 | GCF_000146775.1_ASM14677v1_genomic.fna |
| *Bifidobacterium dentium* Bd1 | Bd1 | SAMN02603177 | 2.63637 | 58.5 | 2137 | GCF_000024445.1_ASM2444v1_genomic.fna |
| *Bifidobacterium dentium* JCM 1195 = DSM 20436 | DSM 20436 | SAMN02442045 | 2.61478 | 58.5 | 2128 | GCF_000771725.1_DSM-20436_genomic.fna |
| ***Bifidobacterium dentium* JCM 1195 = DSM 20436** | **JCM 1195** | **SAMD00061043** | **2.63567** | **58.5** | **2140** | **GCF_001042595.1_ASM104259v1_genomic.fna** |
| *Bifidobacterium dentium* JCM 1195 = DSM 20436 | DSM 20436 | SAMN05192536 | 2.66807 | 58.6 | 2152 | GCF_900105745.1_IMG-taxon_2634166294_annotated_assembly_genomic.fna |
| *Bifidobacterium dentium* JCVIHMP022 | JCVIHMP022 | SAMN00115044 | 2.63658 | 58.4 | 2089 | GCF_000149165.1_ASM14916v1_genomic.fna |
| *Bifidobacterium gallicum* DSM 20093 = LMG 11596 | DSM 20093 | SAMN02299421 | 2.0198 | 57.5 | 1491 | GCF_000173375.1_ASM17337v1_genomic.fna |
| *Bifidobacterium gallicum* DSM 20093 = LMG 11596 | LMG 11596 | SAMN02673434 | 2.00459 | 57.6 | 1487 | GCF_000741205.1_Bifgalcum_genomic.fna |
| *Bifidobacterium gallicum* DSM 20093 = LMG 11596 | DSM 20093 | SAMN02442014 | 1.98985 | 57.6 | 1476 | GCF_000771165.1_DSM-20093_genomic.fna |
| *Bifidobacterium gallinarum* | UBA1815 | SAMN06453770 | 1.63265 | 63.4 | - | GCA_002314335.1_ASM231433v1_genomic.fna |
| *Bifidobacterium gallinarum* | LMG 11586 | SAMN02673435 | 2.16084 | 64.2 | 1651 | GCF_000741215.1_Bifgalrum_genomic.fna |
| *Bifidobacterium gallinarum* | CACC 514 | SAMN10782522 | 2.41446 | 63.9011 | 1957 | GCF_004135085.1_ASM413508v1_genomic.fna |
| *Bifidobacterium gallinarum* DSM 20670 | DSM 20670 | SAMN02442031 | 2.12857 | 64.2 | 1648 | GCF_000771505.1_DSM-20670_genomic.fna |

| | | | | | | |
|---|---|---|---|---|---|---|
| *Bifidobacterium indicum* | ESL0197 | SAMN08297247 | 1.71524 | 60.6 | 1314 | GCF_003202665.1_ASM320266v1_genomic.fna |
| *Bifidobacterium indicum* LMG 11587 = DSM 20214 | LMG 11587 | SAMN05771123 | 1.73455 | 60.5 | 1327 | GCF_000706765.1_ASM70676v1_genomic.fna |
| *Bifidobacterium indicum* LMG 11587 = DSM 20214 | DSM 20214 | SAMN02442022 | 1.72396 | 60.5 | 1332 | GCF_000771325.1_DSM-20214_genomic.fna |
| *Bifidobacterium kashiwanohense* JCM 15439 = DSM 21854 | DSM 21854 | SAMN02673436 | 2.30796 | 56.2 | 1739 | GCF_000741605.1_Bifkas_genomic.fna |
| *Bifidobacterium kashiwanohense* JCM 15439 = DSM 21854 | DSM 21854 | SAMN02442033 | 2.32346 | 56.3 | 1825 | GCF_000771545.1_DSM-21854_genomic.fna |
| *Bifidobacterium kashiwanohense* JCM 15439 = DSM 21854 | JCM 15439 | SAMD00061044 | 2.33723 | 56.3 | 1840 | GCF_001042615.1_ASM104261v1_genomic.fna |
| *Bifidobacterium kashiwanohense* PV20-2 | PV20-2 | SAMN03253090 | 2.37098 | 56.1 | 1876 | GCF_000800455.1_ASM80045v1_genomic.fna |
| *Bifidobacterium lemurum* | DSM 28807 | SAMD00065376 | 2.91202 | 62.6 | 2221 | GCF_001895165.1_ASM189516v1_genomic.fna |
| *Bifidobacterium lemurum* | DSM 28807 | SAMN06341149 | 2.94429 | 62.6 | 2231 | GCF_002259665.1_ASM225966v1_genomic.fna |
| *Bifidobacterium longum* | 121.2 | SAMN04497913 | 1.87256 | 60.3 | 1453 | GCA_001576955.1_ASM157695v1_genomic.fna |
| *Bifidobacterium longum* | UBA2088 | SAMN06457477 | 1.87849 | 59.2 | - | GCA_002331305.1_ASM233130v1_genomic.fna |
| *Bifidobacterium longum* | UBA9129 | SAMN08020367 | 1.87394 | 57.4 | 1920 | GCA_003531035.1_ASM353103v1_genomic.fna |
| *Bifidobacterium longum* | 12_1_47BFAA | SAMN02463822 | 2.40599 | 60.1 | 1966 | GCF_000185665.1_ASM18566v1_genomic.fna |
| *Bifidobacterium longum* | BXY01 | SAMN02843216 | 2.4806 | 59.8 | 1961 | GCF_000730205.1_ASM73020v1_genomic.fna |
| *Bifidobacterium longum* | 105-A | SAMD00019943 | 2.29014 | 60.1 | 1823 | GCF_000829295.1_ASM82929v1_genomic.fna |
| *Bifidobacterium longum* | BG7 | SAMN03271682 | 2.45576 | 60.0068 | 1961 | GCF_001293145.1_ASM129314v1_genomic.fna |
| *Bifidobacterium longum* | CMW7750 | SAMN03842222 | 2.37208 | 60 | 1903 | GCF_001546275.1_ASM154627v1_genomic.fna |
| *Bifidobacterium longum* | 379 | SAMN04155602 | 2.38762 | 60.2 | 1939 | GCF_001595465.1_ASM159546v1_genomic.fna |
| *Bifidobacterium longum* | 35624 | SAMN04254466 | 2.26406 | 60 | 1796 | GCF_001719085.1_ASM171908v1_genomic.fna |
| *Bifidobacterium longum* | 1898B | SAMN06621716 | 2.47439 | 59.9 | 1990 | GCF_002075875.1_Bbif1898B_genomic.fna |
| *Bifidobacterium longum* | 1897B | SAMN06621715 | 2.45361 | 59.9 | 1950 | GCF_002075935.1_Bbif1897B_genomic.fna |
| *Bifidobacterium longum* | 1890B | SAMN06621710 | 2.34167 | 59.9 | 1798 | GCF_002076015.1_Bbif1890B_genomic.fna |
| *Bifidobacterium longum* | 1886B | SAMN06621706 | 2.47375 | 60.2 | 2075 | GCF_002076095.1_Bbif1886B_genomic.fna |
| *Bifidobacterium longum* | Indica | SAMN07503177 | 2.37423 | 60 | 1936 | GCF_002276185.1_ASM227618v1_genomic.fna |

| | | | | | | |
|---|---|---|---|---|---|---|
| *Bifidobacterium longum* | DPC6317 | SAMN07958363 | 2.44863 | 60.2 | 1944 | GCF_002832945.1_ASM283294v1_genomic.fna |
| *Bifidobacterium longum* | APC1504 | SAMN07958361 | 2.31029 | 60.2 | 1869 | GCF_002832955.1_ASM283295v1_genomic.fna |
| *Bifidobacterium longum* | APC1503 | SAMN07958360 | 2.5627 | 59.7 | 2132 | GCF_002832985.1_ASM283298v1_genomic.fna |
| *Bifidobacterium longum* | DPC6321 | SAMN07958365 | 2.38236 | 59.9 | 1920 | GCF_002832995.1_ASM283299v1_genomic.fna |
| *Bifidobacterium longum* | DPC6316 | SAMN07958362 | 2.39397 | 60.4 | 1926 | GCF_002833015.1_ASM283301v1_genomic.fna |
| *Bifidobacterium longum* | APC1482 | SAMN07958359 | 2.33744 | 60.2 | 1866 | GCF_002833035.1_ASM283303v1_genomic.fna |
| *Bifidobacterium longum* | APC1478 | SAMN07958357 | 2.22335 | 59.8 | 1744 | GCF_002833055.1_ASM283305v1_genomic.fna |
| *Bifidobacterium longum* | APC1480 | SAMN07958358 | 2.47775 | 59.9 | 2045 | GCF_002833065.1_ASM283306v1_genomic.fna |
| *Bifidobacterium longum* | APC1477 | SAMN07958356 | 2.22881 | 59.8 | 1743 | GCF_002833075.1_ASM283307v1_genomic.fna |
| *Bifidobacterium longum* | APC1472 | SAMN07958353 | 2.36404 | 60.2 | 1875 | GCF_002833115.1_ASM283311v1_genomic.fna |
| *Bifidobacterium longum* | APC1473 | SAMN07958354 | 2.31707 | 59.8 | 1840 | GCF_002833125.1_ASM283312v1_genomic.fna |
| *Bifidobacterium longum* | APC1468 | SAMN07958352 | 2.39516 | 60.2 | 1989 | GCF_002833135.1_ASM283313v1_genomic.fna |
| *Bifidobacterium longum* | APC1465 | SAMN07958350 | 2.45221 | 59.7 | 2012 | GCF_002833175.1_ASM283317v1_genomic.fna |
| *Bifidobacterium longum* | APC1464 | SAMN07958349 | 2.34652 | 60.1 | 1906 | GCF_002833185.1_ASM283318v1_genomic.fna |
| *Bifidobacterium longum* | APC1462 | SAMN07958348 | 2.41778 | 60.3 | 1991 | GCF_002833205.1_ASM283320v1_genomic.fna |
| *Bifidobacterium longum* | DPC6323 | SAMN07958366 | 2.39696 | 60.2 | 1936 | GCF_002833215.1_ASM283321v1_genomic.fna |
| *Bifidobacterium longum* | DPC6320 | SAMN07958364 | 2.33037 | 59.9 | 1832 | GCF_002833255.1_ASM283325v1_genomic.fna |
| *Bifidobacterium longum* | APC1476 | SAMN07958355 | 2.53254 | 60 | 2119 | GCF_002833265.1_ASM283326v1_genomic.fna |
| *Bifidobacterium longum* | APC1466 | SAMN07958351 | 2.41998 | 59.8 | 2003 | GCF_002833285.1_ASM283328v1_genomic.fna |
| *Bifidobacterium longum* | APC1461 | SAMN07958347 | 2.41899 | 60 | 1888 | GCF_002833315.1_ASM283331v1_genomic.fna |
| *Bifidobacterium longum* | UMB0788 | SAMN08193649 | 2.45493 | 60.2 | 2042 | GCF_002861445.1_ASM286144v1_genomic.fna |
| *Bifidobacterium longum* | DS9_3 | SAMN06464100 | 2.39717 | 59.9 | 1980 | GCF_003094635.1_ASM309463v1_genomic.fna |
| *Bifidobacterium longum* | DS15_3 | SAMN06464097 | 2.39818 | 59.9 | 1981 | GCF_003094855.1_ASM309485v1_genomic.fna |
| *Bifidobacterium longum* | DS18_3 | SAMN06464098 | 2.44826 | 59.7 | 2022 | GCF_003094935.1_ASM309493v1_genomic.fna |
| *Bifidobacterium longum* | DS1_3 | SAMN06464096 | 2.41728 | 60 | 1979 | GCF_003094955.1_ASM309495v1_genomic.fna |
| *Bifidobacterium longum* | DS7_3 | SAMN06464099 | 2.23729 | 60 | 1789 | GCF_003094975.1_ASM309497v1_genomic.fna |
| *Bifidobacterium longum* | UMA026 | SAMN08102330 | 2.39256 | 60.1 | 1904 | GCF_003130775.1_ASM313077v1_genomic.fna |
| *Bifidobacterium longum* | N3A01 | SAMN07189849 | 2.30522 | 59.9 | 1733 | GCF_003369995.1_ASM336999v1_genomic.fna |
| *Bifidobacterium longum* | N6D05 | SAMN07189852 | 2.72332 | 59.7 | 2209 | GCF_003370005.1_ASM337000v1_genomic.fna |

| | | | | | | |
|---|---|---|---|---|---|---|
| *Bifidobacterium longum* | N2G10 | SAMN07189848 | 2.27838 | 59.8 | 1891 | GCF_003370025.1_ASM337002v1_genomic.fna |
| *Bifidobacterium longum* | N2F05 | SAMN07189847 | 2.33342 | 59.9 | 1787 | GCF_003370035.1_ASM337003v1_genomic.fna |
| *Bifidobacterium longum* | N2E12 | SAMN07189846 | 2.34466 | 60 | 1745 | GCF_003370065.1_ASM337006v1_genomic.fna |
| *Bifidobacterium longum* | W35-1 | SAMN07189845 | 2.29837 | 59.9 | 1761 | GCF_003370095.1_ASM337009v1_genomic.fna |
| *Bifidobacterium longum* | N5E04 | SAMN07189851 | 2.45599 | 59.3 | 1701 | GCF_003370205.1_ASM337020v1_genomic.fna |
| *Bifidobacterium longum* | N3E01-2 | SAMN07189850 | 2.34278 | 59.7 | 1716 | GCF_003370225.1_ASM337022v1_genomic.fna |
| *Bifidobacterium longum* | 239-2 | SAMN07189844 | 2.27516 | 59.7 | 1677 | GCF_003370255.1_ASM337025v1_genomic.fna |
| *Bifidobacterium longum* | TM04-17 | SAMN09736971 | 2.4148 | 60 | 2013 | GCF_003436505.1_ASM343650v1_genomic.fna |
| *Bifidobacterium longum* | TM02-7 | SAMN09736966 | 2.39325 | 60.3 | 1927 | GCF_003436595.1_ASM343659v1_genomic.fna |
| *Bifidobacterium longum* | TF07-39 | SAMN09736908 | 2.45182 | 60.1 | 2017 | GCF_003437095.1_ASM343709v1_genomic.fna |
| *Bifidobacterium longum* | TF07-34 | SAMN09736907 | 2.3738 | 60.1 | 1910 | GCF_003437105.1_ASM343710v1_genomic.fna |
| *Bifidobacterium longum* | TF07-31 | SAMN09736906 | 2.44332 | 60 | 2000 | GCF_003437135.1_ASM343713v1_genomic.fna |
| *Bifidobacterium longum* | TM01-1 | SAMN09736962 | 2.35075 | 60.3 | 1909 | GCF_003437455.1_ASM343745v1_genomic.fna |
| *Bifidobacterium longum* | TF08-4AC | SAMN09736919 | 2.4296 | 60.1 | 1954 | GCF_003437695.1_ASM343769v1_genomic.fna |
| *Bifidobacterium longum* | TF06-45A | SAMN09736897 | 2.52562 | 59.6 | 2162 | GCF_003437715.1_ASM343771v1_genomic.fna |
| *Bifidobacterium longum* | TF01-22 | SAMN09736866 | 2.38449 | 60.2 | 1928 | GCF_003437995.1_ASM343799v1_genomic.fna |
| *Bifidobacterium longum* | OM05-2BH | SAMN09736802 | 2.43685 | 60.1 | 2042 | GCF_003438385.1_ASM343838v1_genomic.fna |
| *Bifidobacterium longum* | TF06-12AC | SAMN09736888 | 2.52763 | 59.6 | 2164 | GCF_003438485.1_ASM343848v1_genomic.fna |
| *Bifidobacterium longum* | AF27-1BH | SAMN09734529 | 2.33708 | 60.1 | 1892 | GCF_003457975.1_ASM345797v1_genomic.fna |
| *Bifidobacterium longum* | AF26-10 | SAMN09734508 | 2.34746 | 60.2 | 1913 | GCF_003458335.1_ASM345833v1_genomic.fna |
| *Bifidobacterium longum* | OF01-16 | SAMN09736665 | 2.2483 | 60 | 1785 | GCF_003463285.1_ASM346328v1_genomic.fna |
| *Bifidobacterium longum* | AF14-34 | SAMN09734258 | 2.40908 | 60.1 | 1963 | GCF_003464575.1_ASM346457v1_genomic.fna |
| *Bifidobacterium longum* | AF14-22 | SAMN09734253 | 2.40721 | 60.1 | 1959 | GCF_003464685.1_ASM346468v1_genomic.fna |
| *Bifidobacterium longum* | AF13-41 | SAMN09734240 | 2.32031 | 60.4 | 1868 | GCF_003464805.1_ASM346480v1_genomic.fna |
| *Bifidobacterium longum* | AF11-41 | SAMN09734211 | 2.38362 | 60.3 | 1944 | GCF_003465095.1_ASM346509v1_genomic.fna |
| *Bifidobacterium longum* | AF13-34 | SAMN09734235 | 2.31949 | 60.4 | 1873 | GCF_003465365.1_ASM346536v1_genomic.fna |
| *Bifidobacterium longum* | AF11-12 | SAMN09734206 | 2.68534 | 60.3 | 2252 | GCF_003465455.1_ASM346545v1_genomic.fna |
| *Bifidobacterium longum* | AF08-2 | SAMN09734194 | 2.54012 | 60.2 | 2121 | GCF_003465545.1_ASM346554v1_genomic.fna |
| *Bifidobacterium longum* | AF05-2 | SAMN09734186 | 2.42691 | 60.4 | 2015 | GCF_003465635.1_ASM346563v1_genomic.fna |

| | | | | | | |
|---|---|---|---|---|---|---|
| *Bifidobacterium longum* | AF05-16 | SAMN09734185 | 2.42575 | 60.4 | 2017 | GCF_003465665.1_ASM346566v1_genomic.fna |
| *Bifidobacterium longum* | AF04-13 | SAMN09734175 | 2.37602 | 60.1 | 1913 | GCF_003465745.1_ASM346574v1_genomic.fna |
| *Bifidobacterium longum* | AF03-27 | SAMN09734172 | 2.44611 | 60.2 | 1939 | GCF_003465785.1_ASM346578v1_genomic.fna |
| *Bifidobacterium longum* | AF03-20 | SAMN09734170 | 2.44578 | 60.2 | 1935 | GCF_003466045.1_ASM346604v1_genomic.fna |
| *Bifidobacterium longum* | AF03-10 | SAMN09734168 | 2.44408 | 60.2 | 1936 | GCF_003466075.1_ASM346607v1_genomic.fna |
| *Bifidobacterium longum* | TM06-1 | SAMN09736986 | 2.40404 | 60.2 | 1941 | GCF_003466425.1_ASM346642v1_genomic.fna |
| *Bifidobacterium longum* | TM05-14 | SAMN09736978 | 2.50681 | 59.9 | 2090 | GCF_003466505.1_ASM346650v1_genomic.fna |
| *Bifidobacterium longum* | TM04-48B | SAMN09736975 | 2.37324 | 60 | 1967 | GCF_003466545.1_ASM346654v1_genomic.fna |
| *Bifidobacterium longum* | AM39-8AC | SAMN09736517 | 2.34569 | 60.1 | 1905 | GCF_003467395.1_ASM346739v1_genomic.fna |
| *Bifidobacterium longum* | AM34-3 | SAMN09736466 | 2.37749 | 60.1 | 1905 | GCF_003467905.1_ASM346790v1_genomic.fna |
| *Bifidobacterium longum* | AM42-13AT | SAMN09736542 | 2.22637 | 60.1 | 1784 | GCF_003468005.1_ASM346800v1_genomic.fna |
| *Bifidobacterium longum* | AM39-10AC | SAMN09736509 | 2.23371 | 60 | 1773 | GCF_003468265.1_ASM346826v1_genomic.fna |
| *Bifidobacterium longum* | AM31-13LB | SAMN09736426 | 2.43452 | 60.2 | 1998 | GCF_003468805.1_ASM346880v1_genomic.fna |
| *Bifidobacterium longum* | AM30-9LB | SAMN09736422 | 2.29747 | 60.1 | 1901 | GCF_003468825.1_ASM346882v1_genomic.fna |
| *Bifidobacterium longum* | AM21-20 | SAMN09734892 | 2.33249 | 60 | 1862 | GCF_003471245.1_ASM347124v1_genomic.fna |
| *Bifidobacterium longum* | AM20-3 | SAMN09734882 | 2.2981 | 60 | 1808 | GCF_003471345.1_ASM347134v1_genomic.fna |
| *Bifidobacterium longum* | AM20-39 | SAMN09734884 | 2.29653 | 60 | 1815 | GCF_003471355.1_ASM347135v1_genomic.fna |
| *Bifidobacterium longum* | AM20-19AC | SAMN09734881 | 2.29489 | 60 | 1807 | GCF_003471385.1_ASM347138v1_genomic.fna |
| *Bifidobacterium longum* | AM16-2 | SAMN09734826 | 2.28838 | 60 | 1861 | GCF_003471915.1_ASM347191v1_genomic.fna |
| *Bifidobacterium longum* | AM12-16 | SAMN09734785 | 2.30797 | 60 | 1832 | GCF_003472305.1_ASM347230v1_genomic.fna |
| *Bifidobacterium longum* | AM11-2 | SAMN09734779 | 2.32334 | 60.2 | 1881 | GCF_003472405.1_ASM347240v1_genomic.fna |
| *Bifidobacterium longum* | AM10-15B | SAMN09734765 | 2.41411 | 60.2 | 1973 | GCF_003472605.1_ASM347260v1_genomic.fna |
| *Bifidobacterium longum* | AM11-5 | SAMN09734782 | 2.35711 | 60.1 | 1911 | GCF_003473205.1_ASM347320v1_genomic.fna |
| *Bifidobacterium longum* | AF36-1 | SAMN09734632 | 2.37886 | 60.1 | 1944 | GCF_003474905.1_ASM347490v1_genomic.fna |
| *Bifidobacterium longum* | AF35-13AC | SAMN09734620 | 2.3945 | 60.1 | 1940 | GCF_003475025.1_ASM347502v1_genomic.fna |
| *Bifidobacterium longum* | AF34-9AC | SAMN09734618 | 2.4028 | 60 | 1936 | GCF_003475055.1_ASM347505v1_genomic.fna |
| *Bifidobacterium longum* | AF30-12 | SAMN09734565 | 2.26564 | 59.9 | 1811 | GCF_003475835.1_ASM347583v1_genomic.fna |
| *Bifidobacterium longum* | AF30-11 | SAMN09734564 | 2.2659 | 59.9 | 1811 | GCF_003475865.1_ASM347586v1_genomic.fna |
| *Bifidobacterium longum* | ICIS-505 | SAMN06606733 | 2.44884 | 59.8086 | 2001 | GCF_003790375.1_ASM379037v1_genomic.fna |

| | | | | | | |
|---|---|---|---|---|---|---|
| *Bifidobacterium longum* | BB-79 | SAMN08120363 | 2.20609 | 59.9 | 1797 | GCF_004125375.1_ASM412537v1_genomic.fna |
| *Bifidobacterium longum* | bk_0021 | SAMN10239587 | 2.32045 | 59.9 | 1918 | GCF_004167725.1_ASM416772v1_genomic.fna |
| *Bifidobacterium longum* | ZJ1 | SAMN11513273 | 2.41467 | - | - | GCF_005406285.1_ASM540628v1_genomic.fna |
| *Bifidobacterium longum* | BSD2780061688st2_H1 | SAMN10863273 | 2.2799 | 59.9 | - | GCF_005844115.1_ASM584411v1_genomic.fna |
| *Bifidobacterium longum* | 1001271st1_B4 | SAMN10863261 | 2.44231 | 60.2 | - | GCF_005844345.1_ASM584434v1_genomic.fna |
| *Bifidobacterium longum* | 1001175st1_G10 | SAMN10863239 | 2.33608 | 59.9 | - | GCF_005844715.1_ASM584471v1_genomic.fna |
| *Bifidobacterium longum* | Su859 | SAMN04489749 | 2.39298 | 60 | 1872 | GCF_900103055.1_IMG-taxon_2636416037_annotated_assembly_genomic.fna |
| *Bifidobacterium longum* | DSM 20219 | SAMN04489748 | 2.44902 | 60.3 | 1945 | GCF_900104835.1_IMG-taxon_2634166334_annotated_assembly_genomic.fna |
| *Bifidobacterium longum* | Bifido_09 | SAMEA51821668 | 2.66124 | 59.9 | 2208 | GCF_900157055.1_Bifido_09_v1_genomic.fna |
| *Bifidobacterium longum* | PC1 | SAMEA51825418 | 2.79457 | 59.8 | 2389 | GCF_900157065.1_PC1_v1_genomic.fna |
| *Bifidobacterium longum* | Bifido_S1 | SAMEA51824668 | 2.81203 | 59.7 | 2410 | GCF_900157075.1_Bifido_S1_v1_genomic.fna |
| *Bifidobacterium longum* | Bifido_04 | SAMEA51817918 | 2.57939 | 59.7 | 2108 | GCF_900157085.1_Bifido_04_v1_genomic.fna |
| *Bifidobacterium longum* | Bifido_01 | SAMEA51815668 | 2.33463 | 59.9 | 1907 | GCF_900157095.1_Bifido_01_v1_genomic.fna |
| *Bifidobacterium longum* | Bifido_05 | SAMEA51818668 | 2.33474 | 59.9 | 1868 | GCF_900157115.1_Bifido_05_v1_genomic.fna |
| *Bifidobacterium longum* | Bifido_03 | SAMEA51817168 | 2.41363 | 60.1 | 1960 | GCF_900157145.1_Bifido_03_v1_genomic.fna |
| *Bifidobacterium longum* | Bifido_06 | SAMEA51819418 | 2.42182 | 60 | 1987 | GCF_900157155.1_Bifido_06_v1_genomic.fna |
| *Bifidobacterium longum* | Bifido_12 | SAMEA51823918 | 2.07288 | 60.5 | 1619 | GCF_900157165.1_Bifido_12_v1_genomic.fna |
| *Bifidobacterium longum* | PC4 | SAMEA51826168 | 2.78899 | 59.8 | 2405 | GCF_900157185.1_PC4_v1_genomic.fna |
| *Bifidobacterium longum* | Bifido_02 | SAMEA51816418 | 2.33429 | 60.1 | 1844 | GCF_900157195.1_Bifido_02_v1_genomic.fna |
| *Bifidobacterium longum* | 1 | SAMEA4827164 | 2.20609 | 59.9 | 1800 | GCF_900519135.1_BB-79_genomic.fna |
| *Bifidobacterium longum* | NCTC11818 | SAMEA4412687 | 2.38516 | 60.3 | 1915 | GCF_900637335.1_50618_H02_genomic.fna |
| *Bifidobacterium longum* AGR2137 | AGR2137 | SAMN02441228 | 2.27038 | 59.9 | 1779 | GCF_000421385.1_ASM42138v1_genomic.fna |
| *Bifidobacterium longum* D2957 | D2957 | SAMN02472064 | 2.33023 | 60.4 | 1830 | GCF_000478525.1_blongD2957_genomic.fna |
| *Bifidobacterium longum* DJO10A | DJO10A | SAMN02603512 | 2.38953 | 60.1182 | 1946 | GCF_000008945.1_ASM894v1_genomic.fna |
| *Bifidobacterium longum* DJO10A | DJO10A | SAMN02441414 | 2.37528 | 59.9 | 1815 | GCF_000166895.2_ASM16689v2_genomic.fna |
| *Bifidobacterium longum* E18 | E18 | SAMN02471972 | 2.37297 | 60 | 1942 | GCF_000497735.1_BLONGv1.0_genomic.fna |
| ***Bifidobacterium longum* NCC2705** | **NCC2705** | **SAMN02603675** | **2.26027** | **60.1075** | **1728** | **GCF_000007525.1_ASM752v1_genomic.fna** |

| | | | | | | |
|---|---|---|---|---|---|---|
| *Bifidobacterium longum* subsp. *infantis* | BIC1206122787 | SAMEA2747183 | 2.78904 | 59.8 | 2408 | GCF_000825005.1_BIC1206122787.V1_genomic.fna |
| *Bifidobacterium longum* subsp. *infantis* | BIC1307292462 | SAMEA2747184 | 2.87962 | 59.9 | 2453 | GCF_000825025.1_BIC1307292462.V1_genomic.fna |
| *Bifidobacterium longum* subsp. *infantis* | BIC1401111250 | SAMEA2747185 | 2.79389 | 59.8 | 2409 | GCF_000825045.1_BIC1401111250.V1_genomic.fna |
| *Bifidobacterium longum* subsp. *infantis* | BIC1401212621a | SAMEA2747186 | 2.79157 | 59.8 | 2413 | GCF_000825065.1_BIC1401212621a.V1_genomic.fna |
| *Bifidobacterium longum* subsp. *infantis* | BIC1401212621b | SAMEA2747187 | 2.82188 | 59.9 | 2429 | GCF_000825085.1_BIC1401212621b.V1_genomic.fna |
| *Bifidobacterium longum* subsp. *infantis* | BIB1401242951 | SAMEA2747188 | 2.78753 | 59.8 | 2407 | GCF_000825105.1_BIB1401242951.V1_genomic.fna |
| *Bifidobacterium longum* subsp. *infantis* | BIB1401272845a | SAMEA2747189 | 2.79152 | 59.8 | 2404 | GCF_000825125.1_BIB1401272845a.V1_genomic.fna |
| *Bifidobacterium longum* subsp. *infantis* | BIB1401272845b | SAMEA2747190 | 2.78684 | 59.8 | 2405 | GCF_000825145.1_BIB1401272845b.V1_genomic.fna |
| *Bifidobacterium longum* subsp. *infantis* | CECT 7210 | SAMEA3158508 | 2.4677 | 59.9 | 2033 | GCF_001051015.2_ASM105101v2_genomic.fna |
| *Bifidobacterium longum* subsp. *infantis* | BT1 | SAMN03271683 | 2.57811 | 59.4 | 2035 | GCF_001281305.1_ASM128130v1_genomic.fna |
| *Bifidobacterium longum* subsp. *infantis* | IN-07 | SAMD00047616 | 2.74983 | 60 | 2170 | GCF_001686105.1_ASM168610v1_genomic.fna |
| *Bifidobacterium longum* subsp. *infantis* | IN-F29 | SAMD00047617 | 2.64362 | 59.9 | 2106 | GCF_001686125.1_ASM168612v1_genomic.fna |
| *Bifidobacterium longum* subsp. *infantis* | TPY12-1 | SAMN05578879 | 2.64013 | 59.7 | 2144 | GCF_001870755.1_ASM187075v1_genomic.fna |
| *Bifidobacterium longum* subsp. *infantis* | 1888B | SAMN06621708 | 2.57973 | 59.4 | 2038 | GCF_002076025.1_Bbif1888B_genomic.fna |
| *Bifidobacterium longum* subsp. *infantis* | CECT 7210 | SAMEA3158508 | 2.4677 | 59.9 | 2033 | GCF_002900845.1_ASM290084v1_genomic.fna |

| | | | | | | |
|---|---|---|---|---|---|---|
| *Bifidobacterium longum* subsp. *infantis* | UBBI-01 | SAMN11370925 | 2.73164 | 59.4 | 2259 | GCF_004803425.1_ASM480342v1_genomic.fna |
| *Bifidobacterium longum* subsp. *infantis* | Bi-26 | SAMN10380491 | 2.56944 | - | - | GCF_004919065.1_ASM491906v1_genomic.fna |
| *Bifidobacterium longum* subsp. *infantis* | NCTC13219 | SAMEA104318167 | 2.60259 | 60 | 2224 | GCF_900445755.1_59024_E01_genomic.fna |
| *Bifidobacterium longum* subsp. *infantis* | NCTC11817 | SAMEA44530168 | 2.83275 | 59.9 | 2438 | GCF_900637215.1_49888_B01_genomic.fna |
| *Bifidobacterium longum* subsp. *infantis* 157F | 157F | SAMD00060953 | 2.40883 | 60.111 | 1994 | GCF_000196575.1_ASM19657v1_genomic.fna |
| *Bifidobacterium longum* subsp. *infantis* ATCC 15697 = JCM 1222 = DSM 20088 | ATCC 15697 | SAMN02598380 | 2.83275 | 59.9 | 2438 | GCF_000020425.1_ASM2042v1_genomic.fna |
| *Bifidobacterium longum* subsp. *infantis* ATCC 15697 = JCM 1222 = DSM 20088 | JCM 1222 | SAMD00060952 | 2.82896 | 59.9 | 2443 | GCF_000269965.1_ASM26996v1_genomic.fna |
| *Bifidobacterium longum* subsp. *infantis* ATCC 15697 = JCM 1222 = DSM 20088 | DSM 20088 | SAMN02442011 | 2.78032 | 59.8 | 2386 | GCF_000771105.1_DSM-20088_genomic.fna |
| *Bifidobacterium longum* subsp. *infantis* CCUG 52486 | CCUG 52486 | SAMN02463677 | 2.48085 | 60 | 2067 | GCF_000155415.1_ASM15541v1_genomic.fna |
| *Bifidobacterium longum* subsp. *infantis* EK3 | EK3 | SAMN02862995 | 2.56481 | 59.4 | 2081 | GCF_000730125.1_ASM73012v1_genomic.fna |
| *Bifidobacterium longum* subsp. *longum* | LMG 13197 | SAMN02673437 | 2.3847 | 60.3 | 1852 | GCF_000741245.1_Biflon_sub.lon_genomic.fna |
| *Bifidobacterium longum* subsp. *longum* | VMKB44 | SAMN03105207 | 2.50193 | 60.3 | 2074 | GCF_000786175.1_ASM78617v1_genomic.fna |
| *Bifidobacterium longum* subsp. *longum* | BLOI2 | SAMN03775040 | 2.41759 | 60 | 1955 | GCF_001275745.1_assBLOI2_genomic.fna |

| | | | | | | |
|---|---|---|---|---|---|---|
| *Bifidobacterium longum* subsp. *longum* | NCIMB8809 | SAMN03785818 | 2.34099 | 60.1 | 1855 | GCF_001446255.1_ASM144625v1_genomic.fna |
| *Bifidobacterium longum* subsp. *longum* | CCUG30698 | SAMN03785819 | 2.458 | 60.2 | 2005 | GCF_001446275.1_ASM144627v1_genomic.fna |
| *Bifidobacterium longum* subsp. *longum* | 9 | SAMN04129541 | 2.23377 | 60 | 1789 | GCF_001447955.1_ASM144795v1_genomic.fna |
| *Bifidobacterium longum* subsp. *longum* | 7 | SAMN04129533 | 2.23558 | 60 | 1789 | GCF_001447975.1_ASM144797v1_genomic.fna |
| *Bifidobacterium longum* subsp. *longum* | MC-42 | SAMN04263942 | 2.28825 | 59.8 | 1818 | GCF_001516925.1_ASM151692v1_genomic.fna |
| *Bifidobacterium longum* subsp. *longum* | LO-06 | SAMD00047618 | 2.43747 | 60 | 1964 | GCF_001686145.1_ASM168614v1_genomic.fna |
| *Bifidobacterium longum* subsp. *longum* | LO-10 | SAMD00047619 | 2.54024 | 60.3 | 2052 | GCF_001686165.1_ASM168616v1_genomic.fna |
| *Bifidobacterium longum* subsp. *longum* | LO-21 | SAMD00047620 | 2.65603 | 60.1 | 2054 | GCF_001686185.1_ASM168618v1_genomic.fna |
| *Bifidobacterium longum* subsp. *longum* | LO-C29 | SAMD00047621 | 2.48387 | 60 | 1963 | GCF_001686205.1_ASM168620v1_genomic.fna |
| *Bifidobacterium longum* subsp. *longum* | LO-K29a | SAMD00047622 | 2.44918 | 60 | 1954 | GCF_001686225.1_ASM168622v1_genomic.fna |
| *Bifidobacterium longum* subsp. *longum* | LO-K29b | SAMD00047623 | 2.37271 | 60.1 | 1891 | GCF_001686245.1_ASM168624v1_genomic.fna |
| *Bifidobacterium longum* subsp. *longum* | AH1206 | SAMN04576213 | 2.42129 | 60.2 | 2007 | GCF_001725985.1_ASM172598v1_genomic.fna |
| *Bifidobacterium longum* subsp. *longum* | 296B | SAMN05916052 | 2.25318 | 59.9 | 1850 | GCF_001892965.1_ASM189296v1_genomic.fna |
| *Bifidobacterium longum* subsp. *longum* | W11 | SAMN06109230 | 2.32998 | 59.9 | 1844 | GCF_001940535.1_BlonW11v1_genomic.fna |
| *Bifidobacterium longum* subsp. *longum* | DS32_3 | SAMN08949007 | 2.23593 | 60.1 | 1791 | GCF_003094995.1_ASM309499v1_genomic.fna |

| | | | | | | |
|---|---|---|---|---|---|---|
| *Bifidobacterium longum* subsp. *longum* | BORI | SAMN09691177 | 2.31102 | 59.9 | 1804 | GCF_003342655.1_ASM334265v1_genomic.fna |
| *Bifidobacterium longum* subsp. *longum* | VKPM Ac-1636 | SAMN10644101 | 2.32174 | 60.2 | 1890 | GCF_003990235.1_ASM399023v1_genomic.fna |
| *Bifidobacterium longum* subsp. *longum* | C11A10B | SAMN09355370 | 2.49303 | 60.1 | 2069 | GCF_004324325.1_ASM432432v1_genomic.fna |
| *Bifidobacterium longum* subsp. *longum* | MCC10114 | SAMN06368664 | 2.45459 | 59.8 | 2033 | GCF_004332625.1_ASM433262v1_genomic.fna |
| *Bifidobacterium longum* subsp. *longum* | MCC10113 | SAMN06368663 | 2.46411 | 60 | 2070 | GCF_004332635.1_ASM433263v1_genomic.fna |
| *Bifidobacterium longum* subsp. *longum* | MCC10115 | SAMN06368665 | 2.43372 | 60.3 | 2059 | GCF_004332645.1_ASM433264v1_genomic.fna |
| *Bifidobacterium longum* subsp. *longum* | MCC10111 | SAMN06368661 | 2.43826 | 60 | 2059 | GCF_004332655.1_ASM433265v1_genomic.fna |
| *Bifidobacterium longum* subsp. *longum* | MCC10108 | SAMN06368660 | 2.41875 | 60.3 | 2005 | GCF_004332665.1_ASM433266v1_genomic.fna |
| *Bifidobacterium longum* subsp. *longum* | MCC10101 | SAMN06368655 | 2.41806 | 60.1 | 1976 | GCF_004332725.1_ASM433272v1_genomic.fna |
| *Bifidobacterium longum* subsp. *longum* | MCC10106 | SAMN06368658 | 2.41503 | 60.1 | 1976 | GCF_004332735.1_ASM433273v1_genomic.fna |
| *Bifidobacterium longum* subsp. *longum* | MCC10099 | SAMN06368653 | 2.34007 | 60.1 | 1916 | GCF_004332745.1_ASM433274v1_genomic.fna |
| *Bifidobacterium longum* subsp. *longum* | MCC10102 | SAMN06368656 | 2.53875 | 60.1 | 2101 | GCF_004332755.1_ASM433275v1_genomic.fna |
| *Bifidobacterium longum* subsp. *longum* | MCC10107 | SAMN06368659 | 2.38475 | 59.8 | 1971 | GCF_004332765.1_ASM433276v1_genomic.fna |
| *Bifidobacterium longum* subsp. *longum* | MCC10097 | SAMN06368651 | 2.28035 | 60 | 1849 | GCF_004332825.1_ASM433282v1_genomic.fna |
| *Bifidobacterium longum* subsp. *longum* | MCC10098 | SAMN06368652 | 2.32667 | 59.9 | 1864 | GCF_004332835.1_ASM433283v1_genomic.fna |

| | | | | | | |
|---|---|---|---|---|---|---|
| *Bifidobacterium longum* subsp. *longum* | MCC10095 | SAMN06368649 | 2.35682 | 60.3 | 1938 | GCF_004332855.1_ASM433285v1_genomic.fna |
| *Bifidobacterium longum* subsp. *longum* | MCC10093 | SAMN06368647 | 2.45894 | 60.2 | 2035 | GCF_004332865.1_ASM433286v1_genomic.fna |
| *Bifidobacterium longum* subsp. *longum* | MCC10094 | SAMN06368648 | 2.50962 | 59.9 | 2109 | GCF_004332895.1_ASM433289v1_genomic.fna |
| *Bifidobacterium longum* subsp. *longum* | MCC10092 | SAMN06368646 | 2.23483 | 59.9 | 1789 | GCF_004332925.1_ASM433292v1_genomic.fna |
| *Bifidobacterium longum* subsp. *longum* | MCC10112 | SAMN06368662 | 2.27757 | 60 | 1848 | GCF_004332935.1_ASM433293v1_genomic.fna |
| *Bifidobacterium longum* subsp. *longum* | MCC10116 | SAMN06368666 | 2.62898 | 60 | 2209 | GCF_004332945.1_ASM433294v1_genomic.fna |
| *Bifidobacterium longum* subsp. *longum* | MCC10100 | SAMN06368654 | 2.518 | 60.1 | 2081 | GCF_004332965.1_ASM433296v1_genomic.fna |
| *Bifidobacterium longum* subsp. *longum* | MCC10103 | SAMN06368657 | 2.39468 | 59.9 | 1976 | GCF_004333005.1_ASM433300v1_genomic.fna |
| *Bifidobacterium longum* subsp. *longum* | MCC10091 | SAMN06368645 | 2.39421 | 60 | 1972 | GCF_004333015.1_ASM433301v1_genomic.fna |
| *Bifidobacterium longum* subsp. *longum* | MCC10090 | SAMN06368644 | 2.34731 | 59.8 | 1931 | GCF_004333035.1_ASM433303v1_genomic.fna |
| *Bifidobacterium longum* subsp. *longum* | MCC10096 | SAMN06368650 | 2.57129 | 59.7 | 2133 | GCF_004333045.1_ASM433304v1_genomic.fna |
| *Bifidobacterium longum* subsp. *longum* | MCC10089 | SAMN06368643 | 2.32481 | 60.3 | 1883 | GCF_004333065.1_ASM433306v1_genomic.fna |
| *Bifidobacterium longum* subsp. *longum* | MCC10087 | SAMN06368642 | 2.30319 | 60 | 1853 | GCF_004333105.1_ASM433310v1_genomic.fna |
| *Bifidobacterium longum* subsp. *longum* | MCC10086 | SAMN06368641 | 2.28382 | 59.8 | 1824 | GCF_004333115.1_ASM433311v1_genomic.fna |
| *Bifidobacterium longum* subsp. *longum* | MCC10084 | SAMN06368639 | 2.26435 | 60.1 | 1836 | GCF_004333125.1_ASM433312v1_genomic.fna |

| | | | | | | |
|---|---|---|---|---|---|---|
| *Bifidobacterium longum* subsp. *longum* | MCC10081 | SAMN06368637 | 2.32346 | 59.9 | 1953 | GCF_004333165.1_ASM433316v1_genomic.fna |
| *Bifidobacterium longum* subsp. *longum* | MCC10080 | SAMN06368636 | 2.52893 | 60.2 | 2049 | GCF_004333175.1_ASM433317v1_genomic.fna |
| *Bifidobacterium longum* subsp. *longum* | MCC10078 | SAMN06368634 | 2.27198 | 59.8 | 1803 | GCF_004333205.1_ASM433320v1_genomic.fna |
| *Bifidobacterium longum* subsp. *longum* | MCC10079 | SAMN06368635 | 2.38272 | 59.9 | 1972 | GCF_004333215.1_ASM433321v1_genomic.fna |
| *Bifidobacterium longum* subsp. *longum* | MCC10076 | SAMN06368632 | 2.56355 | 60.2 | 2172 | GCF_004333235.1_ASM433323v1_genomic.fna |
| *Bifidobacterium longum* subsp. *longum* | MCC10075 | SAMN06368631 | 2.38497 | 60.1 | 1970 | GCF_004333265.1_ASM433326v1_genomic.fna |
| *Bifidobacterium longum* subsp. *longum* | MCC10073 | SAMN06368629 | 2.2843 | 59.7 | 1866 | GCF_004333275.1_ASM433327v1_genomic.fna |
| *Bifidobacterium longum* subsp. *longum* | MCC10071 | SAMN06368627 | 2.28772 | 60 | 1831 | GCF_004333305.1_ASM433330v1_genomic.fna |
| *Bifidobacterium longum* subsp. *longum* | MCC10070 | SAMN06368626 | 2.50822 | 59.6 | 2069 | GCF_004333325.1_ASM433332v1_genomic.fna |
| *Bifidobacterium longum* subsp. *longum* | MCC10069 | SAMN06368625 | 2.36718 | 59.9 | 1925 | GCF_004333335.1_ASM433333v1_genomic.fna |
| *Bifidobacterium longum* subsp. *longum* | MCC10068 | SAMN06368624 | 2.40787 | 59.7 | 1940 | GCF_004333365.1_ASM433336v1_genomic.fna |
| *Bifidobacterium longum* subsp. *longum* | MCC10067 | SAMN06368623 | 2.39437 | 59.6 | 1950 | GCF_004333375.1_ASM433337v1_genomic.fna |
| *Bifidobacterium longum* subsp. *longum* | MCC10066 | SAMN06368622 | 2.29383 | 59.8 | 1889 | GCF_004333385.1_ASM433338v1_genomic.fna |
| *Bifidobacterium longum* subsp. *longum* | MCC10064 | SAMN06368621 | 2.26578 | 60 | 1839 | GCF_004333425.1_ASM433342v1_genomic.fna |
| *Bifidobacterium longum* subsp. *longum* | MCC10062 | SAMN06368620 | 2.32592 | 59.8 | 1890 | GCF_004333445.1_ASM433344v1_genomic.fna |

| | | | | | | |
|---|---|---|---|---|---|---|
| *Bifidobacterium longum* subsp. *longum* | MCC10055 | SAMN06368614 | 2.48603 | 60.1 | 2047 | GCF_004333455.1_ASM433345v1_genomic.fna |
| *Bifidobacterium longum* subsp. *longum* | MCC10060 | SAMN06368619 | 2.31311 | 60.2 | 1877 | GCF_004333465.1_ASM433346v1_genomic.fna |
| *Bifidobacterium longum* subsp. *longum* | MCC10056 | SAMN06368615 | 2.29975 | 60 | 1886 | GCF_004333475.1_ASM433347v1_genomic.fna |
| *Bifidobacterium longum* subsp. *longum* | MCC10053 | SAMN06368612 | 2.42582 | 60.3 | 1993 | GCF_004333515.1_ASM433351v1_genomic.fna |
| *Bifidobacterium longum* subsp. *longum* | MCC10052 | SAMN06368611 | 2.42874 | 60.1 | 1986 | GCF_004333535.1_ASM433353v1_genomic.fna |
| *Bifidobacterium longum* subsp. *longum* | MCC10050 | SAMN06368609 | 2.28037 | 59.8 | 1827 | GCF_004333555.1_ASM433355v1_genomic.fna |
| *Bifidobacterium longum* subsp. *longum* | MCC10048 | SAMN06368608 | 2.45602 | 59.8 | 1993 | GCF_004333565.1_ASM433356v1_genomic.fna |
| *Bifidobacterium longum* subsp. *longum* | MCC10046 | SAMN06368606 | 2.28641 | 59.9 | 1810 | GCF_004333575.1_ASM433357v1_genomic.fna |
| *Bifidobacterium longum* subsp. *longum* | MCC10045 | SAMN06368605 | 2.43778 | 60.3 | 2022 | GCF_004333625.1_ASM433362v1_genomic.fna |
| *Bifidobacterium longum* subsp. *longum* | MCC10043 | SAMN06368603 | 2.62386 | 59.5 | 2152 | GCF_004333635.1_ASM433363v1_genomic.fna |
| *Bifidobacterium longum* subsp. *longum* | MCC10044 | SAMN06368604 | 2.51281 | 60.3 | 2087 | GCF_004333645.1_ASM433364v1_genomic.fna |
| *Bifidobacterium longum* subsp. *longum* | MCC10042 | SAMN06368602 | 2.32056 | 60.1 | 1913 | GCF_004333675.1_ASM433367v1_genomic.fna |
| *Bifidobacterium longum* subsp. *longum* | MCC10041 | SAMN06368601 | 2.37064 | 60.1 | 1942 | GCF_004333695.1_ASM433369v1_genomic.fna |
| *Bifidobacterium longum* subsp. *longum* | MCC10035 | SAMN06368596 | 2.4575 | 59.8 | 2058 | GCF_004333715.1_ASM433371v1_genomic.fna |
| *Bifidobacterium longum* subsp. *longum* | MCC10036 | SAMN06368597 | 2.24998 | 59.9 | 1850 | GCF_004333735.1_ASM433373v1_genomic.fna |

| | | | | | | |
|---|---|---|---|---|---|---|
| *Bifidobacterium longum* subsp. *longum* | MCC10034 | SAMN06368595 | 2.25363 | 60 | 1805 | GCF_004333765.1_ASM433376v1_genomic.fna |
| *Bifidobacterium longum* subsp. *longum* | MCC10033 | SAMN06368594 | 2.39204 | 60 | 1955 | GCF_004333775.1_ASM433377v1_genomic.fna |
| *Bifidobacterium longum* subsp. *longum* | MCC10031 | SAMN06368593 | 2.40115 | 60.1 | 1959 | GCF_004333785.1_ASM433378v1_genomic.fna |
| *Bifidobacterium longum* subsp. *longum* | MCC10029 | SAMN06368591 | 2.3461 | 59.9 | 1915 | GCF_004333795.1_ASM433379v1_genomic.fna |
| *Bifidobacterium longum* subsp. *longum* | MCC10025 | SAMN06368588 | 2.40884 | 60.1 | 1951 | GCF_004333845.1_ASM433384v1_genomic.fna |
| *Bifidobacterium longum* subsp. *longum* | MCC10023 | SAMN06368587 | 2.30816 | 60.3 | 1875 | GCF_004333855.1_ASM433385v1_genomic.fna |
| *Bifidobacterium longum* subsp. *longum* | MCC10021 | SAMN06368585 | 2.23575 | 60 | 1818 | GCF_004333875.1_ASM433387v1_genomic.fna |
| *Bifidobacterium longum* subsp. *longum* | MCC10022 | SAMN06368586 | 2.41904 | 59.7 | 2000 | GCF_004333895.1_ASM433389v1_genomic.fna |
| *Bifidobacterium longum* subsp. *longum* | MCC10018 | SAMN06368583 | 2.33041 | 60 | 1911 | GCF_004333905.1_ASM433390v1_genomic.fna |
| *Bifidobacterium longum* subsp. *longum* | MCC10015 | SAMN06368580 | 2.63029 | 59.9 | 2198 | GCF_004333925.1_ASM433392v1_genomic.fna |
| *Bifidobacterium longum* subsp. *longum* | MCC10017 | SAMN06368582 | 2.44883 | 59.7 | 2016 | GCF_004333935.1_ASM433393v1_genomic.fna |
| *Bifidobacterium longum* subsp. *longum* | MCC10014 | SAMN06368579 | 2.46621 | 60.2 | 2064 | GCF_004333975.1_ASM433397v1_genomic.fna |
| *Bifidobacterium longum* subsp. *longum* | MCC10012 | SAMN06368578 | 2.47987 | 59.6 | 2016 | GCF_004333995.1_ASM433399v1_genomic.fna |
| *Bifidobacterium longum* subsp. *longum* | MCC10010 | SAMN06368576 | 2.45245 | 60.4 | 2013 | GCF_004334005.1_ASM433400v1_genomic.fna |
| *Bifidobacterium longum* subsp. *longum* | MCC10008 | SAMN06368574 | 2.54367 | 60 | 2151 | GCF_004334035.1_ASM433403v1_genomic.fna |

| | | | | | | |
|---|---|---|---|---|---|---|
| *Bifidobacterium longum* subsp. *longum* | MCC10007 | SAMN06368573 | 2.48463 | 60.2 | 2046 | GCF_004334045.1_ASM433404v1_genomic.fna |
| *Bifidobacterium longum* subsp. *longum* | MCC10004 | SAMN06368571 | 2.55173 | 60 | 2072 | GCF_004334065.1_ASM433406v1_genomic.fna |
| *Bifidobacterium longum* subsp. *longum* | MCC10003 | SAMN06368570 | 2.52677 | 60.1 | 2074 | GCF_004334075.1_ASM433407v1_genomic.fna |
| *Bifidobacterium longum* subsp. *longum* | MCC10085 | SAMN06368640 | 2.3663 | 60 | 1931 | GCF_004334105.1_ASM433410v1_genomic.fna |
| *Bifidobacterium longum* subsp. *longum* | MCC10083 | SAMN06368638 | 2.47557 | 60.2 | 1987 | GCF_004334145.1_ASM433414v1_genomic.fna |
| *Bifidobacterium longum* subsp. *longum* | MCC10077 | SAMN06368633 | 2.41162 | 60 | 1998 | GCF_004334155.1_ASM433415v1_genomic.fna |
| *Bifidobacterium longum* subsp. *longum* | MCC10074 | SAMN06368630 | 2.41001 | 59.8 | 2024 | GCF_004334165.1_ASM433416v1_genomic.fna |
| *Bifidobacterium longum* subsp. *longum* | MCC10072 | SAMN06368628 | 2.23388 | 60 | 1785 | GCF_004334205.1_ASM433420v1_genomic.fna |
| *Bifidobacterium longum* subsp. *longum* | MCC10058 | SAMN06368617 | 2.32956 | 60.2 | 1909 | GCF_004334215.1_ASM433421v1_genomic.fna |
| *Bifidobacterium longum* subsp. *longum* | MCC10059 | SAMN06368618 | 2.43718 | 60 | 2054 | GCF_004334235.1_ASM433423v1_genomic.fna |
| *Bifidobacterium longum* subsp. *longum* | MCC10054 | SAMN06368613 | 2.29564 | 60 | 1885 | GCF_004334245.1_ASM433424v1_genomic.fna |
| *Bifidobacterium longum* subsp. *longum* | MCC10057 | SAMN06368616 | 2.18862 | 59.9 | 1764 | GCF_004334255.1_ASM433425v1_genomic.fna |
| *Bifidobacterium longum* subsp. *longum* | MCC10051 | SAMN06368610 | 2.30363 | 60.1 | 1882 | GCF_004334285.1_ASM433428v1_genomic.fna |
| *Bifidobacterium longum* subsp. *longum* | MCC10047 | SAMN06368607 | 2.36451 | 59.9 | 1898 | GCF_004334325.1_ASM433432v1_genomic.fna |
| *Bifidobacterium longum* subsp. *longum* | MCC10039 | SAMN06368599 | 2.38905 | 60 | 1956 | GCF_004334335.1_ASM433433v1_genomic.fna |

| | | | | | | |
|---|---|---|---|---|---|---|
| *Bifidobacterium longum* subsp. *longum* | MCC10040 | SAMN06368600 | 2.44996 | 60.2 | 2042 | GCF_004334345.1_ASM433434v1_genomic.fna |
| *Bifidobacterium longum* subsp. *longum* | MCC10030 | SAMN06368592 | 2.51844 | 60.1 | 2049 | GCF_004334355.1_ASM433435v1_genomic.fna |
| *Bifidobacterium longum* subsp. *longum* | MCC10038 | SAMN06368598 | 2.36701 | 59.9 | 2007 | GCF_004334365.1_ASM433436v1_genomic.fna |
| *Bifidobacterium longum* subsp. *longum* | MCC10028 | SAMN06368590 | 2.42541 | 60.2 | 2022 | GCF_004334425.1_ASM433442v1_genomic.fna |
| *Bifidobacterium longum* subsp. *longum* | MCC10019 | SAMN06368584 | 2.30453 | 60.1 | 1888 | GCF_004334435.1_ASM433443v1_genomic.fna |
| *Bifidobacterium longum* subsp. *longum* | MCC10027 | SAMN06368589 | 2.50583 | 59.9 | 2081 | GCF_004334445.1_ASM433444v1_genomic.fna |
| *Bifidobacterium longum* subsp. *longum* | MCC10016 | SAMN06368581 | 2.37118 | 60 | 1930 | GCF_004334465.1_ASM433446v1_genomic.fna |
| *Bifidobacterium longum* subsp. *longum* | MCC10011 | SAMN06368577 | 2.39746 | 59.9 | 1961 | GCF_004334485.1_ASM433448v1_genomic.fna |
| *Bifidobacterium longum* subsp. *longum* | MCC10009 | SAMN06368575 | 2.5237 | 60.1 | 2061 | GCF_004334515.1_ASM433451v1_genomic.fna |
| *Bifidobacterium longum* subsp. *longum* | MCC10006 | SAMN06368572 | 2.45014 | 60.4 | 2026 | GCF_004334535.1_ASM433453v1_genomic.fna |
| *Bifidobacterium longum* subsp. *longum* | MCC10002 | SAMN06368569 | 2.63451 | 60 | 2226 | GCF_004334545.1_ASM433454v1_genomic.fna |
| *Bifidobacterium longum* subsp. *longum* | MCC10212 | SAMN06368681 | 2.36547 | 59.9 | 1943 | GCF_004334555.1_ASM433455v1_genomic.fna |
| *Bifidobacterium longum* subsp. *longum* | MCC10127 | SAMN06368677 | 2.35673 | 60.1 | 1914 | GCF_004334615.1_ASM433461v1_genomic.fna |
| *Bifidobacterium longum* subsp. *longum* | MCC10130 | SAMN06368680 | 2.36857 | 60 | 1931 | GCF_004334625.1_ASM433462v1_genomic.fna |
| *Bifidobacterium longum* subsp. *longum* | MCC10126 | SAMN06368676 | 2.55307 | 59.8 | 2096 | GCF_004334635.1_ASM433463v1_genomic.fna |

| | | | | | | |
|---|---|---|---|---|---|---|
| *Bifidobacterium longum* subsp. *longum* | MCC10124 | SAMN06368674 | 2.45702 | 60.1 | 2015 | GCF_004334645.1_ASM433464v1_genomic.fna |
| *Bifidobacterium longum* subsp. *longum* | MCC10121 | SAMN06368671 | 2.38447 | 60 | 1954 | GCF_004334695.1_ASM433469v1_genomic.fna |
| *Bifidobacterium longum* subsp. *longum* | MCC10118 | SAMN06368668 | 2.34975 | 59.9 | 1934 | GCF_004334705.1_ASM433470v1_genomic.fna |
| *Bifidobacterium longum* subsp. *longum* | MCC10120 | SAMN06368670 | 2.48377 | 60.2 | 2047 | GCF_004334715.1_ASM433471v1_genomic.fna |
| *Bifidobacterium longum* subsp. *longum* | MCC10117 | SAMN06368667 | 2.30167 | 59.9 | 1829 | GCF_004334745.1_ASM433474v1_genomic.fna |
| *Bifidobacterium longum* subsp. *longum* | MCC10129 | SAMN06368679 | 2.27353 | 60.1 | 1854 | GCF_004334775.1_ASM433477v1_genomic.fna |
| *Bifidobacterium longum* subsp. *longum* | MCC10128 | SAMN06368678 | 2.50961 | 59.9 | 2112 | GCF_004334785.1_ASM433478v1_genomic.fna |
| *Bifidobacterium longum* subsp. *longum* | MCC10125 | SAMN06368675 | 2.44774 | 60.2 | 2011 | GCF_004334795.1_ASM433479v1_genomic.fna |
| *Bifidobacterium longum* subsp. *longum* | MCC10123 | SAMN06368673 | 2.5065 | 59.7 | 2074 | GCF_004334815.1_ASM433481v1_genomic.fna |
| *Bifidobacterium longum* subsp. *longum* | MCC10122 | SAMN06368672 | 2.46043 | 60.1 | 1999 | GCF_004334855.1_ASM433485v1_genomic.fna |
| *Bifidobacterium longum* subsp. *longum* | MCC10119 | SAMN06368669 | 2.48689 | 60.1 | 2074 | GCF_004334865.1_ASM433486v1_genomic.fna |
| *Bifidobacterium longum* subsp. *longum* 1-5B | 1-5B | SAMN02862991 | 2.36751 | 60.1 | 1929 | GCF_000730105.1_ASM73010v1_genomic.fna |
| *Bifidobacterium longum* subsp. *longum* 1-6B | 1-6B | SAMN00829154 | 2.68677 | 59.6 | 2233 | GCF_000261245.1_Blongum16Bv1.0_genomic.fna |
| *Bifidobacterium longum* subsp. *longum* 17-1B | 17-1B | SAMN02862993 | 2.4672 | 60.2 | 1983 | GCF_000730035.1_ASM73003v1_genomic.fna |
| *Bifidobacterium longum* subsp. *longum* 2-2B | 2-2B | SAMN00829155 | 2.6257 | 59.7 | 2111 | GCF_000261205.1_Blongum22Bv1.0_genomic.fna |

| | | | | | | |
|---|---|---|---|---|---|---|
| *Bifidobacterium longum* subsp. *longum* 35B | 35B | SAMN00829158 | 2.51443 | 60.1 | 2000 | GCF_000261225.1_Blongum35Bv1.0_genomic.fna |
| *Bifidobacterium longum* subsp. *longum* 44B | 44B | SAMN00829148 | 2.55922 | 59.7 | 2144 | GCF_000261265.1_Blongum44Bv1.0_genomic.fna |
| *Bifidobacterium longum* subsp. *longum* 7-1B | 7-1B | SAMN02862992 | 2.40709 | 59.8 | 1939 | GCF_000730055.1_ASM73005v1_genomic.fna |
| *Bifidobacterium longum* subsp. *longum* 72B | 72B | SAMN02862994 | 2.37445 | 60.3 | 1961 | GCF_000730045.1_ASM73004v1_genomic.fna |
| *Bifidobacterium longum* subsp. *longum* ATCC 55813 | ATCC 55813 | SAMN00001475 | 2.39636 | 60.1 | 1908 | GCF_000003135.1_ASM313v1_genomic.fna |
| *Bifidobacterium longum* subsp. *longum* BBMN68 | BBMN68 | SAMN02603469 | 2.26594 | 59.9 | 1809 | GCF_000166315.1_ASM16631v1_genomic.fna |
| *Bifidobacterium longum* subsp. *longum* CECT 7347 | CECT 7347 | SAMEA3146249 | 2.32722 | 60 | 1855 | GCF_001050555.1_ASM105055v1_genomic.fna |
| *Bifidobacterium longum* subsp. *longum* CMCC P0001 | CMCC P0001 | SAMN02470970 | 2.42435 | 59.7 | 1892 | GCF_000410595.1_ASM41059v1_genomic.fna |
| *Bifidobacterium longum* subsp. *longum* EK13 | EK13 | SAMN02862997 | 2.47453 | 60 | 2067 | GCF_000730135.1_ASM73013v1_genomic.fna |
| *Bifidobacterium longum* subsp. *longum* EK5 | EK5 | SAMN02862996 | 2.23129 | 59.7 | 1795 | GCF_000730025.1_ASM73002v1_genomic.fna |
| *Bifidobacterium longum* subsp. *longum* F8 | F8 | SAMEA3138379 | 2.38499 | 59.9 | 1929 | GCF_000210755.1_ASM21075v1_genomic.fna |
| *Bifidobacterium longum* subsp. *longum* GT15 | GT15 | SAMN03093230 | 2.33752 | 60 | 1844 | GCF_000772485.1_ASM77248v1_genomic.fna |
| *Bifidobacterium longum* subsp. *longum* JCM 1217 | JCM 1217 | SAMD00060951 | 2.38516 | 60.3 | 1920 | GCF_000196555.1_ASM19655v1_genomic.fna |
| *Bifidobacterium longum* subsp. *longum* JDM301 | JDM301 | SAMN02603181 | 2.47784 | 59.8 | 1955 | GCF_000092325.1_ASM9232v1_genomic.fna |
| *Bifidobacterium longum* subsp. *longum* KACC 91563 | KACC 91563 | SAMN02603656 | 2.39576 | 59.8115 | 1906 | GCF_000219455.1_ASM21945v1_genomic.fna |

| | | | | | | |
|---|---|---|---|---|---|---|
| *Bifidobacterium longum* subsp. *suis* | LMG 21814 | SAMN02673438 | 2.33583 | 60 | 1823 | GCF_000741625.1_Biflon_sub.sui_genomic.fna |
| *Bifidobacterium longum* subsp. *suis* | BSM11-5 | SAMN05578880 | 2.60288 | 59.9 | 2043 | GCF_001870705.1_ASM187070v1_genomic.fna |
| *Bifidobacterium longum* subsp. *suis* DSM 20211 | DSM 20211 | SAMN02442020 | 2.38997 | 60 | 1885 | GCF_000771285.1_DSM-20211_genomic.fna |
| *Bifidobacterium magnum* | LMG 11591 | SAMN02673439 | 1.82248 | 58.7 | 1462 | GCF_000741255.1_Bifmag_genomic.fna |
| *Bifidobacterium magnum* DSM 20222 | DSM 20222 | SAMN02441586 | 1.82526 | 58.7 | 1476 | GCF_000420565.1_ASM42056v1_genomic.fna |
| *Bifidobacterium magnum* DSM 20222 | DSM 20222 | SAMN02442024 | 1.81923 | 58.7 | 1473 | GCF_000771365.1_DSM-20222_genomic.fna |
| *Bifidobacterium merycicum* | LMG 11341 | SAMN02673440 | 2.28023 | 60.3 | 1710 | GCF_000741615.1_Bifmer_genomic.fna |
| *Bifidobacterium merycicum* | RUG133 | SAMEA104666290 | 1.90377 | 61.1 | 1424 | GCF_900315825.1_Rumen_uncultured_genome_RUG133_genomic.fna |
| *Bifidobacterium merycicum* DSM 6492 | DSM 6492 | SAMN02442042 | 2.27843 | 60.3 | 1734 | GCF_000770945.1_DSM-6492_genomic.fna |
| *Bifidobacterium merycicum* DSM 6492 | DSM 6492 | SAMN02745589 | 2.27468 | 60.4 | 1745 | GCF_900129045.1_IMG-taxon_2563366502_annotated_assembly_genomic.fna |
| *Bifidobacterium minimum* | LMG 11592 | SAMN02673441 | 1.89286 | 62.7 | 1434 | GCF_000741645.1_Bifmin_genomic.fna |
| *Bifidobacterium minimum* DSM 20102 | DSM 20102 | SAMN02440587 | 1.86732 | 62.7 | 1447 | GCF_000421685.1_ASM42168v1_genomic.fna |
| *Bifidobacterium minimum* DSM 20102 | DSM 20102 | SAMN02442018 | 1.86381 | 62.7 | 1435 | GCF_000771245.1_DSM-20102_genomic.fna |
| *Bifidobacterium mongoliense* | BMONG18 | SAMN09711768 | 2.13251 | 62.7 | 1658 | GCF_003788965.1_ASM378896v1_genomic.fna |
| *Bifidobacterium mongoliense* DSM 21395 | DSM 21395 | SAMN02673442 | 2.17049 | 62.8 | 1674 | GCF_000741285.1_Bifmon_genomic.fna |
| *Bifidobacterium mongoliense* DSM 21395 | DSM 21395 | SAMN02442032 | 2.15405 | 62.8 | 1686 | GCF_000771525.1_DSM-21395_genomic.fna |
| *Bifidobacterium moukalabense* | GG01 | SAMD00158983 | 2.52266 | 59.9 | - | GCF_005405645.1_ASM540564v1_genomic.fna |
| *Bifidobacterium moukalabense* | GB01 | SAMD00158984 | 2.52308 | 59.9 | - | GCF_005405665.1_ASM540566v1_genomic.fna |
| *Bifidobacterium moukalabense* | GB03 | SAMD00158985 | 2.52357 | 59.9 | - | GCF_005405685.1_ASM540568v1_genomic.fna |
| *Bifidobacterium moukalabense* | GB04 | SAMD00158986 | 2.52286 | 59.9 | - | GCF_005405705.1_ASM540570v1_genomic.fna |
| *Bifidobacterium moukalabense* | GB62 | SAMD00158987 | 2.54813 | 59.8 | - | GCF_005405725.1_ASM540572v1_genomic.fna |

| | | | | | | |
|---|---|---|---|---|---|---|
| *Bifidobacterium moukalabense* | GB63 | SAMD00158988 | 2.54573 | 59.8 | - | GCF_005405745.1_ASM540574v1_genomic.fna |
| *Bifidobacterium moukalabense* | GB65 | SAMD00158989 | 2.59608 | 59.9 | - | GCF_005405765.1_ASM540576v1_genomic.fna |
| *Bifidobacterium moukalabense* | CD14 | SAMD00158990 | 2.4513 | 59.8 | - | GCF_005405785.1_ASM540578v1_genomic.fna |
| *Bifidobacterium moukalabense* | CD16 | SAMD00158991 | 2.40562 | 59.8 | - | GCF_005405805.1_ASM540580v1_genomic.fna |
| *Bifidobacterium moukalabense* | CD33 | SAMD00158992 | 2.51575 | 59.9 | - | GCF_005405825.1_ASM540582v1_genomic.fna |
| *Bifidobacterium moukalabense* | EB43 | SAMD00158993 | 2.45141 | 59.8 | - | GCF_005405845.1_ASM540584v1_genomic.fna |
| *Bifidobacterium moukalabense* | EB44 | SAMD00158994 | 2.40379 | 59.8 | - | GCF_005405865.1_ASM540586v1_genomic.fna |
| ***Bifidobacterium moukalabense* DSM 27321** | **DSM 27321** | **SAMN02641635** | **2.51533** | **59.9** | **1987** | **GCF_000522505.1_DSM27321_genomic.fna** |
| *Bifidobacterium pseudocatenulatum* | 121.5 | SAMN04497914 | 1.24379 | 56.7 | 986 | GCA_001576885.1_ASM157688v1_genomic.fna |
| *Bifidobacterium pseudocatenulatum* | CF01-1 | SAMN09736656 | 2.37824 | 56.4 | 1541 | GCA_003463615.1_ASM346361v1_genomic.fna |
| *Bifidobacterium pseudocatenulatum* | CECT 7765 | SAMEA2743237 | 2.25434 | 56.4 | 1735 | GCF_000940535.1_BPSEU7765_v1_genomic.fna |
| *Bifidobacterium pseudocatenulatum* | 2789STDY5834840 | SAMEA3545306 | 2.30387 | 56.5 | 1871 | GCF_001405035.1_13470_2_59_genomic.fna |
| *Bifidobacterium pseudocatenulatum* | CA-05 | SAMD00047609 | 2.22312 | 56.4 | 1679 | GCF_001685965.1_ASM168596v1_genomic.fna |
| *Bifidobacterium pseudocatenulatum* | CA-B29 | SAMD00047610 | 2.3007 | 56.4 | 1761 | GCF_001685985.1_ASM168598v1_genomic.fna |
| *Bifidobacterium pseudocatenulatum* | CA-C29 | SAMD00047611 | 2.18207 | 56.3 | 1669 | GCF_001686005.1_ASM168600v1_genomic.fna |
| *Bifidobacterium pseudocatenulatum* | CA-D29 | SAMD00047612 | 2.26468 | 56.3 | 1750 | GCF_001686025.1_ASM168602v1_genomic.fna |
| *Bifidobacterium pseudocatenulatum* | CA-K29a | SAMD00047613 | 2.45851 | 56.4 | 1952 | GCF_001686045.1_ASM168604v1_genomic.fna |
| *Bifidobacterium pseudocatenulatum* | CA-K29b | SAMD00047614 | 2.45334 | 56.7 | 1945 | GCF_001686065.1_ASM168606v1_genomic.fna |
| *Bifidobacterium pseudocatenulatum* | 1896B | SAMN06621714 | 2.19747 | 56.2 | 1673 | GCF_002075945.1_Bbif1896B_genomic.fna |
| *Bifidobacterium pseudocatenulatum* | 1E | SAMN05933032 | 2.37266 | 56.9 | 1848 | GCF_002271255.1_Bpse1Ev1_genomic.fna |
| *Bifidobacterium pseudocatenulatum* | TM10-1 | SAMN09737021 | 2.22534 | 56.2 | 1704 | GCF_003436025.1_ASM343602v1_genomic.fna |
| *Bifidobacterium pseudocatenulatum* | TM08-2 | SAMN09737009 | 2.33019 | 56.5 | 1539 | GCF_003436105.1_ASM343610v1_genomic.fna |
| *Bifidobacterium pseudocatenulatum* | TM07-3AT | SAMN09737005 | 2.24252 | 56.3 | 1707 | GCF_003436315.1_ASM343631v1_genomic.fna |
| *Bifidobacterium pseudocatenulatum* | TM04-13 | SAMN09736969 | 2.26457 | 56.5 | 1735 | GCF_003436545.1_ASM343654v1_genomic.fna |
| *Bifidobacterium pseudocatenulatum* | TM01-4 | SAMN09736963 | 2.18974 | 56.3 | 1289 | GCF_003436675.1_ASM343667v1_genomic.fna |
| *Bifidobacterium pseudocatenulatum* | TF08-3AT | SAMN09736917 | 2.26167 | 56.3 | 1756 | GCF_003436955.1_ASM343695v1_genomic.fna |
| *Bifidobacterium pseudocatenulatum* | TF07-45 | SAMN09736909 | 2.38374 | 56.6 | 1890 | GCF_003437075.1_ASM343707v1_genomic.fna |
| *Bifidobacterium pseudocatenulatum* | TF07-23 | SAMN09736905 | 2.33833 | 56.7 | 1809 | GCF_003437155.1_ASM343715v1_genomic.fna |

| | | | | | | |
|---|---|---|---|---|---|---|
| *Bifidobacterium pseudocatenulatum* | TM05-11 | SAMN09736977 | 2.29419 | 56.4 | 1806 | GCF_003437435.1_ASM343743v1_genomic.fna |
| *Bifidobacterium pseudocatenulatum* | TF05-2AC | SAMN09736882 | 2.20629 | 56.6 | 1725 | GCF_003437825.1_ASM343782v1_genomic.fna |
| *Bifidobacterium pseudocatenulatum* | TF05-19AC | SAMN09736879 | 2.1764 | 56.4 | 1680 | GCF_003437835.1_ASM343783v1_genomic.fna |
| *Bifidobacterium pseudocatenulatum* | OM10-8 | SAMN09736863 | 2.20095 | 56.3 | 1382 | GCF_003438015.1_ASM343801v1_genomic.fna |
| *Bifidobacterium pseudocatenulatum* | OM05-2 | SAMN09736800 | 2.3654 | 56.6 | 1856 | GCF_003438405.1_ASM343840v1_genomic.fna |
| *Bifidobacterium pseudocatenulatum* | OF05-12 | SAMN09736713 | 2.36975 | 56.8 | 1898 | GCF_003439655.1_ASM343965v1_genomic.fna |
| *Bifidobacterium pseudocatenulatum* | AF26-1 | SAMN09734507 | 2.1833 | 56.6 | 1712 | GCF_003458965.1_ASM345896v1_genomic.fna |
| *Bifidobacterium pseudocatenulatum* | AF20-20AC | SAMN09734414 | 2.24914 | 56.5 | 1745 | GCF_003459475.1_ASM345947v1_genomic.fna |
| *Bifidobacterium pseudocatenulatum* | AF18-2AC | SAMN09734359 | 2.37026 | 56.7 | 1521 | GCF_003459865.1_ASM345986v1_genomic.fna |
| *Bifidobacterium pseudocatenulatum* | AF17-20AC | SAMN09734331 | 2.23889 | 56.2 | 1713 | GCF_003460425.1_ASM346042v1_genomic.fna |
| *Bifidobacterium pseudocatenulatum* | OF01-21AC | SAMN09736671 | 2.25277 | 56.5 | 1431 | GCF_003463265.1_ASM346326v1_genomic.fna |
| *Bifidobacterium pseudocatenulatum* | OF01-8 | SAMN09736676 | 2.28785 | 56.6 | 1795 | GCF_003463425.1_ASM346342v1_genomic.fna |
| *Bifidobacterium pseudocatenulatum* | OF01-2 | SAMN09736669 | 2.24892 | 56.5 | 1740 | GCF_003463455.1_ASM346345v1_genomic.fna |
| *Bifidobacterium pseudocatenulatum* | OF01-12 | SAMN09736662 | 2.28465 | 56.6 | 1793 | GCF_003463505.1_ASM346350v1_genomic.fna |
| *Bifidobacterium pseudocatenulatum* | AF12-8LB-d | SAMN09734227 | 2.20298 | 56.2 | 1702 | GCF_003464925.1_ASM346492v1_genomic.fna |
| *Bifidobacterium pseudocatenulatum* | AF12-10-6.0 | SAMN09734213 | 2.20223 | 56.2 | 1706 | GCF_003465065.1_ASM346506v1_genomic.fna |
| *Bifidobacterium pseudocatenulatum* | AF11-18 | SAMN09734208 | 2.26795 | 56.2 | 1745 | GCF_003465135.1_ASM346513v1_genomic.fna |
| *Bifidobacterium pseudocatenulatum* | AF12-8A-LB | SAMN09734226 | 2.20378 | 56.2 | 1702 | GCF_003465385.1_ASM346538v1_genomic.fna |
| *Bifidobacterium pseudocatenulatum* | AF03-28 | SAMN09734173 | 2.36028 | 56.6 | 1835 | GCF_003465775.1_ASM346577v1_genomic.fna |
| *Bifidobacterium pseudocatenulatum* | AF02-36-1 | SAMN09734165 | 2.1851 | 56.3 | 1669 | GCF_003466105.1_ASM346610v1_genomic.fna |
| *Bifidobacterium pseudocatenulatum* | AM43-10 | SAMN09736560 | 2.19896 | 56.5 | 1682 | GCF_003467065.1_ASM346706v1_genomic.fna |
| *Bifidobacterium pseudocatenulatum* | AM38-8 | SAMN09736506 | 2.30925 | 56.2 | 1786 | GCF_003467515.1_ASM346751v1_genomic.fna |
| *Bifidobacterium pseudocatenulatum* | AM36-5BH | SAMN09736486 | 2.1639 | 56.2 | 1676 | GCF_003467755.1_ASM346775v1_genomic.fna |
| *Bifidobacterium pseudocatenulatum* | AM36-2AC | SAMN09736481 | 2.16318 | 56.2 | 1675 | GCF_003467785.1_ASM346778v1_genomic.fna |
| *Bifidobacterium pseudocatenulatum* | AM33-6 | SAMN09736456 | 2.26145 | 56.4 | 1764 | GCF_003468555.1_ASM346855v1_genomic.fna |
| *Bifidobacterium pseudocatenulatum* | AM26-14LB | SAMN09736330 | 2.21856 | 56.2 | 1724 | GCF_003470125.1_ASM347012v1_genomic.fna |
| *Bifidobacterium pseudocatenulatum* | AM19-19 | SAMN09734878 | 2.18198 | 56.1 | 1374 | GCF_003470545.1_ASM347054v1_genomic.fna |
| *Bifidobacterium pseudocatenulatum* | AM20-9-6.0 | SAMN09734886 | 2.15943 | 56.3 | 1684 | GCF_003471295.1_ASM347129v1_genomic.fna |
| *Bifidobacterium pseudocatenulatum* | AM20-6 | SAMN09734885 | 2.23064 | 56.6 | 1773 | GCF_003471325.1_ASM347132v1_genomic.fna |

| | | | | | | |
|---|---|---|---|---|---|---|
| *Bifidobacterium pseudocatenulatum* | AM20-1 | SAMN09734879 | 2.23797 | 56.6 | 1771 | GCF_003471415.1_ASM347141v1_genomic.fna |
| *Bifidobacterium pseudocatenulatum* | AM18-42 | SAMN09734871 | 2.22506 | 56.2 | 1695 | GCF_003471505.1_ASM347150v1_genomic.fna |
| *Bifidobacterium pseudocatenulatum* | AM11-10 | SAMN09734778 | 2.33619 | 56.5 | 1848 | GCF_003472415.1_ASM347241v1_genomic.fna |
| *Bifidobacterium pseudocatenulatum* | AM10-24 | SAMN09734769 | 2.14238 | 56.4 | 1662 | GCF_003472545.1_ASM347254v1_genomic.fna |
| *Bifidobacterium pseudocatenulatum* | AM10-2 | SAMN09734766 | 2.14287 | 56.4 | 1661 | GCF_003472575.1_ASM347257v1_genomic.fna |
| *Bifidobacterium pseudocatenulatum* | AM08-25 | SAMN09734749 | 2.33946 | 56.4 | 1787 | GCF_003472685.1_ASM347268v1_genomic.fna |
| *Bifidobacterium pseudocatenulatum* | AM08-2 | SAMN09734748 | 2.32705 | 56.5 | 1779 | GCF_003472725.1_ASM347272v1_genomic.fna |
| *Bifidobacterium pseudocatenulatum* | AM13-8 | SAMN09734801 | 2.21638 | 56.3 | 1717 | GCF_003473025.1_ASM347302v1_genomic.fna |
| *Bifidobacterium pseudocatenulatum* | AM13-2 | SAMN09734796 | 2.21441 | 56.3 | 1715 | GCF_003473115.1_ASM347311v1_genomic.fna |
| *Bifidobacterium pseudocatenulatum* | AF45-10BH | SAMN09734713 | 2.27871 | 56.6 | 1485 | GCF_003474015.1_ASM347401v1_genomic.fna |
| *Bifidobacterium pseudocatenulatum* | AF41-3MH | SAMN09734692 | 2.26022 | 56.5 | 1378 | GCF_003474525.1_ASM347452v1_genomic.fna |
| *Bifidobacterium pseudocatenulatum* | AF36-12AT | SAMN09734638 | 2.35922 | 56.6 | 1889 | GCF_003474835.1_ASM347483v1_genomic.fna |
| *Bifidobacterium pseudocatenulatum* | 12 | SAMN08116046 | 2.19239 | 56.5 | 1657 | GCF_003952825.1_ASM395282v1_genomic.fna |
| *Bifidobacterium pseudocatenulatum* | ca_0067 | SAMN10239588 | 2.10619 | 56.3 | 1626 | GCF_004167565.1_ASM416756v1_genomic.fna |
| *Bifidobacterium pseudocatenulatum* | 1001271st1_F3 | SAMN10863262 | 2.18083 | 56.2 | - | GCF_005844335.1_ASM584433v1_genomic.fna |
| *Bifidobacterium pseudocatenulatum* DSM 20438 = JCM 1200 = LMG 10505 | DSM 20438 | SAMN00008796 | 2.30481 | 56.3 | 1750 | GCF_000173435.1_ASM17343v1_genomic.fna |
| *Bifidobacterium pseudocatenulatum* DSM 20438 = JCM 1200 = LMG 10505 | LMG 10505 | SAMN02673443 | 2.28377 | 56.4 | 1700 | GCF_000741685.1_Bifpsetum_genomic.fna |
| *Bifidobacterium pseudocatenulatum* DSM 20438 = JCM 1200 = LMG 10505 | DSM 20438 | SAMN02442029 | 2.28449 | 56.4 | 1746 | GCF_000771445.1_DSM-20438_genomic.fna |
| ***Bifidobacterium pseudocatenulatum* DSM 20438 = JCM 1200 = LMG 10505** | **JCM 1200** | **SAMD00061045** | **2.31375** | **56.4** | **1759** | **GCF_001025215.1_ASM102521v1_genomic.fna** |
| *Bifidobacterium pseudocatenulatum* IPLA36007 | IPLA36007 | SAMN02649570 | 2.32818 | 56.3 | 1814 | GCF_000708005.1_B.p.IPLA36007_genomic.fna |
| *Bifidobacterium pseudolongum* | UBA1157 | SAMN06457169 | 1.82503 | 62.1 | - | GCA_002311215.1_ASM231121v1_genomic.fna |

| | | | | | | |
|---|---|---|---|---|---|---|
| *Bifidobacterium pseudolongum* | UMB-MBP-01 | SAMN07357905 | 2.0081 | 63.4 | 1585 | GCF_002282915.1_ASM228291v1_genomic.fna |
| *Bifidobacterium pseudolongum* | AF13-3LB | SAMN09734239 | 2.21544 | 63 | 1798 | GCF_003464845.1_ASM346484v1_genomic.fna |
| *Bifidobacterium pseudolongum* | WYJ21-P61 | SAMN10024806 | 2.05379 | 63.3 | 1658 | GCF_003612445.1_ASM361244v1_genomic.fna |
| *Bifidobacterium pseudolongum* | NM87_A27A | SAMN11366404 | 2.21056 | 63.2 | 1787 | GCF_004801625.1_ASM480162v1_genomic.fna |
| *Bifidobacterium pseudolongum* AGR2145 | AGR2145 | SAMN02440890 | 1.9922 | 63.3 | 1566 | GCF_000421365.1_ASM42136v1_genomic.fna |
| ***Bifidobacterium pseudolongum* PV8-2** | **PV8-2** | **SAMN03253091** | **2.0327** | **63.3** | **1608** | **GCF_000800475.2_ASM80047v2_genomic.fna** |
| *Bifidobacterium pseudolongum* subsp. *globosum* | LMG 11569 | SAMN02673444 | 1.93525 | 63.4 | 1512 | GCF_000741295.1_Bifpse_sub.glo_genomic.fna |
| *Bifidobacterium pseudolongum* subsp. *globosum* | 1744B | SAMN07731043 | 2.14358 | 63.1 | 1674 | GCF_002846675.1_ASM284667v1_genomic.fna |
| *Bifidobacterium pseudolongum* subsp. *globosum* | 1619B | SAMN07731042 | 2.05041 | 63.3 | 1637 | GCF_002846685.1_ASM284668v1_genomic.fna |
| *Bifidobacterium pseudolongum* subsp. *globosum* | 1549B | SAMN07731038 | 1.9902 | 63.2 | 1569 | GCF_002846715.1_ASM284671v1_genomic.fna |
| *Bifidobacterium pseudolongum* subsp. *globosum* | 1520B | SAMN07731037 | 2.00848 | 63.1 | 1575 | GCF_002846775.1_ASM284677v1_genomic.fna |
| *Bifidobacterium pseudolongum* subsp. *globosum* | 1524B | SAMN07731045 | 2.06241 | 63.1 | 1634 | GCF_002846815.1_ASM284681v1_genomic.fna |
| *Bifidobacterium pseudolongum* subsp. *globosum* | 1747B | SAMN07731044 | 2.14308 | 63.2 | 1722 | GCF_002846835.1_ASM284683v1_genomic.fna |
| *Bifidobacterium pseudolongum* subsp. *globosum* | 1734B | SAMN07731041 | 2.11186 | 63.3 | 1692 | GCF_002846845.1_ASM284684v1_genomic.fna |
| *Bifidobacterium pseudolongum* subsp. *globosum* | 1691B | SAMN07731040 | 2.14872 | 63.1 | 1679 | GCF_002846875.1_ASM284687v1_genomic.fna |
| *Bifidobacterium pseudolongum* subsp. *globosum* | 2114B | SAMN10615463 | 2.0472 | 63.1 | 1598 | GCF_004154995.1_ASM415499v1_genomic.fna |
| *Bifidobacterium pseudolongum* subsp. *globosum* | 2083B | SAMN10615453 | 2.05982 | 63.1 | 1616 | GCF_004155045.1_ASM415504v1_genomic.fna |

| | | | | | | |
|---|---|---|---|---|---|---|
| *Bifidobacterium pseudolongum* subsp. *globosum* | 2019B | SAMN10615444 | 1.94314 | 63.3 | 1538 | GCF_004155095.1_ASM415509v1_genomic.fna |
| *Bifidobacterium pseudolongum* subsp. *globosum* | 2004B | SAMN10615440 | 1.91072 | 63.3 | 1507 | GCF_004155115.1_ASM415511v1_genomic.fna |
| *Bifidobacterium pseudolongum* subsp. *globosum* | 2071B | SAMN10615451 | 2.08465 | 63.2 | 1687 | GCF_004155135.1_ASM415513v1_genomic.fna |
| *Bifidobacterium pseudolongum* subsp. *globosum* | 2012B | SAMN10615442 | 2.07061 | 63.3 | 1682 | GCF_004155145.1_ASM415514v1_genomic.fna |
| *Bifidobacterium pseudolongum* subsp. *globosum* | 1678B | SAMN10615431 | 2.03349 | 63 | 1606 | GCF_004155155.1_ASM415515v1_genomic.fna |
| *Bifidobacterium pseudolongum* subsp. *globosum* | 2029B | SAMN10615446 | 2.13264 | 63.1 | 1671 | GCF_004155165.1_ASM415516v1_genomic.fna |
| *Bifidobacterium pseudolongum* subsp. *globosum* | 1511B | SAMN10615419 | 1.97106 | 63.2 | 1542 | GCF_004155235.1_ASM415523v1_genomic.fna |
| *Bifidobacterium pseudolongum* subsp. *globosum* | 2017B | SAMN10615443 | 2.18994 | 63 | 1812 | GCF_004155275.1_ASM415527v1_genomic.fna |
| *Bifidobacterium pseudolongum* subsp. *globosum* | 2000B | SAMN10615436 | 2.11231 | 63.2 | 1710 | GCF_004155285.1_ASM415528v1_genomic.fna |
| *Bifidobacterium pseudolongum* subsp. *globosum* | 2113B | SAMN10615462 | 1.99635 | 63.1 | 1581 | GCF_004155295.1_ASM415529v1_genomic.fna |
| *Bifidobacterium pseudolongum* subsp. *globosum* | 2003B | SAMN10615439 | 2.1486 | 63.2 | 1741 | GCF_004155305.1_ASM415530v1_genomic.fna |
| *Bifidobacterium pseudolongum* subsp. *globosum* | 22506 | SAMN10615465 | 1.91307 | 63.3 | 1494 | GCF_004155325.1_ASM415532v1_genomic.fna |
| *Bifidobacterium pseudolongum* subsp. *globosum* | 1805B | SAMN10615435 | 2.1027 | 63.1 | 1672 | GCF_004155375.1_ASM415537v1_genomic.fna |
| *Bifidobacterium pseudolongum* subsp. *globosum* | 102017 | SAMN10615468 | 1.88411 | 63.2 | 1487 | GCF_004155405.1_ASM415540v1_genomic.fna |
| *Bifidobacterium pseudolongum* subsp. *globosum* | 102015 | SAMN10615467 | 1.91818 | 63.1 | 1524 | GCF_004155425.1_ASM415542v1_genomic.fna |

| | | | | | | |
|---|---|---|---|---|---|---|
| *Bifidobacterium pseudolongum* subsp. *globosum* | 22511 | SAMN10615466 | 2.01887 | 62.9 | 1572 | GCF_004155435.1_ASM415543v1_genomic.fna |
| *Bifidobacterium pseudolongum* subsp. *globosum* | 2109B | SAMN10615461 | 2.03922 | 63.2 | 1617 | GCF_004155475.1_ASM415547v1_genomic.fna |
| *Bifidobacterium pseudolongum* subsp. *globosum* | 2098B | SAMN10615458 | 1.99835 | 63.1 | 1580 | GCF_004155495.1_ASM415549v1_genomic.fna |
| *Bifidobacterium pseudolongum* subsp. *globosum* | 2105B | SAMN10615460 | 1.96489 | 63.3 | 1553 | GCF_004155505.1_ASM415550v1_genomic.fna |
| *Bifidobacterium pseudolongum* subsp. *globosum* | 2089B | SAMN10615456 | 2.04748 | 63.1 | 1586 | GCF_004155525.1_ASM415552v1_genomic.fna |
| *Bifidobacterium pseudolongum* subsp. *globosum* | 2093B | SAMN10615457 | 2.13296 | 63.1 | 1684 | GCF_004155535.1_ASM415553v1_genomic.fna |
| *Bifidobacterium pseudolongum* subsp. *globosum* | 2072B | SAMN10615452 | 2.08284 | 63.2 | 1685 | GCF_004155565.1_ASM415556v1_genomic.fna |
| *Bifidobacterium pseudolongum* subsp. *globosum* | 2048B | SAMN10615448 | 2.03004 | 63.3 | 1637 | GCF_004155595.1_ASM415559v1_genomic.fna |
| *Bifidobacterium pseudolongum* subsp. *globosum* | 2032B | SAMN10615447 | 2.13219 | 63.1 | 1672 | GCF_004155615.1_ASM415561v1_genomic.fna |
| *Bifidobacterium pseudolongum* subsp. *globosum* | 2009B | SAMN10615441 | 2.10753 | 63 | 1668 | GCF_004155625.1_ASM415562v1_genomic.fna |
| *Bifidobacterium pseudolongum* subsp. *globosum* | 2023B | SAMN10615445 | 1.98377 | 63.6 | 1571 | GCF_004155635.1_ASM415563v1_genomic.fna |
| *Bifidobacterium pseudolongum* subsp. *globosum* | 2001B | SAMN10615437 | 2.09532 | 63.2 | 1670 | GCF_004155645.1_ASM415564v1_genomic.fna |
| *Bifidobacterium pseudolongum* subsp. *globosum* | 1791B | SAMN10615434 | 2.13055 | 62.8 | 1690 | GCF_004155695.1_ASM415569v1_genomic.fna |
| *Bifidobacterium pseudolongum* subsp. *globosum* | 1770B | SAMN10615432 | 2.11457 | 63 | 1685 | GCF_004155705.1_ASM415570v1_genomic.fna |
| *Bifidobacterium pseudolongum* subsp. *globosum* | 1578B | SAMN10615425 | 1.95007 | 63.5 | 1530 | GCF_004155745.1_ASM415574v1_genomic.fna |

| | | | | | | |
|---|---|---|---|---|---|---|
| *Bifidobacterium pseudolongum* subsp. *globosum* | 1565B | SAMN10615423 | 1.95659 | 63.3 | 1558 | GCF_004155795.1_ASM415579v1_genomic.fna |
| *Bifidobacterium pseudolongum* subsp. *globosum* | 1550B | SAMN10615422 | 2.00091 | 63.3 | 1599 | GCF_004155805.1_ASM415580v1_genomic.fna |
| *Bifidobacterium pseudolongum* subsp. *globosum* | 1546B | SAMN10615421 | 1.95552 | 63.3 | 1556 | GCF_004155845.1_ASM415584v1_genomic.fna |
| *Bifidobacterium pseudolongum* subsp. *globosum* | 112206 | SAMN10615469 | 1.94749 | 63.2 | 1528 | GCF_004155855.1_ASM415585v1_genomic.fna |
| *Bifidobacterium pseudolongum* subsp. *globosum* | 2088B | SAMN10615455 | 1.99177 | 63.1 | 1577 | GCF_004156075.1_ASM415607v1_genomic.fna |
| *Bifidobacterium pseudolongum* subsp. *globosum* | 2115B | SAMN10615464 | 2.01678 | 63.1 | 1584 | GCF_004156085.1_ASM415608v1_genomic.fna |
| *Bifidobacterium pseudolongum* subsp. *globosum* | 2049B | SAMN10615449 | 2.18995 | 63 | 1810 | GCF_004156115.1_ASM415611v1_genomic.fna |
| *Bifidobacterium pseudolongum* subsp. *globosum* | 2086B | SAMN10615454 | 1.99682 | 63.1 | 1581 | GCF_004156135.1_ASM415613v1_genomic.fna |
| *Bifidobacterium pseudolongum* subsp. *globosum* | 1616B | SAMN10615428 | 2.03179 | 63.3 | 1614 | GCF_004156145.1_ASM415614v1_genomic.fna |
| *Bifidobacterium pseudolongum* subsp. *globosum* | 2103B | SAMN10615459 | 2.08059 | 63.2 | 1667 | GCF_004156175.1_ASM415617v1_genomic.fna |
| *Bifidobacterium pseudolongum* subsp. *globosum* | 1655B | SAMN10615430 | 2.03177 | 63 | 1610 | GCF_004156195.1_ASM415619v1_genomic.fna |
| *Bifidobacterium pseudolongum* subsp. *globosum* | 1577B | SAMN10615424 | 1.94992 | 63.5 | 1530 | GCF_004156215.1_ASM415621v1_genomic.fna |
| *Bifidobacterium pseudolongum* subsp. *globosum* | 2002B | SAMN10615438 | 2.05796 | 63.3 | 1646 | GCF_004156235.1_ASM415623v1_genomic.fna |
| *Bifidobacterium pseudolongum* subsp. *globosum* | 1780B | SAMN10615433 | 1.98148 | 63.2 | 1552 | GCF_004168525.1_ASM416852v1_genomic.fna |
| *Bifidobacterium pseudolongum* subsp. *globosum* DSM 20092 | DSM 20092 | SAMN02743881 | 1.91305 | 63.4 | 1504 | GCF_000687595.1_ASM68759v1_genomic.fna |

| | | | | | | |
|---|---|---|---|---|---|---|
| *Bifidobacterium pseudolongum* subsp. *globosum* DSM 20092 | DSM 20092 | SAMN02442013 | 1.91504 | 63.4 | 1499 | GCF_000771145.1_DSM-20092_genomic.fna |
| *Bifidobacterium pseudolongum* subsp. *globosum* DSM 20092 | DSM 20092 | SAMN05912980 | 1.97816 | 63.3 | 1532 | GCF_002706665.1_ASM270666v1_genomic.fna |
| *Bifidobacterium pseudolongum* subsp. *pseudolongum* | LMG 11571 | SAMN02673445 | 1.89868 | 63.1 | 1475 | GCF_000741325.1_Bifpse_sub.pse_genomic.fna |
| *Bifidobacterium pseudolongum* subsp. *pseudolongum* | 1595B | SAMN07731039 | 1.93642 | 63 | 1515 | GCF_002846725.1_ASM284672v1_genomic.fna |
| *Bifidobacterium pseudolongum* subsp. *pseudolongum* | 1370B | SAMN07731036 | 1.90204 | 63 | 1514 | GCF_002846755.1_ASM284675v1_genomic.fna |
| *Bifidobacterium pseudolongum* subsp. *pseudolongum* | 2054B | SAMN10615450 | 1.97429 | 63 | 1565 | GCF_004155015.1_ASM415501v1_genomic.fna |
| *Bifidobacterium pseudolongum* subsp. *pseudolongum* | 1604B | SAMN10615426 | 1.88204 | 63 | 1487 | GCF_004155395.1_ASM415539v1_genomic.fna |
| *Bifidobacterium pseudolongum* subsp. *pseudolongum* | 1612B | SAMN10615427 | 1.8915 | 63.1 | 1512 | GCF_004155715.1_ASM415571v1_genomic.fna |
| *Bifidobacterium pseudolongum* subsp. *pseudolongum* | 1629B | SAMN10615429 | 1.85243 | 63 | 1475 | GCF_004155725.1_ASM415572v1_genomic.fna |
| *Bifidobacterium pseudolongum* subsp. *pseudolongum* | 1513B | SAMN10615420 | 1.85577 | 63.2 | 1473 | GCF_004155835.1_ASM415583v1_genomic.fna |
| *Bifidobacterium pseudolongum* subsp. *pseudolongum* DSM 20099 | DSM 20099 | SAMN02442017 | 1.901 | 63.1 | 1509 | GCF_000771225.1_DSM-20099_genomic.fna |
| *Bifidobacterium psychraerophilum* | LMG 21775 | SAMN02673446 | 2.61508 | 58.8 | 2081 | GCF_000741705.1_Bifpsy_genomic.fna |
| *Bifidobacterium psychraerophilum* DSM 22366 | DSM 22366 | SAMN02442034 | 2.62112 | 58.7 | 2083 | GCF_000771565.1_DSM-22366_genomic.fna |
| *Bifidobacterium psychraerophilum* DSM 22366 | DSM 22366 | SAMN05192537 | 2.63149 | 58.8 | 2085 | GCF_002813205.1_ASM281320v1_genomic.fna |
| *Bifidobacterium pullorum* | LMG 21816 | SAMN02673447 | 2.15356 | 64.2 | 1559 | GCF_000741335.1_Bifpul_genomic.fna |
| *Bifidobacterium pullorum* DSM 20433 | DSM 20433 | SAMN02442026 | 2.10095 | 64.3 | 1613 | GCF_000771405.1_DSM-20433_genomic.fna |

| | | | | | | |
|---|---|---|---|---|---|---|
| *Bifidobacterium reuteri* DSM 23975 | DSM 23975 | SAMN02673448 | 2.84757 | 60.5 | 2061 | GCF_000741695.1_Bifreu_genomic.fna |
| *Bifidobacterium reuteri* DSM 23975 | DSM 23975 | SAMN02442040 | 2.836 | 60.5 | 2079 | GCF_000771685.1_DSM-23975_genomic.fna |
| *Bifidobacterium ruminantium* | LMG 21811 | SAMN02673449 | 2.24981 | 59.2 | 1735 | GCF_000741365.1_Bifrum_genomic.fna |
| *Bifidobacterium ruminantium* DSM 6489 | DSM 6489 | SAMN02743880 | 2.21856 | 59.2 | 1723 | GCF_000687635.1_ASM68763v1_genomic.fna |
| *Bifidobacterium ruminantium* DSM 6489 | DSM 6489 | SAMN02442041 | 2.23033 | 59.2 | 1727 | GCF_000770925.1_DSM-6489_genomic.fna |
| *Bifidobacterium saeculare* DSM 6531 = LMG 14934 | LMG 14934 | SAMN02673450 | 2.26328 | 63.7 | 1723 | GCF_000741375.1_Bifsae_genomic.fna |
| *Bifidobacterium saeculare* DSM 6531 = LMG 14934 | DSM 6531 | SAMN02442043 | 2.25126 | 63.8 | 1741 | GCF_000770965.1_DSM-6531_genomic.fna |
| *Bifidobacterium saguini* DSM 23967 | DSM 23967 | SAMN02673451 | 2.78704 | 56.4 | 2178 | GCF_000741715.1_Bifsag_genomic.fna |
| *Bifidobacterium saguini* DSM 23967 | DSM 23967 | SAMN02442037 | 2.77343 | 56.3 | 2204 | GCF_000771625.1_DSM-23967_genomic.fna |
| *Bifidobacterium scardovii* | LMG 21589 | SAMN02673452 | 3.14179 | 64.6 | 2375 | GCF_000741405.1_Bifsca_genomic.fna |
| *Bifidobacterium scardovii* | 981_BLON | SAMN03198202 | 3.12129 | 64.8 | 2354 | GCF_001059475.1_ASM105947v1_genomic.fna |
| *Bifidobacterium scardovii* JCM 12489 = DSM 13734 | DSM 13734 | SAMN02442005 | 3.14662 | 64.7 | 2375 | GCF_000770985.1_DSM-13734_genomic.fna |
| *Bifidobacterium scardovii* JCM 12489 = DSM 13734 | DSM 13734 | SAMN03443793 | 3.14395 | 64.6 | 2374 | GCF_001005065.1_ASM100506v1_genomic.fna |
| *Bifidobacterium scardovii* JCM 12489 = DSM 13734 | JCM 12489 | SAMD00061046 | 3.15835 | 64.6 | 2397 | GCF_001042635.1_ASM104263v1_genomic.fna |
| *Bifidobacterium stercoris* JCM 15918 | DSM 24849 | SAMN02673454 | 2.30461 | 59.4 | 1831 | GCF_000741415.1_Bifste_genomic.fna |
| *Bifidobacterium stercoris* JCM 15918 | JCM 15918 | SAMN02442044 | 2.29198 | 59.4 | 1829 | GCF_000771705.1_JCM-15918_genomic.fna |
| *Bifidobacterium subtile* | LMG 11597 | SAMN02673455 | 2.79009 | 60.9 | 2163 | GCF_000741775.1_Bifsub_genomic.fna |

| | | | | | | |
|---|---|---|---|---|---|---|
| *Bifidobacterium subtile* DSM 20096 | DSM 20096 | SAMN02440843 | 2.76527 | 60.9 | 2139 | GCF_000426405.1_ASM42640v1_genomic.fna |
| *Bifidobacterium subtile* DSM 20096 | DSM 20096 | SAMN02442015 | 2.762 | 60.9 | 2129 | GCF_000771185.1_DSM-20096_genomic.fna |
| *Bifidobacterium thermacidophilum* subsp. *porcinum* | LMG 21689 | SAMN02673456 | 2.07937 | 60.2 | 1738 | GCF_000741445.1_Bifthe_sub.por_genomic.fna |
| *Bifidobacterium thermacidophilum* subsp. *porcinum* DSM 17755 | DSM 17755 | SAMN02442008 | 2.06257 | 60.2 | 1504 | GCF_000771045.1_DSM-17755_genomic.fna |
| *Bifidobacterium thermacidophilum* subsp. *thermacidophilum* | LMG 21395 | SAMN02673457 | 2.23307 | 60.4 | 1614 | GCF_000741455.1_Bifthe_sub.the_genomic.fna |
| *Bifidobacterium thermacidophilum* subsp. *thermacidophilum* DSM 15837 | DSM 15837 | SAMN02441522 | 2.22359 | 60.4 | 1642 | GCF_000426425.1_ASM42642v1_genomic.fna |
| *Bifidobacterium thermacidophilum* subsp. *thermacidophilum* DSM 15837 | DSM 15837 | SAMN02442006 | 2.22099 | 60.4 | 1634 | GCF_000771005.1_DSM-15837_genomic.fna |
| *Bifidobacterium thermophilum* | JCM 1207 | SAMN02673459 | 2.0995 | 59.9 | 1700 | GCF_000741495.1_Bifthelum_genomic.fna |
| *Bifidobacterium thermophilum* | 1543B | SAMN07731047 | 2.31613 | 60.4 | 1687 | GCF_002846655.1_ASM284665v1_genomic.fna |
| *Bifidobacterium thermophilum* | 1542B | SAMN07731046 | 2.35913 | 60.3 | 1702 | GCF_002846785.1_ASM284678v1_genomic.fna |
| *Bifidobacterium thermophilum* DSM 20210 | DSM 20210 | SAMN02442019 | 2.22484 | 59.7 | 1622 | GCF_000771265.1_DSM-20210_genomic.fna |
| *Bifidobacterium thermophilum* DSM 20212 | DSM 20212 | SAMN02743878 | 2.25235 | 60.1 | 1626 | GCF_000687575.1_ASM68757v1_genomic.fna |
| *Bifidobacterium thermophilum* RBL67 | RBL67 | SAMN02603226 | 2.29164 | 60.1 | 1641 | GCF_000347695.1_ASM34769v1_genomic.fna |
| *Bifidobacterium tsurumiense* | JCM 13495 | SAMN02673458 | 2.16443 | 52.8 | 1568 | GCF_000741765.1_Biftsu_genomic.fna |
| *Bifidobacterium tsurumiense* DSM 17777 | DSM 17777 | SAMN02440869 | 2.16654 | 52.9 | 1643 | GCF_000429745.1_ASM42974v1_genomic.fna |
| *Bifidobacterium tsurumiense* DSM 17777 | DSM 17777 | SAMN02442009 | 2.15769 | 52.8 | 1631 | GCF_000771065.1_DSM-17777_genomic.fna |

**Table S2 The information of sequences used in phylogenetic reconstruction of BSH genes**

| gene_ID after Prokka annoation | Tree_leaf_name | Sequence length(bp) |
|---|---|---|
| GCF_000020425.1_ASM2042v1_01534 | BSH-T4_Bifidobacterium_longum_subsp.infantis_ATCC_15697 | 954 |
| GCF_000154085.1_ASM15408v1_00676 | BSH-T4_Bifidobacterium_adolescentis_L2-32 | 951 |
| GCF_000155395.1_ASM15539v1_01343 | BSH-T4_Bifidobacterium_bifidum_NCIMB 41171 | 951 |
| GCF_000003135.1_ASM313v1_01131 | BSH-T4_Bifidobacterium_longum_subsp.longum_ATCC_55813 | 954 |
| GCF_000092765.1_ASM9276v1_00856 | BSH-T4_Bifidobacterium_animalis_subsp.lactis_V9 | 945 |
| GCF_000092325.1_ASM9232v1_00966 | BSH-T4_Bifidobacterium_longum_subsp.longum_JDM301 | 954 |
| GCF_000155415.1_ASM15541v1_01368 | BSH-T4_Bifidobacterium_longum_subsp.infantis_CCUG_52486 | 954 |
| GCF_000158015.1_ASM15801v1_01810 | BSH-T4_Bifidobacterium_breve_DSM_20213=JCM_1192 | 954 |
| GCF_000213865.1_ASM21386v1_00885 | gene2_breve_cbh | 954 |
| GCF_002838705.1_ASM283870v1_01187 | gene3_breve_cbh | 954 |
| GCF_004333385.1_ASM433338v1_01029 | gene4_longum_cbh | 954 |
| GCF_003471385.1_ASM347138v1_01646 | gene5_longum_cbh | 954 |
| GCF_003094895.1_ASM309489v1_00267 | gene6_animalis_cbh | 945 |
| GCF_002076025.1_Bbif1888B_00643 | gene7_longum_cbh | 954 |
| GCF_000708005.1_B.p.IPLA36007_01076 | gene8_pseudocatenulatum_cbh | 951 |
| GCF_001546275.1_ASM154627v1_01274 | gene9_longum_cbh | 954 |
| GCF_003094775.1_ASM309477v1_00268 | gene10_animalis_cbh | 945 |
| GCF_001595435.1_ASM159543v1_01315 | gene11_bifidum_cbh | 951 |
| GCF_001686145.1_ASM168614v1_00435 | gene12_longum_cbh | 954 |
| GCF_003437325.1_ASM343732v1_01767 | gene13_bifidum_cbh | 951 |
| GCF_001686185.1_ASM168618v1_00585 | gene14_longum_cbh | 954 |
| GCF_003094875.1_ASM309487v1_00205 | gene15_animalis_cbh | 945 |
| GCF_000466525.1_ASM46652v1_01831 | gene16_bifidum_cbh | 951 |
| GCF_000741685.1_Bifpsetum_00413 | gene17_pseudocatenulatum_cbh | 951 |
| GCF_004333175.1_ASM433317v1_00908 | gene18_longum_cbh | 954 |
| GCF_003465545.1_ASM346554v1_00470 | gene19_longum_cbh | 954 |

| | | |
|---|---|---|
| GCF_002914865.1_ASM291486v1_00859 | gene20_breve_cbh | 954 |
| GCF_000008945.1_ASM894v1_00542 | gene21_longum_cbh | 954 |
| GCF_001686045.1_ASM168604v1_00991 | gene22_pseudocatenulatum_cbh | 951 |
| GCF_001685985.1_ASM168598v1_01691 | gene23_pseudocatenulatum_cbh | 951 |
| GCF_002833175.1_ASM283317v1_00873 | gene24_longum_cbh | 954 |
| GCF_003458965.1_ASM345896v1_00380 | gene25_pseudocatenulatum_cbh | 951 |
| GCF_004333785.1_ASM433378v1_00985 | gene26_longum_cbh | 954 |
| GCF_000825025.1_BIC1307292462.V1_01591 | gene27_longum_cbh | 954 |
| GCF_000771285.1_DSM-20211_00261 | gene28_longum_cbh | 954 |
| GCF_002838465.1_ASM283846v1_00986 | gene29_breve_cbh | 954 |
| GCF_002846775.1_ASM284677v1_01664 | gene30_pseudolongum_cbh | 945 |
| GCF_004155155.1_ASM415515v1_01629 | gene31_pseudolongum_cbh | 945 |
| GCF_000730135.1_ASM73013v1_01262 | gene32_longum_cbh | 954 |
| GCF_004155705.1_ASM415570v1_01215 | gene33_pseudolongum_cbh | 945 |
| GCF_003471245.1_ASM347124v1_01561 | gene34_longum_cbh | 954 |
| GCF_001264155.1_ASM126415v1_00297 | gene35_breve_cbh | 954 |
| GCA_003573955.1_ASM357395v1_01796 | gene36_bifidum_cbh | 951 |
| GCF_900105745.1_IMG-taxon_2634166294_annotated_assembly_00315 | gene37_dentium_cbh | 951 |
| GCF_000771705.1_JCM-15918_01091 | gene38_adolescentis_cbh | 951 |
| GCF_004156085.1_ASM415608v1_00978 | gene39_pseudolongum_cbh | 945 |
| GCF_002271275.1_Bbre7Ev1_01597 | gene40_breve_cbh | 954 |
| GCF_900519135.1_BB-79_00175 | gene41_longum_cbh | 954 |
| GCF_000741085.1_Bifbif_01391 | gene42_bifidum_cbh | 951 |
| GCF_001686125.1_ASM168612v1_00303 | gene43_longum_cbh | 954 |
| GCF_003437435.1_ASM343743v1_00261 | gene44_pseudocatenulatum_cbh | 951 |
| GCF_003465385.1_ASM346538v1_01137 | gene45_pseudocatenulatum_cbh | 951 |
| GCF_002838305.1_ASM283830v1_00993 | gene46_breve_cbh | 954 |
| GCF_004333855.1_ASM433385v1_00973 | gene47_longum_cbh | 954 |

| | | |
|---|---|---|
| GCF_002838485.1_ASM283848v1_00984 | gene48_breve_cbh | 954 |
| GCF_000220885.1_ASM22088v1_00858 | gene49_animalis_cbh | 945 |
| GCF_003472405.1_ASM347240v1_01159 | gene50_longum_cbh | 954 |
| GCF_004333555.1_ASM433355v1_00909 | gene51_longum_cbh | 954 |
| GCF_004333215.1_ASM433321v1_00977 | gene52_longum_cbh | 954 |
| GCF_004156115.1_ASM415611v1_01211 | gene53_pseudolongum_cbh | 945 |
| GCF_003369995.1_ASM336999v1_00956 | gene54_longum_cbh | 954 |
| GCF_003438565.1_ASM343856v1_00713 | gene55_animalis_cbh | 945 |
| GCF_000471945.1_ASM47194v1_00903 | gene56_animalis_cbh | 945 |
| GCF_001020415.1_Bbif09v4_01394 | gene57_bifidum_cbh | 951 |
| GCF_004154645.1_ASM415464v1_00052 | gene58_animalis_cbh | 945 |
| GCF_004334625.1_ASM433462v1_01037 | gene59_longum_cbh | 954 |
| GCF_002838565.1_ASM283856v1_01090 | gene60_breve_cbh | 954 |
| GCF_004155135.1_ASM415513v1_01814 | gene61_pseudolongum_cbh | 945 |
| GCF_004334635.1_ASM433463v1_00938 | gene62_longum_cbh | 954 |
| GCF_001446255.1_ASM144625v1_00848 | gene63_longum_cbh | 954 |
| GCF_000771485.1_DSM-20456_01070 | gene64_bifidum_cbh | 951 |
| GCF_001020355.1_Bbif01v4_01617 | gene65_bifidum_cbh | 951 |
| GCF_002846725.1_ASM284672v1_00386 | gene66_pseudolongum_cbh | 945 |
| GCF_000210755.1_ASM21075v1_01006 | gene67_longum_cbh | 954 |
| GCF_900157055.1_Bifido_09_v1_02090 | gene68_longum_cbh | 954 |
| GCF_001719085.1_ASM171908v1_01034 | gene69_longum_cbh | 954 |
| GCF_004334855.1_ASM433485v1_01189 | gene70_longum_cbh | 954 |
| GCF_002282915.1_ASM228291v1_00888 | gene71_pseudolongum_cbh | 945 |
| GCF_002108155.1_BadoLMG11579v1_00956 | gene72_adolescentis_cbh | 951 |
| GCF_001685925.1_ASM168592v1_01330 | gene73_breve_cbh | 954 |
| GCF_003472685.1_ASM347268v1_00182 | gene74_pseudocatenulatum_cbh | 951 |
| GCF_004155405.1_ASM415540v1_01094 | gene75_pseudolongum_cbh | 945 |
| GCF_004154535.1_ASM415453v1_00901 | gene76_animalis_cbh | 945 |

| | | |
|---|---|---|
| GCF_004155835.1_ASM415583v1_00921 | gene77_pseudolongum_cbh | 945 |
| GCF_004333065.1_ASM433306v1_00917 | gene78_longum_cbh | 954 |
| GCF_003370225.1_ASM337022v1_00173 | gene79_longum_cbh | 954 |
| GCF_002833075.1_ASM283307v1_00865 | gene80_longum_cbh | 954 |
| GCF_002075955.1_Bbif1893B_00594 | gene81_dentium_cbh | 951 |
| GCF_000786175.1_ASM78617v1_01140 | gene82_longum_cbh | 954 |
| GCF_000687595.1_ASM68759v1_00101 | gene83_pseudolongum_cbh | 945 |
| GCF_003439655.1_ASM343965v1_00873 | gene84_pseudocatenulatum_cbh | 951 |
| GCF_003436955.1_ASM343695v1_00478 | gene85_pseudocatenulatum_cbh | 951 |
| GCF_001263845.1_ASM126384v1_00942 | gene86_breve_cbh | 954 |
| GCF_003457765.1_ASM345776v1_00961 | gene87_adolescentis_cbh | 951 |
| GCF_002833265.1_ASM283326v1_00916 | gene88_longum_cbh | 954 |
| GCF_004334715.1_ASM433471v1_00998 | gene89_longum_cbh | 954 |
| GCF_003473105.1_ASM347310v1_00933 | gene90_adolescentis_cbh | 951 |
| GCF_004334515.1_ASM433451v1_00967 | gene91_longum_cbh | 954 |
| GCF_001685865.1_ASM168586v1_01161 | gene92_breve_cbh | 954 |
| GCF_001039715.1_BAN2_01181 | gene93_animalis_cbh | 945 |
| GCF_004154555.1_ASM415455v1_00861 | gene94_animalis_cbh | 945 |
| GCF_004799295.1_ASM479929v1_01479 | gene95_bifidum_cbh | 951 |
| GCF_002076055.1_Bbif1891B_00902 | gene96_breve_cbh | 954 |
| GCF_002838245.1_ASM283824v1_00938 | gene97_breve_cbh | 954 |
| GCF_000226175.1_ASM22617v2_00349 | gene98_breve_cbh | 954 |
| GCF_002846755.1_ASM284675v1_01235 | gene99_pseudolongum_cbh | 945 |
| GCF_003094835.1_ASM309483v1_00058 | gene100_animalis_cbh | 945 |
| GCF_000224965.2_ASM22496v2_00857 | gene101_animalis_cbh | 945 |
| GCF_004333925.1_ASM433392v1_01065 | gene102_longum_cbh | 954 |
| GCF_000730035.1_ASM73003v1_01770 | gene103_longum_cbh | 954 |
| GCF_003370035.1_ASM337003v1_00283 | gene104_longum_cbh | 954 |
| GCF_003436655.1_ASM343665v1_01328 | gene105_bifidum_cbh | 951 |

| | | |
|---|---|---|
| GCF_000414215.1_ASM41421v1_00859 | gene106_animalis_cbh | 945 |
| GCF_001020325.1_Bbif10v4_01344 | gene107_bifidum_cbh | 951 |
| GCF_004155295.1_ASM415529v1_01134 | gene108_pseudolongum_cbh | 945 |
| GCF_002220485.1_ASM222048v1_00860 | gene109_animalis_cbh | 945 |
| GCF_001685705.1_ASM168570v1_00724 | gene110_breve_cbh | 954 |
| GCF_002276185.1_ASM227618v1_01566 | gene111_longum_cbh | 954 |
| GCF_000772485.1_ASM77248v1_00997 | gene112_longum_cbh | 954 |
| GCF_002075945.1_Bbif1896B_00380 | gene113_pseudocatenulatum_cbh | 951 |
| GCF_003095035.1_ASM309503v1_01061 | gene114_bifidum_cbh | 951 |
| GCF_002075935.1_Bbif1897B_01326 | gene115_longum_cbh | 954 |
| GCF_004333735.1_ASM433373v1_01267 | gene116_longum_cbh | 954 |
| GCF_002846875.1_ASM284687v1_00560 | gene117_pseudolongum_cbh | 945 |
| GCF_000021425.1_ASM2142v1_01453 | gene118_animalis_cbh | 945 |
| GCF_000522505.1_DSM27321_01002 | gene119_moukalabense_cbh | 951 |
| GCF_002838405.1_ASM283840v1_00946 | gene120_breve_cbh | 954 |
| GCF_003467905.1_ASM346790v1_00323 | gene121_longum_cbh | 954 |
| GCF_004333675.1_ASM433367v1_00882 | gene122_longum_cbh | 954 |
| GCF_003970855.1_ASM397085v1_00912 | gene123_animalis_cbh | 945 |
| GCF_900157065.1_PC1_v1_01688 | gene124_longum_cbh | 954 |
| GCF_000817045.1_ASM81704v1_00859 | gene125_animalis_cbh | 945 |
| GCF_000568975.1_ASM56897v1_01046 | gene127_breve_cbh | 954 |
| GCF_002838285.1_ASM283828v1_00963 | gene128_breve_cbh | 954 |
| GCF_001685785.1_ASM168578v1_01106 | gene129_breve_cbh | 954 |
| GCF_003436575.1_ASM343657v1_00338 | gene130_bifidum_cbh | 951 |
| GCF_003472725.1_ASM347272v1_00918 | gene131_pseudocatenulatum_cbh | 951 |
| GCF_001189355.1_assBREI4_01110 | gene132_breve_cbh | 954 |
| GCF_900157185.1_PC4_v1_02285 | gene133_longum_cbh | 954 |
| GCF_003436595.1_ASM343659v1_00665 | gene134_longum_cbh | 954 |
| GCF_001685905.1_ASM168590v1_00064 | gene135_breve_cbh | 954 |

| | | |
|---|---|---|
| GCF_000277345.1_ASM27734v1_00857 | gene136_animalis_cbh | 945 |
| GCF_004334615.1_ASM433461v1_00970 | gene137_longum_cbh | 954 |
| GCF_000410595.1_ASM41059v1_00558 | gene138_longum_cbh | 954 |
| GCF_001756865.1_ASM175686v1_00668 | gene139_adolescentis_cbh | 951 |
| GCF_001686225.1_ASM168622v1_00331 | gene140_longum_cbh | 954 |
| GCF_000569015.1_ASM56901v1_01072 | gene141_breve_cbh | 954 |
| GCF_001685825.1_ASM168582v1_01753 | gene142_breve_cbh | 954 |
| GCF_004334065.1_ASM433406v1_01154 | gene143_longum_cbh | 954 |
| GCF_004156135.1_ASM415613v1_00433 | gene144_pseudolongum_cbh | 945 |
| GCF_000825045.1_BIC1401111250.V1_01587 | gene145_longum_cbh | 954 |
| GCF_003094635.1_ASM309463v1_01638 | gene146_longum_cbh | 954 |
| GCF_005844365.1_ASM584436v1_00578 | gene147_bifidum_cbh | 951 |
| GCF_003094995.1_ASM309499v1_00603 | gene148_longum_cbh | 954 |
| GCF_003471355.1_ASM347135v1_01294 | gene149_longum_cbh | 954 |
| GCF_003573895.1_ASM357389v1_00220 | gene150_bifidum_cbh | 951 |
| GCF_000299595.1_ASM29959v1_01017 | gene151_bifidum_cbh | 951 |
| GCF_003095075.1_ASM309507v1_00205 | gene152_animalis_cbh | 945 |
| GCF_005405705.1_ASM540570v1_00014 | gene153_moukalabense_cbh | 951 |
| GCF_004125375.1_ASM412537v1_01602 | gene154_longum_cbh | 954 |
| GCF_002107995.1_BadoLMG10734v1_01056 | gene155_adolescentis_cbh | 951 |
| GCF_003475835.1_ASM347583v1_00879 | gene156_longum_cbh | 954 |
| GCF_002107925.1_Bado42Bv1_01134 | gene157_adolescentis_cbh | 951 |
| GCF_001263915.1_ASM126391v1_01374 | gene158_breve_cbh | 954 |
| GCF_000741125.1_Bifbre_01806 | gene159_breve_cbh | 954 |
| GCF_004332755.1_ASM433275v1_00950 | gene160_longum_cbh | 954 |
| GCF_000166895.2_ASM16689v2_01779 | gene161_longum_cbh | 954 |
| GCF_004156175.1_ASM415617v1_00554 | gene162_pseudolongum_cbh | 945 |
| GCF_004155635.1_ASM415563v1_01092 | gene163_pseudolongum_cbh | 945 |
| GCF_003473205.1_ASM347320v1_01122 | gene164_longum_cbh | 954 |

| | | |
|---|---|---|
| GCF_004334255.1_ASM433425v1_00853 | gene165_longum_cbh | 954 |
| GCF_004167735.1_ASM416773v1_00102 | gene166_dentium_cbh | 951 |
| GCF_004155495.1_ASM415549v1_00974 | gene167_pseudolongum_cbh | 945 |
| GCF_001516925.1_ASM151692v1_00302 | gene168_longum_cbh | 954 |
| GCF_005845205.1_ASM584520v1_00739 | gene169_adolescentis_cbh | 951 |
| GCF_003474015.1_ASM347401v1_00473 | gene170_pseudocatenulatum_cbh | 951 |
| GCF_005405665.1_ASM540566v1_00014 | gene171_moukalabense_cbh | 951 |
| GCF_003370205.1_ASM337020v1_01838 | gene172_longum_cbh | 1122 |
| GCF_000737885.1_ASM73788v1_00958 | gene173_adolescentis_cbh | 951 |
| GCF_001311295.1_ASM131129v1_02819 | gene174_breve_cbh | 954 |
| GCF_003466505.1_ASM346650v1_00543 | gene175_longum_cbh | 954 |
| GCF_002108075.1_BadoAL462v1_01241 | gene176_adolescentis_cbh | 951 |
| GCF_001406215.1_13470_2_69_00145 | gene177_adolescentis_cbh | 951 |
| GCF_003467395.1_ASM346739v1_01045 | gene178_longum_cbh | 954 |
| GCF_002271255.1_Bpse1Ev1_00420 | gene179_pseudocatenulatum_cbh | 951 |
| GCF_004167585.1_ASM416758v1_00413 | gene180_adolescentis_cbh | 951 |
| GCF_004154525.1_ASM415452v1_00895 | gene181_animalis_cbh | 945 |
| GCF_004154565.1_ASM415456v1_00900 | gene182_animalis_cbh | 945 |
| GCF_001020275.1_Bbif07v4_01535 | gene183_bifidum_cbh | 951 |
| GCF_900157145.1_Bifido_03_v1_01699 | gene184_longum_cbh | 954 |
| GCF_003470585.1_ASM347058v1_01837 | gene185_bifidum_cbh | 951 |
| GCF_004333905.1_ASM433390v1_00925 | gene186_longum_cbh | 954 |
| GCF_900112465.1_IMG-taxon_2654588208_annotated_assembly_01196 | gene187_bifidum_cbh | 951 |
| GCF_002833215.1_ASM283321v1_00877 | gene188_longum_cbh | 954 |
| GCF_004332935.1_ASM433293v1_00856 | gene189_longum_cbh | 954 |
| GCF_002833255.1_ASM283325v1_00884 | gene190_longum_cbh | 954 |
| GCF_005844245.1_ASM584424v1_01250 | gene191_animalis_cbh | 945 |
| GCF_002832995.1_ASM283299v1_00927 | gene192_longum_cbh | 954 |

| | | |
|---|---|---|
| GCF_004154435.1_ASM415443v1_00861 | gene193_animalis_cbh | 945 |
| GCF_900157085.1_Bifido_04_v1_01008 | gene194_longum_cbh_1 | 1023 |
| GCF_000569035.1_ASM56903v1_00958 | gene195_breve_cbh | 954 |
| GCF_004154655.1_ASM415465v1_00906 | gene196_animalis_cbh | 945 |
| GCF_000024445.1_ASM2444v1_01089 | gene197_dentium_cbh | 951 |
| GCF_003467065.1_ASM346706v1_00631 | gene198_pseudocatenulatum_cbh | 951 |
| GCF_002833015.1_ASM283301v1_00811 | gene199_longum_cbh | 954 |
| GCF_000497735.1_BLONGv1.0_01002 | gene200_longum_cbh | 954 |
| GCF_003370105.1_ASM337010v1_01890 | gene201_breve_cbh | 954 |
| GCF_005405805.1_ASM540580v1_00338 | gene202_moukalabense_cbh | 951 |
| GCF_003438485.1_ASM343848v1_00042 | gene203_longum_cbh | 954 |
| GCF_004334535.1_ASM433453v1_00917 | gene204_longum_cbh | 954 |
| GCF_001281345.1_ASM128134v1_00896 | gene205_bifidum_cbh | 951 |
| GCF_000025245.1_ASM2524v1_01358 | gene206_animalis_cbh | 945 |
| GCF_003437825.1_ASM343782v1_00501 | gene207_pseudocatenulatum_cbh | 951 |
| GCF_900157085.1_Bifido_04_v1_02183 | gene208_longum_cbh | 954 |
| GCF_004332825.1_ASM433282v1_00859 | gene209_longum_cbh | 954 |
| GCF_003462885.1_ASM346288v1_00958 | gene210_adolescentis_cbh | 951 |
| GCF_004155525.1_ASM415552v1_00969 | gene211_pseudolongum_cbh | 945 |
| GCF_900157125.1_Bifido_07_v1_00402 | gene212_breve_cbh | 954 |
| GCF_004333035.1_ASM433303v1_01153 | gene213_longum_cbh | 954 |
| GCF_002838345.1_ASM283834v1_00996 | gene214_breve_cbh | 954 |
| GCF_000825145.1_BIB1401272845b.V1_01570 | gene215_longum_cbh | 954 |
| GCF_000612705.1_CECT8145_00714 | gene216_animalis_cbh | 945 |
| GCF_004154545.1_ASM415454v1_00966 | gene217_animalis_cbh | 945 |
| GCF_003437715.1_ASM343771v1_00373 | gene218_longum_cbh | 954 |
| GCF_004333265.1_ASM433326v1_00963 | gene219_longum_cbh | 954 |
| GCF_001940535.1_BlonW11v1_01159 | gene220_longum_cbh | 954 |
| GCF_000164965.1_ASM16496v1_00902 | gene221_bifidum_cbh | 951 |

| | | |
|---|---|---|
| GCF_004334645.1_ASM433464v1_00980 | gene222_longum_cbh | 954 |
| GCF_001686105.1_ASM168610v1_01446 | gene223_longum_cbh | 954 |
| GCF_000569075.1_ASM56907v1_00982 | gene224_breve_cbh | 954 |
| GCF_004333475.1_ASM433347v1_00920 | gene225_longum_cbh | 954 |
| GCF_900445615.1_49964_F01_00169 | gene226_adolescentis_cbh | 951 |
| GCF_003370005.1_ASM337000v1_02031 | gene227_longum_cbh | 954 |
| GCF_900157105.1_Bifido_10_v1_00991 | gene228_breve_cbh | 954 |
| GCF_002833205.1_ASM283320v1_00870 | gene229_longum_cbh | 954 |
| GCF_003469145.1_ASM346914v1_00502 | gene230_adolescentis_cbh | 951 |
| GCF_004154475.1_ASM415447v1_00899 | gene232_animalis_cbh | 945 |
| GCF_002706665.1_ASM270666v1_00981 | gene233_pseudolongum_cbh | 945 |
| GCF_003463425.1_ASM346342v1_00156 | gene234_pseudocatenulatum_cbh | 951 |
| GCF_005405785.1_ASM540578v1_00987 | gene235_moukalabense_cbh | 951 |
| GCF_002833035.1_ASM283303v1_00916 | gene236_longum_cbh | 954 |
| GCF_004333895.1_ASM433389v1_01009 | gene237_longum_cbh | 954 |
| GCF_005405685.1_ASM540568v1_00237 | gene238_moukalabense_cbh | 951 |
| GCF_004333455.1_ASM433345v1_00928 | gene239_longum_cbh | 954 |
| GCF_002914895.1_ASM291489v1_00105 | gene240_animalis_cbh | 945 |
| GCF_003370195.1_ASM337019v1_02061 | gene241_breve_cbh | 954 |
| GCF_000240765.1_BifAniBS01_1.0_00903 | gene242_animalis_cbh | 945 |
| GCF_003472345.1_ASM347234v1_00243 | gene243_bifidum_cbh | 951 |
| GCF_000300215.1_ASM30021v1_00072 | gene244_bifidum_cbh | 951 |
| GCF_005405645.1_ASM540564v1_00014 | gene245_moukalabense_cbh | 951 |
| GCF_002846845.1_ASM284684v1_01404 | gene246_pseudolongum_cbh | 945 |
| GCF_000771105.1_DSM-20088_00685 | gene247_longum_cbh | 954 |
| GCF_001050555.1_ASM105055v1_01078 | gene248_longum_cbh | 954 |
| GCF_900104835.1_IMG-taxon_2634166334_annotated_assembly_01147 | gene249_longum_cbh | 954 |
| GCF_003471415.1_ASM347141v1_00463 | gene250_pseudocatenulatum_cbh | 951 |

| | | |
|---|---|---|
| GCF_000261245.1_Blongum16Bv1.0_01873 | gene251_longum_cbh | 954 |
| GCF_001447975.1_ASM144797v1_00008 | gene252_longum_cbh | 954 |
| GCF_004319685.1_ASM431968v1_01059 | gene253_breve_cbh | 954 |
| GCF_001263945.1_ASM126394v1_00941 | gene254_animalis_cbh | 945 |
| GCF_004333335.1_ASM433333v1_00940 | gene255_longum_cbh | 954 |
| GCF_004154455.1_ASM415445v1_00758 | gene256_animalis_cbh | 945 |
| GCF_003475865.1_ASM347586v1_00366 | gene257_longum_cbh | 954 |
| GCF_002838605.1_ASM283860v1_00997 | gene258_breve_cbh | 954 |
| GCF_003458335.1_ASM345833v1_01918 | gene259_longum_cbh | 954 |
| GCF_000022965.1_ASM2296v1_00859 | gene260_animalis_cbh | 945 |
| GCF_004334425.1_ASM433442v1_00901 | gene261_longum_cbh | 954 |
| GCF_003466485.1_ASM346648v1_00874 | gene262_bifidum_cbh | 951 |
| GCF_000730055.1_ASM73005v1_00729 | gene263_longum_cbh | 954 |
| GCF_004155375.1_ASM415537v1_00983 | gene264_pseudolongum_cbh | 945 |
| GCF_003437785.1_ASM343778v1_00875 | gene265_bifidum_cbh | 951 |
| GCF_005405845.1_ASM540584v1_00987 | gene266_moukalabense_cbh | 951 |
| GCF_001446275.1_ASM144627v1_00888 | gene267_longum_cbh | 954 |
| GCF_003094715.1_ASM309471v1_01219 | gene268_bifidum_cbh | 951 |
| GCF_001870705.1_ASM187070v1_01160 | gene269_longum_cbh | 954 |
| GCA_003473345.1_ASM347334v1_01130 | gene270_adolescentis_cbh | 951 |
| GCF_000260715.1_ASM26071v1_00880 | gene271_animalis_cbh | 945 |
| GCF_004167725.1_ASM416772v1_01547 | gene272_longum_cbh | 954 |
| GCF_003474045.1_ASM347404v1_00675 | gene273_bifidum_cbh | 951 |
| GCF_001688645.2_ASM168864v2_01421 | gene274_animalis_cbh | 945 |
| GCF_003370265.1_ASM337026v1_01802 | gene275_breve_cbh | 954 |
| GCF_004802595.1_ASM480259v1_01328 | gene276_breve_cbh | 954 |
| GCF_003465775.1_ASM346577v1_01255 | gene278_pseudocatenulatum_cbh | 951 |
| GCF_001546235.1_ASM154623v1_01989 | gene279_breve_cbh | 954 |
| GCF_004332665.1_ASM433266v1_00901 | gene280_longum_cbh | 954 |

| | | |
|---|---|---|
| GCF_003475055.1_ASM347505v1_01592 | gene281_longum_cbh | 954 |
| GCF_000730025.1_ASM73002v1_01624 | gene282_longum_cbh | 954 |
| GCF_001686165.1_ASM168616v1_01275 | gene283_longum_cbh | 954 |
| GCF_002838425.1_ASM283842v1_01046 | gene284_breve_cbh | 954 |
| GCF_003471915.1_ASM347191v1_00592 | gene285_longum_cbh | 954 |
| GCF_004332735.1_ASM433273v1_01018 | gene286_longum_cbh | 954 |
| GCF_003466395.1_ASM346639v1_00935 | gene287_bifidum_cbh | 951 |
| GCF_001263855.1_ASM126385v1_01883 | gene288_breve_cbh | 954 |
| GCF_003790375.1_ASM379037v1_01203 | gene289_longum_cbh | 954 |
| GCF_004334445.1_ASM433444v1_00829 | gene290_longum_cbh | 954 |
| GCF_004334165.1_ASM433416v1_01091 | gene291_longum_cbh | 954 |
| GCF_003094955.1_ASM309495v1_01706 | gene292_longum_cbh | 954 |
| GCF_005844115.1_ASM584411v1_01667 | gene293_longum_cbh | 954 |
| GCF_000741325.1_Bifpse_sub.pse_00532 | gene294_pseudolongum_cbh | 945 |
| GCF_005406285.1_ASM540628v1_00863 | gene295_longum_cbh | 954 |
| GCF_004155435.1_ASM415543v1_00164 | gene296_pseudolongum_cbh | 945 |
| GCF_004332945.1_ASM433294v1_01093 | gene297_longum_cbh | 954 |
| GCF_005844205.1_ASM584420v1_00725 | gene298_bifidum_cbh | 951 |
| GCF_003459865.1_ASM345986v1_00932 | gene299_pseudocatenulatum_cbh | 951 |
| GCF_000818055.1_ASM81805v1_00858 | gene300_animalis_cbh | 945 |
| GCF_004156215.1_ASM415621v1_01026 | gene301_pseudolongum_cbh | 945 |
| GCF_001020405.1_Bbif06v4_01321 | gene302_bifidum_cbh | 951 |
| GCF_000173435.1_ASM17343v1_00683 | gene303_pseudocatenulatum_cbh | 951 |
| GCF_004683745.1_ASM468374v1_00604 | gene304_dentium_cbh | 951 |
| GCF_900157115.1_Bifido_05_v1_00745 | gene305_longum_cbh | 954 |
| GCF_004332835.1_ASM433283v1_00832 | gene306_longum_cbh | 954 |
| GCF_001025175.1_ASM102517v1_00932 | gene307_breve_cbh | 954 |
| GCF_003472605.1_ASM347260v1_00606 | gene308_longum_cbh | 954 |
| GCF_003095115.1_ASM309511v1_00268 | gene309_animalis_cbh | 945 |

| | | |
|---|---|---|
| GCF_001892965.1_ASM189296v1_00306 | gene310_longum_cbh | 954 |
| GCF_000817995.1_ASM81799v1_00999 | gene311_adolescentis_cbh | 951 |
| GCF_000220135.1_ASM22013v1_01061 | gene312_breve_cbh | 954 |
| GCF_900157075.1_Bifido_S1_v1_01762 | gene313_longum_cbh | 954 |
| GCF_001264035.1_ASM126403v1_00687 | gene314_breve_cbh | 954 |
| GCF_003471345.1_ASM347134v1_01471 | gene315_longum_cbh | 954 |
| GCF_002832945.1_ASM283294v1_00923 | gene316_longum_cbh | 954 |
| GCF_004135895.1_ASM413589v1_00858 | gene317_animalis_cbh | 945 |
| GCF_004334865.1_ASM433486v1_01034 | gene318_longum_cbh | 954 |
| GCF_004333275.1_ASM433327v1_00900 | gene319_longum_cbh | 954 |
| GCF_003856735.1_ASM385673v1_01016 | gene320_adolescentis_cbh | 951 |
| GCF_004333165.1_ASM433316v1_00885 | gene321_longum_cbh | 954 |
| GCF_004334105.1_ASM433410v1_01063 | gene322_longum_cbh | 954 |
| GCF_004155285.1_ASM415528v1_00336 | gene323_pseudolongum_cbh | 945 |
| GCF_004332895.1_ASM433289v1_01287 | gene324_longum_cbh | 954 |
| GCF_000569055.1_ASM56905v1_01001 | gene325_breve_cbh | 954 |
| GCF_003437135.1_ASM343713v1_00176 | gene326_longum_cbh | 954 |
| GCF_001264055.1_ASM126405v1_00778 | gene327_animalis_cbh | 945 |
| GCF_003467515.1_ASM346751v1_01205 | gene328_pseudocatenulatum_cbh | 951 |
| GCF_003437945.1_ASM343794v1_00965 | gene329_bifidum_cbh | 951 |
| GCF_003467985.1_ASM346798v1_00227 | gene330_bifidum_cbh | 951 |
| GCF_000478525.1_blongD2957_01382 | gene331_longum_cbh | 954 |
| GCF_900157135.1_Bifido_11_v1_01133 | gene332_animalis_cbh | 945 |
| GCF_002833065.1_ASM283306v1_00952 | gene333_longum_cbh | 954 |
| GCF_004154445.1_ASM415444v1_00819 | gene334_animalis_cbh | 945 |
| GCF_002803775.1_ASM280377v1_00691 | gene335_animalis_cbh | 945 |
| GCF_003468825.1_ASM346882v1_01707 | gene336_longum_cbh | 954 |
| GCF_004333765.1_ASM433376v1_00854 | gene337_longum_cbh | 954 |
| GCF_004334205.1_ASM433420v1_00877 | gene338_longum_cbh | 954 |

| | | |
|---|---|---|
| GCF_001293145.1_ASM129314v1_00939 | gene339_longum_cbh | 954 |
| GCF_002108095.1_BadoAD467v1_01075 | gene340_adolescentis_cbh | 951 |
| GCF_003472305.1_ASM347230v1_01622 | gene341_longum_cbh | 954 |
| GCF_003436635.1_ASM343663v1_01327 | gene342_bifidum_cbh | 951 |
| GCF_003094735.1_ASM309473v1_01695 | gene343_bifidum_cbh | 951 |
| GCF_003436315.1_ASM343631v1_00476 | gene344_pseudocatenulatum_cbh | 951 |
| GCF_004155395.1_ASM415539v1_00911 | gene345_pseudolongum_cbh | 945 |
| GCF_001406735.1_13414_6_46_00239 | gene346_adolescentis_cbh | 951 |
| GCF_004167565.1_ASM416756v1_00471 | gene347_pseudocatenulatum_cbh | 951 |
| GCF_002833285.1_ASM283328v1_00838 | gene348_longum_cbh | 954 |
| GCF_003464845.1_ASM346484v1_01086 | gene349_pseudolongum_cbh | 945 |
| GCF_000165905.1_ASM16590v1_00869 | gene350_bifidum_cbh | 951 |
| GCF_005405725.1_ASM540572v1_01512 | gene351_moukalabense_cbh | 951 |
| GCF_003472575.1_ASM347257v1_00517 | gene352_pseudocatenulatum_cbh | 951 |
| GCF_002838545.1_ASM283854v1_00973 | gene353_breve_cbh | 954 |
| GCF_001264095.1_ASM126409v1_00464 | gene354_breve_cbh | 954 |
| GCF_004334775.1_ASM433477v1_01088 | gene355_longum_cbh | 954 |
| GCF_003437105.1_ASM343710v1_00969 | gene356_longum_cbh | 954 |
| GCF_003436505.1_ASM343650v1_01880 | gene357_longum_cbh | 954 |
| GCF_000771305.1_DSM-20213_01580 | gene358_breve_cbh | 954 |
| GCF_002846685.1_ASM284668v1_01350 | gene359_pseudolongum_cbh | 945 |
| GCF_004333645.1_ASM433364v1_00845 | gene360_longum_cbh | 954 |
| GCF_001892925.1_ASM189292v1_01496 | gene361_animalis_cbh | 945 |
| GCF_002838225.1_ASM283822v1_01024 | gene362_breve_cbh | 954 |
| GCF_900157165.1_Bifido_12_v1_01552 | gene363_longum_cbh | 954 |
| GCF_004154625.1_ASM415462v1_00474 | gene364_animalis_cbh | 945 |
| GCF_004334555.1_ASM433455v1_00900 | gene365_longum_cbh | 954 |
| GCF_004334005.1_ASM433400v1_00910 | gene366_longum_cbh | 954 |
| GCF_003464575.1_ASM346457v1_00180 | gene367_longum_cbh | 954 |

| | | |
|---|---|---|
| GCF_003465425.1_ASM346542v1_01692 | gene368_bifidum_cbh | 951 |
| GCF_003437455.1_ASM343745v1_01474 | gene369_longum_cbh | 954 |
| GCF_000568875.1_Mira_de-novo_assembly_00857 | gene370_breve_cbh | 954 |
| GCF_000730205.1_ASM73020v1_00968 | gene371_longum_cbh | 954 |
| GCF_003094815.1_ASM309481v1_00205 | gene372_animalis_cbh | 945 |
| GCF_900637095.1_48450_G02_01392 | gene373_bifidum_cbh | 951 |
| GCF_003465785.1_ASM346578v1_00838 | gene374_longum_cbh | 954 |
| GCF_003813065.1_ASM381306v1_00941 | gene375_breve_cbh | 954 |
| GCF_003471595.1_ASM347159v1_01794 | gene376_bifidum_cbh | 951 |
| GCF_003462895.1_ASM346289v1_00960 | gene377_adolescentis_cbh | 951 |
| GCF_003465065.1_ASM346506v1_01050 | gene378_pseudocatenulatum_cbh | 951 |
| GCF_003437075.1_ASM343707v1_01854 | gene379_pseudocatenulatum_cbh | 951 |
| GCF_001025135.1_ASM102513v1_00880 | gene380_bifidum_cbh | 951 |
| GCF_004333425.1_ASM433342v1_00850 | gene381_longum_cbh | 954 |
| GCA_002762435.1_ASM276243v1_00968 | gene382_animalis_cbh | 945 |
| GCF_001685765.1_ASM168576v1_01300 | gene383_breve_cbh | 954 |
| GCF_002838265.1_ASM283826v1_00995 | gene384_breve_cbh | 954 |
| GCF_003437155.1_ASM343715v1_00102 | gene385_pseudocatenulatum_cbh | 951 |
| GCF_002846675.1_ASM284667v1_00145 | gene386_pseudolongum_cbh | 945 |
| GCA_001576885.1_ASM157688v1_00660 | gene387_pseudocatenulatum_cbh | 951 |
| GCF_004155805.1_ASM415580v1_00958 | gene388_pseudolongum_cbh | 945 |
| GCF_003472415.1_ASM347241v1_00229 | gene389_pseudocatenulatum_cbh | 951 |
| GCF_003370295.1_ASM337029v1_00373 | gene390_breve_cbh | 954 |
| GCF_000771225.1_DSM-20099_01152 | gene391_pseudolongum_cbh | 945 |
| GCF_002076075.1_Bbif1889B_00631 | gene392_breve_cbh | 954 |
| GCF_003474835.1_ASM347483v1_01789 | gene393_pseudocatenulatum_cbh | 951 |
| GCF_004333845.1_ASM433384v1_00909 | gene394_longum_cbh | 954 |
| GCF_004332745.1_ASM433274v1_00926 | gene395_longum_cbh | 954 |
| GCF_001264135.1_ASM126413v1_01272 | gene396_breve_cbh | 954 |

| | | |
|---|---|---|
| GCF_000261225.1_Blongum35Bv1.0_00190 | gene397_longum_cbh | 951 |
| GCF_004155095.1_ASM415509v1_00207 | gene398_pseudolongum_cbh | 945 |
| GCF_003606305.1_ASM360630v1_00857 | gene399_animalis_cbh | 945 |
| GCF_004155845.1_ASM415584v1_01235 | gene400_pseudolongum_cbh | 945 |
| GCF_004334795.1_ASM433479v1_00942 | gene401_longum_cbh | 954 |
| GCF_004332925.1_ASM433292v1_00853 | gene402_longum_cbh | 954 |
| GCA_003508295.1_ASM350829v1_01073 | gene403_dentium_cbh | 951 |
| GCF_003438405.1_ASM343840v1_00796 | gene404_pseudocatenulatum_cbh | 951 |
| GCF_003437175.1_ASM343717v1_00936 | gene405_bifidum_cbh | 951 |
| GCF_004334705.1_ASM433470v1_00942 | gene406_longum_cbh | 954 |
| GCF_003472545.1_ASM347254v1_01156 | gene407_pseudocatenulatum_cbh | 951 |
| GCF_000825065.1_BIC1401212621a.V1_01575 | gene408_longum_cbh | 954 |
| GCF_004334235.1_ASM433423v1_01021 | gene409_longum_cbh | 954 |
| GCF_003468415.1_ASM346841v1_00723 | gene410_bifidum_cbh | 951 |
| GCF_005405765.1_ASM540576v1_01122 | gene411_moukalabense_cbh | 951 |
| GCF_004333695.1_ASM433369v1_00877 | gene412_longum_cbh | 954 |
| GCF_000010425.1_ASM1042v1_00929 | gene413_adolescentis_cbh | 951 |
| GCF_004155015.1_ASM415501v1_01060 | gene414_pseudolongum_cbh | 945 |
| GCF_003465635.1_ASM346563v1_01705 | gene415_longum_cbh | 954 |
| GCF_003468555.1_ASM346855v1_00479 | gene416_pseudocatenulatum_cbh | 951 |
| GCF_001263985.1_ASM126398v1_00332 | gene417_animalis_cbh | 945 |
| GCF_004334465.1_ASM433446v1_00939 | gene418_longum_cbh | 954 |
| GCF_004334435.1_ASM433443v1_00961 | gene419_longum_cbh | 954 |
| GCF_004333975.1_ASM433397v1_00860 | gene420_longum_cbh | 954 |
| GCF_003095015.1_ASM309501v1_01321 | gene421_animalis_cbh | 945 |
| GCF_004333775.1_ASM433377v1_00880 | gene422_longum_cbh | 954 |
| GCF_004333465.1_ASM433346v1_00935 | gene423_longum_cbh | 954 |
| GCF_004155725.1_ASM415572v1_00913 | gene424_pseudolongum_cbh | 945 |
| GCF_900637175.1_49569_F01_01089 | gene425_dentium_cbh | 951 |

| | | |
|---|---|---|
| GCF_004333005.1_ASM433300v1_00927 | gene426_longum_cbh | 954 |
| GCF_004332965.1_ASM433296v1_00899 | gene427_longum_cbh | 954 |
| GCF_004334035.1_ASM433403v1_00960 | gene428_longum_cbh | 954 |
| GCF_001020265.1_Bbif05v4_01332 | gene429_bifidum_cbh | 951 |
| GCF_003458805.1_ASM345880v1_00875 | gene430_adolescentis_cbh | 951 |
| GCF_003437735.1_ASM343773v1_01693 | gene431_adolescentis_cbh | 951 |
| GCF_001051015.2_ASM105101v2_00576 | gene432_longum_cbh | 954 |
| GCF_003370125.1_ASM337012v1_01326 | gene433_breve_cbh | 954 |
| GCF_002861445.1_ASM286144v1_00765 | gene434_longum_cbh | 954 |
| GCF_002107955.1_Bado70Bv1_01125 | gene435_adolescentis_cbh | 951 |
| GCF_004332655.1_ASM433265v1_00927 | gene436_longum_cbh | 954 |
| GCF_003428375.1_ASM342837v1_00960 | gene437_animalis_cbh | 945 |
| GCF_900157095.1_Bifido_01_v1_01892 | gene438_longum_cbh | 954 |
| GCF_002833185.1_ASM283318v1_00829 | gene439_longum_cbh | 954 |
| GCF_004333875.1_ASM433387v1_00853 | gene440_longum_cbh | 954 |
| GCF_002838665.1_ASM283866v1_00997 | gene441_breve_cbh | 954 |
| GCF_004155305.1_ASM415530v1_00255 | gene442_pseudolongum_cbh | 945 |
| GCA_001576955.1_ASM157695v1_00385 | gene443_longum_cbh | 954 |
| GCF_004155145.1_ASM415514v1_00584 | gene444_pseudolongum_cbh | 945 |
| GCF_000185665.1_ASM18566v1_01575 | gene445_longum_cbh | 954 |
| GCF_002832985.1_ASM283298v1_01001 | gene446_longum_cbh | 954 |
| GCF_001025215.1_ASM102521v1_00937 | gene447_pseudocatenulatum_cbh | 951 |
| GCF_004333325.1_ASM433332v1_01157 | gene448_longum_cbh | 954 |
| GCF_000741245.1_Biflon_sub.lon_00850 | gene449_longum_cbh | 954 |
| GCF_002075865.1_Bbif1900B_00430 | gene450_breve_cbh | 954 |
| GCF_002108015.1_Bado487Bv1_01034 | gene451_adolescentis_cbh | 951 |
| GCF_003095055.1_ASM309505v1_00839 | gene452_animalis_cbh | 945 |
| GCF_004156235.1_ASM415623v1_00248 | gene453_pseudolongum_cbh | 945 |
| GCF_000277325.1_ASM27732v1_00859 | gene454_animalis_cbh | 945 |

| | | |
|---|---|---|
| GCF_001447955.1_ASM144795v1_00825 | gene455_longum_cbh | 954 |
| GCF_004333235.1_ASM433323v1_01097 | gene456_longum_cbh | 954 |
| GCF_000172135.1_ASM17213v1_01839 | gene457_dentium_cbh | 951 |
| GCF_000730105.1_ASM73010v1_01076 | gene458_longum_cbh | 954 |
| GCF_000146775.1_ASM14677v1_00706 | gene459_dentium_cbh | 951 |
| GCF_001686125.1_ASM168612v1_00931 | gene460_longum_cbh_1 | 1023 |
| GCF_003370095.1_ASM337009v1_01744 | gene461_longum_cbh | 954 |
| GCF_003472265.1_ASM347226v1_01063 | gene463_adolescentis_cbh | 951 |
| GCF_900102865.1_IMG-taxon_2651870312_annotated_assembly_00704 | gene465_breve_cbh | 954 |
| GCF_000149165.1_ASM14916v1_02018 | gene466_dentium_cbh | 951 |
| GCF_000166315.1_ASM16631v1_00548 | gene467_longum_cbh | 954 |
| GCF_003466045.1_ASM346604v1_00812 | gene468_longum_cbh | 954 |
| GCA_004154635.1_ASM415463v1_01325 | gene469_animalis_cbh | 945 |
| GCF_003475025.1_ASM347502v1_00929 | gene470_longum_cbh | 954 |
| GCF_003438385.1_ASM343838v1_01374 | gene471_longum_cbh | 954 |
| GCF_004155715.1_ASM415571v1_00920 | gene472_pseudolongum_cbh | 945 |
| GCF_003370255.1_ASM337025v1_00211 | gene473_longum_cbh | 954 |
| GCF_003860285.1_ASM386028v1_01021 | gene474_breve_cbh | 954 |
| GCF_003612445.1_ASM361244v1_00523 | gene475_pseudolongum_cbh | 945 |
| GCF_002838725.1_ASM283872v1_01038 | gene476_breve_cbh | 954 |
| GCF_004332865.1_ASM433286v1_00902 | gene477_longum_cbh | 954 |
| GCF_001686085.1_ASM168608v1_00662 | gene478_dentium_cbh | 951 |
| GCF_003457975.1_ASM345797v1_01022 | gene479_longum_cbh | 954 |
| GCF_004155535.1_ASM415553v1_00127 | gene480_pseudolongum_cbh | 945 |
| GCF_005405865.1_ASM540586v1_00311 | gene481_moukalabense_cbh | 951 |
| GCF_003468805.1_ASM346880v1_00538 | gene482_longum_cbh | 954 |
| GCF_000421365.1_ASM42136v1_00122 | gene483_pseudolongum_cbh | 945 |
| GCF_004168525.1_ASM416852v1_01250 | gene484_pseudolongum_cbh | 945 |

| | | |
|---|---|---|
| GCF_001870755.1_ASM187075v1_00677 | gene485_longum_cbh | 954 |
| GCF_002838325.1_ASM283832v1_00958 | gene486_breve_cbh | 954 |
| GCF_004167365.1_ASM416736v1_00484 | gene487_bifidum_cbh | 951 |
| GCF_001546225.1_ASM154622v1_01866 | gene488_bifidum_cbh | 951 |
| GCF_003130775.1_ASM313077v1_00142 | gene489_longum_cbh | 954 |
| GCF_004155745.1_ASM415574v1_01027 | gene490_pseudolongum_cbh | 945 |
| GCF_002114145.1_ASM211414v1_01345 | gene491_bifidum_cbh | 951 |
| GCF_001405035.1_13470_2_59_01084 | gene492_pseudocatenulatum_cbh | 951 |
| GCF_002900845.1_ASM290084v1_00576 | gene493_longum_cbh | 954 |
| GCF_004333375.1_ASM433337v1_00926 | gene494_longum_cbh | 954 |
| GCF_900637335.1_50618_H02_00851 | gene495_longum_cbh | 954 |
| GCF_004334215.1_ASM433421v1_00883 | gene496_longum_cbh | 954 |
| GCF_004332765.1_ASM433276v1_01089 | gene497_longum_cbh | 954 |
| GCF_003436025.1_ASM343602v1_00461 | gene498_pseudocatenulatum_cbh | 951 |
| GCF_000741295.1_Bifpse_sub.glo_00833 | gene499_pseudolongum_cbh | 945 |
| GCF_001405355.1_13414_6_61_00313 | gene500_bifidum_cbh | 951 |
| GCF_004333045.1_ASM433304v1_00938 | gene501_longum_cbh | 954 |
| GCF_003437695.1_ASM343769v1_00564 | gene502_longum_cbh | 954 |
| GCF_003790385.1_ASM379038v1_01405 | gene503_bifidum_cbh | 951 |
| GCF_004155115.1_ASM415511v1_01381 | gene504_pseudolongum_cbh | 945 |
| GCF_004155565.1_ASM415556v1_01140 | gene505_pseudolongum_cbh | 945 |
| GCF_002846835.1_ASM284683v1_00832 | gene506_pseudolongum_cbh | 945 |
| GCF_003464805.1_ASM346480v1_00372 | gene507_longum_cbh | 954 |
| GCF_003436185.1_ASM343618v1_00770 | gene508_adolescentis_cbh | 951 |
| GCF_000829295.1_ASM82929v1_00863 | gene509_longum_cbh | 954 |
| GCF_002107975.1_BadoAL124v1_00973 | gene510_adolescentis_cbh | 951 |
| GCF_000695895.1_ASM69589v1_00858 | gene511_animalis_cbh | 945 |
| GCF_003472245.1_ASM347224v1_01810 | gene512_adolescentis_cbh | 951 |
| GCF_004334145.1_ASM433414v1_00899 | gene513_longum_cbh | 954 |

| | | |
|---|---|---|
| GCF_004155325.1_ASM415532v1_01549 | gene514_pseudolongum_cbh | 945 |
| GCF_002838385.1_ASM283838v1_00994 | gene515_breve_cbh | 954 |
| GCF_004154425.1_ASM415442v1_00225 | gene516_animalis_cbh | 945 |
| GCF_001275745.1_assBLOI2_00575 | gene517_longum_cbh | 954 |
| GCF_000196575.1_ASM19657v1_00983 | gene518_longum_cbh | 954 |
| GCF_003370065.1_ASM337006v1_01918 | gene519_longum_cbh | 954 |
| GCF_004332635.1_ASM433263v1_01011 | gene520_longum_cbh | 954 |
| GCF_004334335.1_ASM433433v1_01153 | gene521_longum_cbh | 954 |
| GCF_005405745.1_ASM540574v1_01410 | gene522_moukalabense_cbh | 951 |
| GCF_003429385.1_ASM342938v1_00940 | gene523_adolescentis_cbh | 951 |
| GCF_000568895.1_Mira_de-novo_assembly_01281 | gene524_breve_cbh | 954 |
| GCF_004334325.1_ASM433432v1_00859 | gene525_longum_cbh | 954 |
| GCF_900157045.1_Bifido_08_v1_01355 | gene526_animalis_cbh | 945 |
| GCF_002833125.1_ASM283312v1_00864 | gene527_longum_cbh | 954 |
| GCF_002833115.1_ASM283311v1_00861 | gene528_longum_cbh | 954 |
| GCF_000825005.1_BIC1206122787.V1_01580 | gene529_longum_cbh | 954 |
| GCF_003474905.1_ASM347490v1_01325 | gene530_longum_cbh | 954 |
| GCF_000261265.1_Blongum44Bv1.0_01178 | gene531_longum_cbh | 954 |
| GCF_001281305.1_ASM128130v1_01891 | gene532_longum_cbh | 954 |
| GCF_004333105.1_ASM433310v1_00827 | gene533_longum_cbh | 954 |
| GCF_000825125.1_BIB1401272845a.V1_01577 | gene534_longum_cbh | 954 |
| GCF_001264045.1_ASM126404v1_00901 | gene535_breve_cbh | 954 |
| GCF_004333715.1_ASM433371v1_00865 | gene536_longum_cbh | 954 |
| GCF_003460425.1_ASM346042v1_00476 | gene537_pseudocatenulatum_cbh | 951 |
| GCF_003466545.1_ASM346654v1_02010 | gene538_longum_cbh | 954 |
| GCF_004167905.1_ASM416790v1_00325 | gene539_animalis_cbh | 945 |
| GCF_001263865.1_ASM126386v1_00422 | gene540_animalis_cbh | 945 |
| GCF_004332645.1_ASM433264v1_00971 | gene541_longum_cbh | 954 |
| GCF_003466075.1_ASM346607v1_00839 | gene542_longum_cbh | 954 |

| | | |
|---|---|---|
| GCF_000411435.1_Bifi_brev_HPH0326_V1_01851 | gene543_breve_cbh | 954 |
| GCF_003437835.1_ASM343783v1_00205 | gene544_pseudocatenulatum_cbh | 951 |
| GCF_001042595.1_ASM104259v1_01090 | gene545_dentium_cbh | 951 |
| GCF_004333995.1_ASM433399v1_01000 | gene546_longum_cbh | 954 |
| GCF_003094855.1_ASM309485v1_01847 | gene547_longum_cbh | 954 |
| GCF_003094975.1_ASM309497v1_01002 | gene548_longum_cbh | 954 |
| GCF_005844335.1_ASM584433v1_00235 | gene549_pseudocatenulatum_cbh | 951 |
| GCF_004155165.1_ASM415516v1_00980 | gene550_pseudolongum_cbh | 945 |
| GCF_003095095.1_ASM309509v1_01329 | gene551_breve_cbh | 954 |
| GCF_000273525.1_PB_Bifi_bifi_NCIMB_41171_V1_00693 | gene552_bifidum_cbh | 951 |
| GCF_001685745.1_ASM168574v1_01087 | gene553_breve_cbh | 954 |
| GCF_000466545.1_ASM46654v1_01301 | gene554_breve_cbh | 954 |
| GCF_003466425.1_ASM346642v1_01995 | gene555_longum_cbh | 954 |
| GCF_003436545.1_ASM343654v1_00622 | gene556_pseudocatenulatum_cbh | 951 |
| GCF_001264105.1_ASM126410v1_01400 | gene557_breve_cbh | 954 |
| GCF_002832955.1_ASM283295v1_00853 | gene558_longum_cbh | 954 |
| GCF_004333575.1_ASM433357v1_00854 | gene559_longum_cbh | 954 |
| GCF_003390755.1_ASM339075v1_01258 | gene560_animalis_cbh | 945 |
| GCF_000022705.1_ASM2270v1_00858 | gene561_animalis_cbh | 945 |
| GCF_003436135.1_ASM343613v1_00870 | gene562_bifidum_cbh | 951 |
| GCF_003468385.1_ASM346838v1_00956 | gene563_adolescentis_cbh | 951 |
| GCF_004333015.1_ASM433301v1_00907 | gene564_longum_cbh | 954 |
| GCF_004333365.1_ASM433336v1_00935 | gene565_longum_cbh | 954 |
| GCF_003468005.1_ASM346800v1_01294 | gene566_longum_cbh | 954 |
| GCF_003466105.1_ASM346610v1_00469 | gene567_pseudocatenulatum_cbh | 951 |
| GCF_001990225.1_ASM199022v1_01012 | gene568_breve_cbh | 954 |
| GCF_001264015.1_ASM126401v1_00486 | gene569_animalis_cbh | 945 |
| GCF_002846815.1_ASM284681v1_00797 | gene570_pseudolongum_cbh | 945 |
| GCF_000940535.1_BPSEU7765_v1_00966 | gene571_pseudocatenulatum_cbh | 951 |

| | | |
|---|---|---|
| GCF_004334815.1_ASM433481v1_00908 | gene572_longum_cbh | 954 |
| GCF_004333205.1_ASM433320v1_01000 | gene573_longum_cbh | 954 |
| GCF_003952825.1_ASM395282v1_00968 | gene574_pseudocatenulatum_cbh | 951 |
| GCF_900445755.1_59024_E01_01561 | gene575_longum_cbh | 954 |
| GCF_003465205.1_ASM346520v1_01135 | gene576_adolescentis_cbh | 951 |
| GCF_001595465.1_ASM159546v1_00462 | gene577_longum_cbh | 954 |
| ref_00934 | gene578_pseudolongum_cbh | 945 |
| GCF_001725985.1_ASM172598v1_00931 | gene579_longum_cbh | 954 |
| GCF_002075875.1_Bbif1898B_01614 | gene580_longum_cbh | 954 |
| GCF_000196555.1_ASM19655v1_00851 | gene581_longum_cbh | 954 |
| GCF_004155855.1_ASM415585v1_01261 | gene582_pseudolongum_cbh | 945 |
| GCF_003990235.1_ASM399023v1_01819 | gene583_longum_cbh | 954 |
| GCF_002845845.1_ASM284584v1_00234 | gene584_bifidum_cbh | 951 |
| GCF_004334745.1_ASM433474v1_01082 | gene585_longum_cbh | 954 |
| GCF_003466335.1_ASM346633v1_01133 | gene586_adolescentis_cbh | 951 |
| GCF_004155795.1_ASM415579v1_01008 | gene587_pseudolongum_cbh | 945 |
| GCF_003466365.1_ASM346636v1_00921 | gene588_bifidum_cbh | 951 |
| GCF_002838505.1_ASM283850v1_01120 | gene589_breve_cbh | 954 |
| GCF_004333635.1_ASM433363v1_00951 | gene590_longum_cbh | 954 |
| GCF_004154695.1_ASM415469v1_00810 | gene591_animalis_cbh | 945 |
| GCF_004334365.1_ASM433436v1_00897 | gene592_longum_cbh | 954 |
| GCF_004156145.1_ASM415614v1_00449 | gene593_pseudolongum_cbh | 945 |
| GCF_003464925.1_ASM346492v1_01388 | gene594_pseudocatenulatum_cbh | 951 |
| GCF_003471505.1_ASM347150v1_00623 | gene595_pseudocatenulatum_cbh | 951 |
| GCF_003471295.1_ASM347129v1_00756 | gene596_pseudocatenulatum_cbh | 951 |
| GCF_000269965.1_ASM26996v1_01533 | gene597_longum_cbh | 954 |
| GCF_001020245.1_Bbif04v4_01402 | gene598_bifidum_cbh | 951 |
| GCF_003470545.1_ASM347054v1_00490 | gene599_pseudocatenulatum_cbh | 951 |
| GCF_001686005.1_ASM168600v1_01005 | gene600_pseudocatenulatum_cbh | 951 |

| | | |
|---|---|---|
| GCF_004334045.1_ASM433404v1_00954 | gene601_longum_cbh | 954 |
| GCF_001263835.1_ASM126383v1_00044 | gene602_animalis_cbh | 945 |
| GCF_002833135.1_ASM283313v1_00953 | gene603_longum_cbh | 954 |
| GCF_001685965.1_ASM168596v1_01500 | gene604_pseudocatenulatum_cbh | 951 |
| GCF_004919065.1_ASM491906v1_00430 | gene605_longum_cbh | 954 |
| GCF_001686025.1_ASM168602v1_00133 | gene606_pseudocatenulatum_cbh | 951 |
| GCF_000702865.1_ASM70286v1_01079 | gene607_adolescentis_cbh | 951 |
| GCF_000007525.1_ASM752v1_00497 | gene608_longum_cbh | 954 |
| GCF_003094795.1_ASM309479v1_00882 | gene609_bifidum_cbh | 951 |
| GCF_000247755.1_ASM24775v2_00253 | gene610_breve_cbh | 954 |
| GCF_004154995.1_ASM415499v1_01138 | gene611_pseudolongum_cbh | 945 |
| GCF_004334695.1_ASM433469v1_01014 | gene612_longum_cbh | 954 |
| GCF_000172535.1_Blac_1.0_01397 | gene613_animalis_cbh | 945 |
| GCF_002027265.1_ASM202726v1_00786 | gene614_breve_cbh | 954 |
| GCF_002108035.1_Bado703Bv1_01891 | gene615_adolescentis_cbh | 951 |
| GCF_004334785.1_ASM433478v1_01020 | gene616_longum_cbh | 954 |
| GCF_004333445.1_ASM433344v1_01027 | gene617_longum_cbh | 954 |
| GCF_003465745.1_ASM346574v1_01462 | gene618_longum_cbh | 954 |
| GCF_000219455.1_ASM21945v1_01045 | gene619_longum_cbh | 954 |
| GCF_004155475.1_ASM415547v1_00986 | gene620_pseudolongum_cbh | 945 |
| GCF_003438945.1_ASM343894v1_00025 | gene621_animalis_cbh | 945 |
| GCF_004334285.1_ASM433428v1_00911 | gene622_longum_cbh | 954 |
| GCF_900637215.1_49888_B01_01534 | gene623_longum_cbh | 954 |
| GCF_003473115.1_ASM347311v1_01070 | gene624_pseudocatenulatum_cbh | 951 |
| GCF_003471555.1_ASM347155v1_01793 | gene625_bifidum_cbh | 951 |
| GCF_003094755.1_ASM309475v1_01705 | gene626_bifidum_cbh | 951 |
| GCF_003464325.1_ASM346432v1_00907 | gene627_adolescentis_cbh | 951 |
| GCF_900157195.1_Bifido_02_v1_00872 | gene628_longum_cbh | 954 |
| GCF_004155235.1_ASM415523v1_00864 | gene629_pseudolongum_cbh | 945 |

| | | |
|---|---|---|
| GCF_001281425.1_ASM128142v1_00948 | gene630_breve_cbh | 954 |
| GCF_900637145.1_49569_E01_00933 | gene631_breve_cbh | 954 |
| GCF_000771145.1_DSM-20092_00545 | gene632_pseudolongum_cbh | 945 |
| GCF_003467335.1_ASM346733v1_01053 | gene633_adolescentis_cbh | 951 |
| GCF_004334545.1_ASM433454v1_00973 | gene634_longum_cbh | 954 |
| GCF_000741415.1_Bifste_00377 | gene635_adolescentis_cbh | 951 |
| GCF_000771725.1_DSM-20436_00887 | gene636_dentium_cbh | 951 |
| GCF_003463455.1_ASM346345v1_01205 | gene637_pseudocatenulatum_cbh | 951 |
| GCF_000771445.1_DSM-20438_00223 | gene638_pseudocatenulatum_cbh | 951 |
| GCF_003390735.1_ASM339073v1_01223 | gene639_bifidum_cbh | 951 |
| GCF_003464685.1_ASM346468v1_00180 | gene640_longum_cbh | 954 |
| GCF_001020375.1_Bbif03v4_01378 | gene641_bifidum_cbh | 951 |
| GCF_004334245.1_ASM433424v1_00945 | gene642_longum_cbh | 954 |
| GCF_003463505.1_ASM346350v1_01124 | gene643_pseudocatenulatum_cbh | 951 |
| GCF_004332725.1_ASM433272v1_00927 | gene645_longum_cbh | 954 |
| GCF_003370155.1_ASM337015v1_00520 | gene646_breve_cbh | 954 |
| GCF_002838685.1_ASM283868v1_00997 | gene647_breve_cbh | 954 |
| GCF_004333305.1_ASM433330v1_00851 | gene648_longum_cbh | 954 |
| GCF_001685945.1_ASM168594v1_01578 | gene649_breve_cbh | 954 |
| GCF_001686245.1_ASM168624v1_01450 | gene650_longum_cbh | 954 |
| GCF_004332625.1_ASM433262v1_00936 | gene651_longum_cbh | 954 |
| GCF_004334485.1_ASM433448v1_00962 | gene652_longum_cbh | 954 |
| GCF_004334345.1_ASM433434v1_00904 | gene653_longum_cbh | 954 |
| GCF_002076095.1_Bbif1886B_00569 | gene654_longum_cbh | 954 |
| GCF_002838365.1_ASM283836v1_01151 | gene655_breve_cbh | 954 |
| GCF_002108135.1_BadoLMG10733v1_00950 | gene656_adolescentis_cbh | 951 |
| GCF_003472095.1_ASM347209v1_01018 | gene657_adolescentis_cbh | 951 |
| GCF_003437995.1_ASM343799v1_00615 | gene658_longum_cbh | 954 |
| GCF_003465455.1_ASM346545v1_01357 | gene659_longum_cbh | 954 |

| | | |
|---|---|---|
| GCF_003467755.1_ASM346775v1_00249 | gene660_pseudocatenulatum_cbh | 951 |
| GCF_900103055.1_IMG-taxon_2636416037_annotated_assembly_01196 | gene661_longum_cbh | 954 |
| GCF_004333935.1_ASM433393v1_01001 | gene662_longum_cbh | 954 |
| GCF_004334075.1_ASM433407v1_00987 | gene663_longum_cbh | 954 |
| GCF_002221565.2_ASM222156v2_00398 | gene664_animalis_cbh | 945 |
| GCF_002871815.1_ASM287181v1_01711 | gene665_breve_cbh | 954 |
| GCF_002838625.1_ASM283862v1_00997 | gene666_breve_cbh | 954 |
| GCF_002838445.1_ASM283844v1_01189 | gene667_breve_cbh | 954 |
| GCF_003470125.1_ASM347012v1_00475 | gene668_pseudocatenulatum_cbh | 951 |
| GCF_000741625.1_Biflon_sub.sui_00938 | gene669_longum_cbh | 954 |
| GCF_002861455.1_ASM286145v1_01655 | gene670_breve_cbh | 954 |
| GCF_004803425.1_ASM480342v1_00864 | gene671_longum_cbh_1 | 1023 |
| GCF_000741485.1_Bifani_sub.ani_00750 | gene672_animalis_cbh | 945 |
| GCF_003437345.1_ASM343734v1_01709 | gene673_bifidum_cbh | 951 |
| GCF_004333125.1_ASM433312v1_00854 | gene674_longum_cbh | 954 |
| GCF_001406455.1_13414_6_8_00971 | gene675_adolescentis_cbh | 951 |
| GCF_000568955.1_ASM56895v1_00907 | gene676_breve_cbh | 954 |
| GCF_004333795.1_ASM433379v1_00965 | gene677_longum_cbh | 954 |
| GCF_004333535.1_ASM433353v1_00912 | gene678_longum_cbh | 954 |
| GCF_003463285.1_ASM346328v1_00175 | gene679_longum_cbh | 954 |
| GCF_004155625.1_ASM415562v1_00604 | gene680_pseudolongum_cbh | 945 |
| GCF_001685845.1_ASM168584v1_00850 | gene681_breve_cbh | 954 |
| GCF_004155615.1_ASM415561v1_00989 | gene682_pseudolongum_cbh | 945 |
| GCF_004156075.1_ASM415607v1_01285 | gene683_pseudolongum_cbh | 945 |
| GCF_002076015.1_Bbif1890B_01851 | gene684_longum_cbh | 954 |
| GCF_004155045.1_ASM415504v1_01204 | gene685_pseudolongum_cbh | 945 |
| GCF_001020255.1_Bbif02v4_01371 | gene686_bifidum_cbh | 951 |
| GCF_003473145.1_ASM347314v1_00706 | gene687_bifidum_cbh | 951 |

| | | |
|---|---|---|
| GCF_003465665.1_ASM346566v1_01887 | gene688_longum_cbh | 954 |
| GCF_900157155.1_Bifido_06_v1_00698 | gene689_longum_cbh | 954 |
| GCF_004155275.1_ASM415527v1_01175 | gene690_pseudolongum_cbh | 945 |
| GCF_003467785.1_ASM346778v1_00249 | gene691_pseudocatenulatum_cbh | 951 |
| GCF_001685725.1_ASM168572v1_00700 | gene692_breve_cbh | 954 |
| GCF_001685685.1_ASM168568v1_00196 | gene693_bifidum_cbh | 951 |
| GCF_005844345.1_ASM584434v1_01233 | gene694_longum_cbh | 954 |
| GCF_002108045.1_BadoAD28v1_01562 | gene695_adolescentis_cbh | 951 |
| GCF_000421385.1_ASM42138v1_01339 | gene696_longum_cbh | 954 |
| GCF_002075965.1_Bbif1892B_00509 | gene697_adolescentis_cbh | 951 |
| GCF_003473025.1_ASM347302v1_00990 | gene698_pseudocatenulatum_cbh | 951 |
| GCF_002108165.1_BadoLMG188971_01806 | gene699_adolescentis_cbh | 951 |
| GCF_004156195.1_ASM415619v1_01630 | gene700_pseudolongum_cbh | 945 |
| GCF_002838585.1_ASM283858v1_01039 | gene701_breve_cbh | 954 |
| GCF_004333565.1_ASM433356v1_00964 | gene702_longum_cbh | 954 |
| GCF_002833315.1_ASM283331v1_00963 | gene703_longum_cbh | 954 |
| GCF_002914815.1_ASM291481v1_00105 | gene704_animalis_cbh | 945 |
| GCF_003342655.1_ASM334265v1_00891 | gene705_longum_cbh | 954 |
| GCF_003437775.1_ASM343777v1_00031 | gene706_adolescentis_cbh | 951 |
| GCF_003094645.1_ASM309464v1_01540 | gene707_bifidum_cbh | 951 |
| GCF_003468265.1_ASM346826v1_00125 | gene708_longum_cbh | 954 |
| GCF_001685805.1_ASM168580v1_01277 | gene709_breve_cbh | 954 |
| GCF_002076105.1_Bbif1887B_00694 | gene710_bifidum_cbh | 951 |
| GCF_000816205.1_ASM81620v1_00862 | gene711_animalis_cbh | 945 |
| GCF_001263935.1_ASM126393v1_00019 | gene712_breve_cbh | 954 |
| GCF_001020335.1_Bbif08v4_01448 | gene713_bifidum_cbh | 951 |
| GCF_003437755.1_ASM343775v1_01007 | gene714_adolescentis_cbh | 951 |
| GCF_002838745.1_ASM283874v1_00997 | gene715_breve_cbh | 954 |
| GCF_003030905.1_ASM303090v1_01080 | gene716_adolescentis_cbh | 951 |

| | | |
|---|---|---|
| GCF_001686065.1_ASM168606v1_01219 | gene717_pseudocatenulatum_cbh | 951 |
| GCF_002846715.1_ASM284671v1_00484 | gene718_pseudolongum_cbh | 945 |
| GCF_004155425.1_ASM415542v1_00185 | gene719_pseudolongum_cbh | 945 |
| GCF_002838525.1_ASM283852v1_00968 | gene720_breve_cbh | 954 |
| GCF_001686205.1_ASM168620v1_00330 | gene721_longum_cbh | 954 |
| GCF_003471325.1_ASM347132v1_00243 | gene722_pseudocatenulatum_cbh | 951 |
| GCF_004155695.1_ASM415569v1_00197 | gene723_pseudolongum_cbh | 945 |
| GCF_001263975.1_ASM126397v1_00603 | gene724_animalis_cbh | 945 |
| GCF_003470615.1_ASM347061v1_01793 | gene725_bifidum_cbh | 951 |
| GCF_005405825.1_ASM540582v1_00764 | gene726_moukalabense_cbh | 951 |
| GCF_002833055.1_ASM283305v1_00866 | gene727_longum_cbh | 954 |
| GCF_000265095.1_ASM26509v1_00853 | gene728_bifidum_cbh | 951 |
| GCF_000730045.1_ASM73004v1_01187 | gene729_longum_cbh | 954 |
| GCF_005844715.1_ASM584471v1_00835 | gene730_longum_cbh | 954 |
| GCF_004333115.1_ASM433311v1_00900 | gene731_longum_cbh | 954 |
| GCF_000730125.1_ASM73012v1_00390 | gene732_longum_cbh | 954 |
| GCF_004324325.1_ASM432432v1_02023 | gene733_longum_cbh | 954 |
| GCF_004334155.1_ASM433415v1_01060 | gene734_longum_cbh | 954 |
| GCF_004803425.1_ASM480342v1_02124 | gene735_longum_cbh | 954 |
| GCF_002838645.1_ASM283864v1_00996 | gene736_breve_cbh | 954 |
| GCF_004155595.1_ASM415559v1_01034 | gene737_pseudolongum_cbh | 945 |
| GCF_003459475.1_ASM345947v1_00555 | gene738_pseudocatenulatum_cbh | 951 |
| GCF_000261205.1_Blongum22Bv1.0_01260 | gene739_longum_cbh | 954 |
| GCF_003465095.1_ASM346509v1_01381 | gene740_longum_cbh | 954 |
| GCF_004333515.1_ASM433351v1_00917 | gene741_longum_cbh | 954 |
| GCF_003094915.1_ASM309491v1_01025 | gene742_animalis_cbh | 945 |
| GCF_003094935.1_ASM309493v1_00004 | gene743_longum_cbh | 954 |
| GCF_004334355.1_ASM433435v1_00897 | gene744_longum_cbh | 954 |
| GCF_000825105.1_BIB1401242951.V1_01574 | gene745_longum_cbh | 954 |

| | | |
|---|---|---|
| GCF_001010915.1_ASM101091v1_01480 | gene746_adolescentis_cbh | 951 |
| GCF_004333625.1_ASM433362v1_00880 | gene747_longum_cbh | 954 |
| GCF_003437095.1_ASM343709v1_00383 | gene748_longum_cbh | 954 |
| GCF_004332855.1_ASM433285v1_00966 | gene749_longum_cbh | 954 |
| GCA_001576865.1_ASM157686v1_01088 | gene750_bifidum_cbh | 951 |
| GCF_003465365.1_ASM346536v1_00951 | gene751_longum_cbh | 954 |
| GCF_004155645.1_ASM415564v1_01190 | gene753_pseudolongum_cbh | 945 |
| GCF_000825085.1_BIC1401212621b.V1_01604 | gene754_longum_cbh | 954 |
| GCF_001685885.1_ASM168588v1_00231 | gene755_breve_cbh | 954 |
| GCF_003671995.1_ASM367199v1_00881 | gene756_animalis_cbh | 945 |
| GCF_003465135.1_ASM346513v1_00583 | gene757_pseudocatenulatum_cbh | 951 |
| GCF_000021185.1_ASM2118v1_00504 | BSH-T2_Listeria_monocytogenes_HCC23 | 1011 |
| GCF_000072485.1_ASM7248v1_02093 | BSH-T6_Stenotrophomonas_maltophilia_K279a | 1053 |
| GCF_000146405.1_ASM14640v1_00655 | BSH-T2_Streptococcus_equinus_ATCC_700338 | 981 |
| GCF_000146525.1_ASM14652v1_00812 | BSH-T2_Streptococcus_gallolyticus_subsp._gallolyticus_TX20005 | 981 |
| GCF_000154065.1_ASM15406v1_01793 | BSH-T1_Dorea_longicatena_DSM_13814 | 978 |
| GCF_000154245.1_ASM15424v1_02114 | BSH-T1_Clostridium_sp._L2-50 | 975 |
| GCF_000154445.1_ASM15444v1_02475 | BSH-T0_Intestinibacter_bartlettii_DSM_16795 | 987 |
| GCF_000154525.1_ASM15452v1_02320 | BSH-T6_Bacteroides_stercoris_ATCC_43183 | 1059 |
| GCF_000154545.1_ASM15454v1_02459 | BSH-T1_Clostridium_sp._SS2/1 | 972 |
| GCF_000154845.1_ASM15484v1_00317 | BSH-T5_Bacteroides_coprocola_DSM_17136 | 1104 |
| GCF_000154985.1_ASM15498v1_00524 | BSH-T2_Streptococcus_infantarius_subsp._infantarius_ATCC_BAA-102 | 978 |
| GCF_000156035.2_ASM15603v2_00266 | BSH-T7_Tyzzerella_nexilis_DSM_1787 | 978 |
| GCF_000156655.1_ASM15665v1_00072 | BSH-T1_Holdemanella_biformis_DSM_3989 | 951 |
| GCF_000158195.2_Fuso_mor_ATCC9817_V2_01081 | BSH-T5_Fusobacterium_mortiferum_ATCC_9817 | 1101 |
| GCF_000158375.2_Clostridium_sp_7_2_43FAA_V2_02978 | BSH-T0_Clostridium_sp._7_2_43FAA | 993 |
| GCF_000159415.1_ASM15941v1_01864 | BSH-T3_Lactobacillus_ultunensis_DSM_16047 | 978 |
| GCF_000161915.2_Lacto_cris_MV-1A-US_V2_00115 | BSH-T3_Lactobacillus_crispatus_MV-1A-US | 978 |

| | | |
|---|---|---|
| GCF_000168475.1_ASM16847v1_02573 | BSH-T2_Listeria_monocytogenes_FSL_J2-071 | 1011 |
| GCF_000168635.2_ASM16863v2_02117 | BSH-T2_Listeria_monocytogenes_J0161 | 978 |
| GCF_000173795.1_ASM17379v1_02246 | BSH-T1_Catenibacterium_mitsuokai_DSM_15897 | 975 |
| GCF_000179335.1_ASM17933v1_01074 | BSH-T1_Peptoniphilus_sp._oral_taxon_836_str._F0141 | 978 |
| GCF_000182605.1_ASM18260v1_01543 | BSH-T0_Clostridium_butyricum_E4_str._BoNT_E_BL5262 | 990 |
| GCF_000187895.1_ASM18789v1_00984 | BSH-T5_Bacteroides_plebeius_DSM_17135 | 1083 |
| GCF_000382085.1_ASM38208v1_00272 | BSH-T1_[Eubacterium]_siraeum_DSM_15702 | 975 |
| GCF_003019655.1_ASM301965v1_00418 | BSH-T5_Fusobacterium_varium_ATCC_27725 | 1104 |

**Table S3 The clade-specific core genes of *B. pseudolongum***

| **Gene** | **Annotation** | **A** | **B** | **C** | **D** |
|---|---|---|---|---|---|
| group_1132 | hypothetical protein | 1 | 0 | 0 | 0 |
| group_1946 | FHA domain protein | 1 | 0 | 0 | 0 |
| group_1947 | 5-formyltetrahydrofolate cyclo-ligase family protein | 1 | 0 | 0 | 0 |
| group_1948 | hypothetical protein | 1 | 0 | 0 | 0 |
| group_1949 | tRNA pseudouridine synthase B | 1 | 0 | 0 | 0 |
| group_1951 | hypothetical protein | 1 | 0 | 0 | 0 |
| group_2841 | hypothetical protein | 1 | 0 | 0 | 0 |
| group_2842 | Ribonuclease HI | 1 | 0 | 0 | 0 |
| group_2843 | hypothetical protein | 1 | 0 | 0 | 0 |
| group_2846 | pheromone autoinducer 2 transporter | 1 | 0 | 0 | 0 |
| group_2847 | hypothetical protein | 1 | 0 | 0 | 0 |
| group_2848 | hypothetical protein | 1 | 0 | 0 | 0 |
| group_2851 | hypothetical protein | 1 | 0 | 0 | 0 |
| group_2852 | hypothetical protein | 1 | 0 | 0 | 0 |
| group_2866 | Dephospho-CoA kinase | 1 | 0 | 0 | 0 |
| group_2867 | DNA utilization protein GntX | 1 | 0 | 0 | 0 |
| group_2868 | Transcriptional regulator LytR | 1 | 0 | 0 | 0 |

| | | | | | |
|---|---|---|---|---|---|
| group_2869 | ESX-1 secretion system protein EccCa1 | 1 | 0 | 0 | 0 |
| group_2870 | Beta-hexosaminidase | 1 | 0 | 0 | 0 |
| group_2872 | Sensor histidine kinase DesK | 1 | 0 | 0 | 0 |
| group_2873 | hypothetical protein | 1 | 0 | 0 | 0 |
| group_2876 | Glycerate 2-kinase | 1 | 0 | 0 | 0 |
| cdaR | Carbohydrate diacid regulator | 1 | 0 | 0 | 0 |
| group_2879 | hypothetical protein | 1 | 0 | 0 | 0 |
| group_2880 | hypothetical protein | 1 | 0 | 0 | 0 |
| group_2881 | hypothetical protein | 1 | 0 | 0 | 0 |
| group_2882 | cell division protein FtsQ | 1 | 0 | 0 | 0 |
| group_2884 | DivIVA protein | 1 | 0 | 0 | 0 |
| group_2885 | LL-diaminopimelate aminotransferase | 1 | 0 | 0 | 0 |
| group_2886 | Methionine import ATP-binding protein MetN | 1 | 0 | 0 | 0 |
| group_2901 | hypothetical protein | 1 | 0 | 0 | 0 |
| group_2903 | Resuscitation-promoting factor Rpf2 precursor | 1 | 0 | 0 | 0 |
| group_2905 | hypothetical protein | 1 | 0 | 0 | 0 |
| group_2909 | HTH-type transcriptional regulator DegA | 1 | 0 | 0 | 0 |
| group_2911 | MarR family protein | 1 | 0 | 0 | 0 |
| group_2913 | Bacterial type II secretion system protein F domain protein | 1 | 0 | 0 | 0 |
| group_2914 | hypothetical protein | 1 | 0 | 0 | 0 |
| group_2916 | L-glyceraldehyde 3-phosphate reductase | 1 | 0 | 0 | 0 |
| group_2920 | hypothetical protein | 1 | 0 | 0 | 0 |
| group_2923 | hypothetical protein | 1 | 0 | 0 | 0 |
| group_2924 | Holo-[acyl-carrier-protein] synthase | 1 | 0 | 0 | 0 |
| group_2925 | hypothetical protein | 1 | 0 | 0 | 0 |
| group_887 | hypothetical protein | 1 | 0 | 0 | 0 |
| group_2164 | hypothetical protein | 0 | 1 | 0 | 0 |
| group_3445 | hypothetical protein | 0 | 1 | 0 | 0 |
| group_2818 | Adenosine monophosphate-protein transferase SoFic | 0 | 0 | 1 | 0 |

| | | | | | |
|---|---|---|---|---|---|
| group_2824 | Nucleotidyltransferase domain protein | 0 | 0 | 1 | 0 |
| group_15 | hypothetical protein | 0 | 0 | 0 | 1 |
| group_1754 | hypothetical protein | 0 | 0 | 0 | 1 |
